# Active Slingshot Geometry Site on Single-Atom La Catalyst Largely Promotes Oxidative Methane Coupling

**DOI:** 10.1021/acscentsci.5c01016

**Published:** 2025-09-20

**Authors:** Lizhuo Wang, Liwei Cao, Ang Li, Wenjie Yang, Wei Li, Xiaozhou Liao, Xiaodong Han, Jun Huang

**Affiliations:** † Laboratory for Catalysis Engineering, School of Chemical and Biomolecular Engineering, Sydney Nano Institute, 4334 The University of Sydney, Sydney, NSW 2006, Australia; ‡ Beijing Key Laboratory of Microstructure and Property of Advanced Materials, 12496 Beijing University of Technology, Beijing 100124, China; § Australian Centre for Microscopy & Microanalysis and School of Aerospace, Mechanical and Mechatronic Engineering, The University of Sydney, Sydney, NSW 2006, Australia; ∥ Department of Materials Science and Engineering, 255310 Southern University of Science and Technology, Shenzhen 518055, China

## Abstract

Oxidative methane coupling (OCM) has long been deemed a promising route for the direct conversion of methane to valuable ethylene. Despite its potential and many progresses, OCM’s industrial implementation has been hampered by low C_2_ yields and insufficient understanding of the reaction mechanism for catalyst design. In this study, we present a surface geometric modification strategy to enhance OCM performance. Single La atoms incorporated onto MgO surface (SA-La/MgO) form a unique La–O–Mg “slingshot” geometry. This configuration, driven by the large atomic radius of La and its valency mismatch with Mg, significantly activates surface lattice oxygen. These activated oxygen species initiate the OCM by reacting with methane, while the resulting oxygen vacancies are rapidly replenished by dioxygen, sustaining active oxygen supply and preserving the structural integrity of single La atoms. These processes are realized by state-of-the-art *in situ* environmental electron microscopy and electron energy loss spectroscopy. Remarkably, the La–O–Mg “slingshot” geometry doubles C_2_ yields and significantly elevates the turnover frequency of SA-La/MgO compared to La_2_O_3_ particles on MgO, which lacks such active oxygen species. This work discovers a new mechanism for largely enhancing the OCM performance, emphasizing the importance of atomic-scale geometric and electronic modifications in catalyst design.

## Graphical abstract

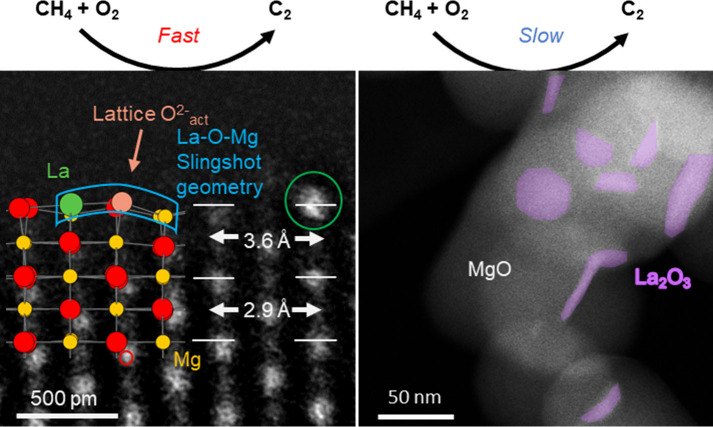

## 1 Introduction

The direct oxidative coupling of methane (OCM) to produce valuable C_2_ hydrocarbons represents a transformative approach to leverage natural gas or biogas as a clean or sustainable feedstock for the chemical industry. As a one-step process, OCM offers a compelling alternative to the conventional multistep indirect conversion of methane, which involves energy-intensive processes such as reforming to syngas, methanol synthesis, and subsequent conversion to C_2_ hydrocarbons. By eliminating intermediate steps, OCM not only enhances cost and energy efficiency but also significantly reduces carbon emissions, positioning it as a cornerstone for sustainable chemical production. Despite its potential, the widespread adoption of OCM has been hindered by challenges in achieving high C_2_ yields, primarily due to the trade-off between methane conversion and C_2_ selectivity, as well as the overoxidation of hydrocarbons to CO_*x*_.^1–8^ Overcoming these limitations requires a profound understanding of the catalytic mechanisms and active sites involved, which remain elusive due to the complexity of the reaction and the dynamic nature of the catalysts under operating conditions.

Recent advancements in catalyst development have identified promising materials, such as Mn/Na_2_WO_4_/SiO_2_ and Ba_2_TiO_4_, which exhibit notable activities and stabilities during OCM.^3^ Additionally, metal-ion-doped oxides, particularly Li- and La-doped variants, have garnered significant attention for their potential to enhance C_2_ selectivity.^9^ Among these, La-based catalysts stand out for their ability to improve C_2_ hydrocarbon selectivity in the OCM process,^10^ yet their performance () is still constrained by the intrinsic challenge of balancing methane conversion with selectivity.^11–13^ A critical barrier to progress lies in the lack of atomic-scale insights into the structural and compositional evolution of active sites under the reaction conditions, compounded by the exothermic nature of the OCM, which induces dynamic changes in catalyst behavior. Traditional mechanistic models, such as the widely accepted role of ^•^O and ^•^CH_3_ radicals,^1,14^ remain inadequately supported by experimental evidence, underscoring the need for advanced characterization techniques to unravel the intricacies of the OCM process.^15,16^

The advent of *in situ* transmission electron microscopy (TEM) coupled with electron energy loss spectroscopy (EELS) has opened new frontiers in catalyst research, enabling unprecedented resolution and sensitivity in observing geometric and compositional changes at the atomic scale. These techniques provide a unique opportunity to probe the dynamic behavior of catalysts under realistic operating conditions, offering critical insights into the nature of active sites and reaction mechanisms.^17–20^

In the present research, La-doped MgO catalysts, a well-established doped oxide, are chosen as the model catalyst for investigating the performance of the OCM over them. Through a variety of *in situ* characterizations, the findings confirm that there are two types of doped oxides: La_2_O_3_ particle-doped MgO (PA-La/MgO) and single-atom La in MgO (SA-La/MgO), each contributing fundamentally different active sites and exhibiting a distinct OCM performance. Despite their similar chemical compositions, OCM follows different reaction mechanisms over these two types of doped oxides, resulting in significant differences in catalytic performance. This work underscores the importance of detailed structural and mechanistic studies under working conditions, paving the way for the rational design of next-generation OCM catalysts with enhanced efficiency and sustainability.

## 2 Experimental Section

### 2.1 Catalyst Synthesis

The synthesis of the catalysts follows the Pechini sol–gel method^21^ or impregnation method. For the Pechini sol–gel method, 12.82 g of Mg­(NO_3_)_2_·6H_2_O (Sigma-Aldrich) and 0.06 g of La­(NO_3_)_3_·6H_2_O are dissolved in 25 mL of deionized water. Then, 9.60 g of citric acid and 3.10 g of ethylene glycol are added in sequence. The resultant solution is stirred rigorously with a magnetic stirrer and heated at 100 °C. Then, heating is continued until the gel is generated and solid precursor is formed. The resultant solid precursor is then dehydrated in a 120 °C oven overnight. The dried solid is crushed in a mortar before calcined in a muffle furnace in static air at 950 °C for 12 h. The synthesized white power is denoted as SA-La/MgO. The nominal La content of it is 1 w%. For comparison, the particle La_2_O_3_ loaded MgO was synthesized via the impregnation method. The intrinsic MgO is synthesized via the published method.^22^ Specifically, 1.5 M magnesium nitrate solution was added to the Na_2_CO_3_ (1M) solution dropwise. The volume between the magnesium nitrate solution and the Na_2_CO_3_ solution was 1:1.5. The pH value of the mixture was adjusted to 10 by 10 M NaOH. The obtained mixture was consequently heated at 80 °C overnight in oven. The resultant gel was filtrated and washed by deionized water several times and then dried in a 120 °C oven for 10 h before calcination in an 850 °C muffle furnace for 5 h in air. The resultant product is the substrate MgO. Then, 1 g of MgO, 0.15 g of La­(NO_3_)_3_·H_2_O and 25 mL of water were added into a baker, rigorously stirred for 30 min, and then evaporated the water on a heat plate. The resultant solid is then calcined in muffle furnace at 950 °C for 12 h. The resulted catalyst is denoted as PA-La/MgO and the nominal La content is 5 w%.

### 2.2 Catalyst Characterization

#### 2.2.1 *Ex Situ* Characterization

The X-ray diffraction (XRD) pattern was collected over PANalytical Xpert Pro powder diffractometer (45 kV and 40 mA) using Cu Ka radiation (*l* = 1.5405 Å). The scanning range for all of the samples was 10° to 80°. The X-ray photoelectron spectroscopy (XPS) results were collected on Thermo Fisher K Alpha X-ray photoelectron spectrometer with a monochromated Al Kα X-ray was applied as the radiation source. The range of scan was 50 eV. High resolution high angle annular dark field (HAADF) images and integrated differential phase contrast (iDPC) images were taken on the FEI Themis Z was equipped with a probe and image spherical aberration corrector on 300 kV.

#### 2.2.2 *In Situ* Characterization and Performance Test

Environmental transmission electron microscopy (ETEM) images, EELS, and *in situ* diffuse reflectance inferred Fourier transformation spectroscopy (DRIFTS) are acquired to identify the catalyst behaviors during reaction.

The *in situ* ETEM images and EELS were acquired on FEI ETEM operated under 300 kV. The experiment was carried out in O_2_, CH_4_, or the mixture of O_2_ and CH_4_ (1:4 v/v) gaseous environment. The temperature is controlled by the *in situ* heating MEMS chip obtained from DENSESolution. The *in situ* TEM-EELS spectra were taken from the edge region on the catalyst along with *in situ* images. The TEM-EELS was acquired at 27 000× magnification, and the irradiation area was around 100 um^2^. EELS signal was captured from the entire illuminated field. The commercial electron prism provided by Gatan Inc. was applied for acquiring the EELS spectra. To avoid beam-induced sample damage, the electron dosage was controlled to be less than 650 e^–^/(Å^2^·s).

The *in situ* DRIFTS test was carried out in the Nicolet iS50 provided by ThermoFisher Scientific equipped with a Harrick *in situ* reactor. The window of the reactor is made by GeZn. In the experiment, proper amount of sample was put into the reactor with the support of the stainless-steel mesh. The catalyst was *in situ* heated at 500 °C in the nitrogen flux (60 mL/min) to remove the adsorbed water. The catalyst was then cooled to 100 °C before the test being proceeded. During the measurement, the catalyst in the corresponding gas flux was heated up with steps, and the interval of these steps was 50 °C. The liquid nitrogen cooled MCT-A detector was applied with the scan number as 128 times. The resolution of the spectrum was 4 cm^–1^. The exhaust of the *in situ* FTIR was linked with a MicroGC (Varian CP-4900) or Agilent GC 7890B to analyze the product of the reaction.

## 3 Results and Discussion

HAADF scanning transmission electron microscopy (STEM) images displayed over Figure 1a–c revealed the localized structure of the prepared samples. The lattice distance measured on intrinsic MgO shown in Figure 1a is 0.42 nm, which corresponds to the (100) facet of MgO (PDF#45-0946), as also indicated by the XRD measurement in Figure 1d and Figure S1. In Figure 1b, which depicts the image of PA-La/MgO, the brighter contrast of the particle on the left side of the image indicates that the particle is composed of heavier elements, specifically a La_2_O_3_ particle on MgO. The low magnification HAADF-STEM image for PA-La/MgO (Figure S2b) indicates that La species are in the form of a flat sheet with an average thickness of 7.45 ± 1.43 nm. The XRD measurement of a particle size calculated via the Scherrer equation is approximately 18.3 nm^23^ in Figure 1d. As for the SA-La/MgO shown in Figure 1c, the HAADF-STEM image and its contrast profile indicate that single-site lanthanum (marked with a red circle) can be distinguished on top of the MgO surface. No La_2_O_3_ particles were observed from either STEM or XRD. The single La atom substitutes for the Mg atom within the MgO crystal lattice. Notably, these distinct brighter dots are solely observed in the surface region of MgO. The low magnification image of SA-La/MgO (Figure S2a) indicates no obvious La species aggregation, which corresponds to the STEM EDS mapping (Figure S3) results which indicate that La is well dispersed on MgO without aggregation on SA-LaMgO. Therefore, isolated La atoms have been generated on the surface of SA-La/MgO. This is also confirmed by the XPS measurements in Figure 1e,f, Figure S4, and Figure S5. Based on the above characterizations, both the proposed particle and single-atom doping were achieved during the synthesis process, and the structures of these two catalysts are illustrated in Figure 1h,i. In the OCM tests conducted within the *in situ* DRIFTS system equipped with an online GC, all the prepared oxides MgO, PA-La/MgO, and SA-La/MgO exhibited catalytic activity. PA-La/MgO showed only a marginal improvement compared to undoped MgO. In contrast, SA-La/MgO demonstrated significantly enhanced OCM activity, indicating the effectiveness of the SA La catalyst.

**Figure 1:**
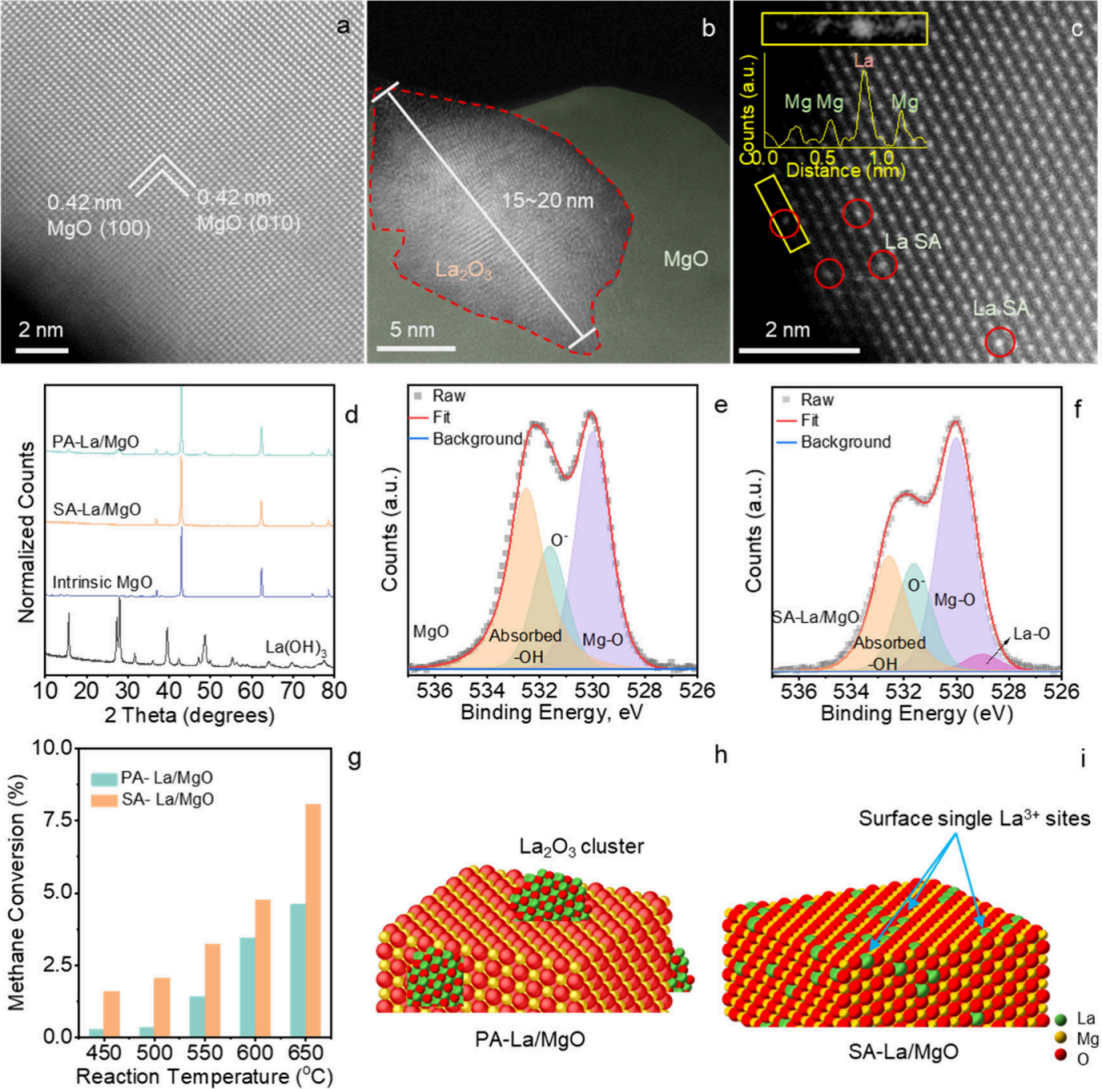
HAADF-STEM image for intrinsic MgO (a), PA-La/MgO (b), and SA-La/MgO (c); XRD pattern of PA-La/MgO, SA-La/MgO, intrinsic MgO and La­(OH)_3_ (d); O 1s XPS result of intrinsic MgO (e) and SA-La/MgO (f); methane conversion at different temperature of PA-La/MgO and SA-La/MgO based on DRIFTS product analysis (g); illustration of the structure of PA-La/MgO (h) and SA-La/MgO (i).

The apparent activation energy of methane conversion over SA-La/MgO, PA-La/MgO, and intrinsic MgO was computed. As depicted in Figure S7, the apparent methane activation energy over SA-La/MgO is 55.5 ± 3.9 kJ/mol, which is notably lower than that over PA-La/MgO (97.9 ± 18.0 kJ/mol) or intrinsic MgO (204.3 ± 13.5 kJ/mol). This indicates that methane is much more readily activated on SA-La/MgO compared to PA-La/MgO and intrinsic MgO. The significant disparity in the apparent activation energy implies that the methane activation mechanism over SA-La/MgO diverges from that over PA-La/MgO and MgO, thereby leading to markedly different OCM performance.

### 3.1 Geometry and Composition of Active Sites on SA-La/MgO for the *In Situ* Generation of O Defects at Reaction Conditions

Figure 2a presents the HAADF-STEM image of SA-La/MgO. The distinctively brighter dots represent atomically distributed La atoms within the MgO crystal lattice. When the region of the La atom is further zoomed in as depicted in Figure 2b, it is evident that the lattice distance between the La atom and the Mg atom is 3.6 Å, whereas the lattice distance of MgO is 2.9 Å. To further elaborate on the nature of the vicinity of a single atom of La, the SA-La/MgO crystal has been simulated using the CrystalMaker software package. After relaxation, as depicted by the color model in Figure 2b, the positions of the La atom and the Mg atom correspond precisely to their actual positions. The lattice oxygen adjacent to the single atom of La, as shown in Figure 2b, is compelled to deviate from its original location. The substitution of Mg (0.072 nm)^24^ in the lattice by La with a larger radius (0.103 nm)^25^ exerts pressure on the adjacent oxygen atoms, causing them to protrude outward from the crystal surface. Consequently, it results in a “slingshot” type of La–O–Mg coordination geometry. The STEM-iDPC image shown in Figure S8 revealed the surface lattice of O^2–^ adjacent to La ions was displaced from its original position. This direct observation double confirms the formation of “slingshot” type of La–O–Mg coordination geometry. Given that the displacement of lattice O atoms on the crystal has been reported to confer better catalytic properties,^26^ the “slingshot” geometry gives rise to the generation of a greater amount of surface-active oxygen. In contrast, as shown in Figure S9, intrinsic MgO exhibited a stable surface without the emergence of the “slingshot” geometry, further confirming that this unique structure originates from the effect of La^3+^.

**Figure 2:**
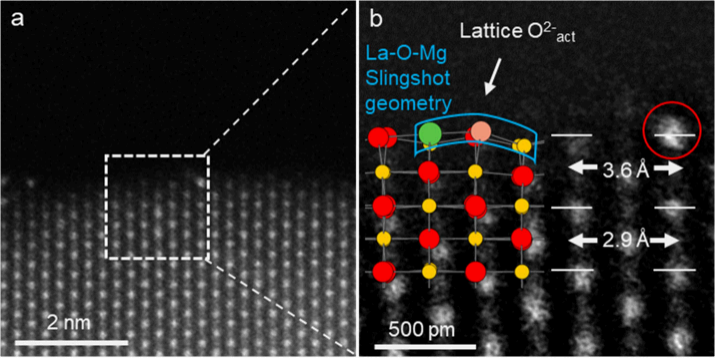
(a) HAADF-STEM image of SA-La/MgO and (b) the zoomed-in image of panel (a); simulation of SA-La/MgO catalyst after relaxation was also added for comparison (where green ball represents La, yellow ball represents Mg, red ball represents O, and pink ball represents active lattice O).

Moreover, the La atom (La^3+^) in oxides has a higher valency than that of the Mg atom (Mg^2+^). The doped La^3+^ in the MgO lattice alters the localized electron structure of the surface La–O–Mg geometry.^27^ This is anticipated to further enhance the activity of lattice O. Additionally, the size difference between La and Mg affects the bonding strengths of the lattice O atom to La and Mg, thereby modifying the electronic structure of lattice O to improve its activity. Consequently, the O anion on the surface La–O–Mg geometry is hypothesized to be the highly active species (lattice O^2–^_act._) that can contribute reactivity to the OCM reactions.

Methane has been introduced to SA-La/MgO catalysts for an *in situ* ETEM investigation of the working geometry and composition on the surface. At ambient temperature in the presence of methane, all La (the largest bright dot), Mg (smaller yellow dots), and O (dots similar to Mg) ions are well-oriented on the surface, as depicted in Figure 3a. Moreover, no structural transformation or surface defects are observed on the catalyst under the TEM electron beam even when the catalyst is heated to 300 °C (Figure S10). When the temperature reaches 400 °C, as presented in Figure 3b, the dim sites, highlighted by red circles, are identified on the surface of the catalyst. Given that the simulated TEM image shows a lower contrast of oxygen vacancies on MgO (Figure S11), the dim sites observed in Figure 3b indicate the generation of oxygen vacancies on the catalyst surface. Specifically, the surface-active lattice oxygen from La–O–Mg has been consumed during the reaction with methane. The disturbed region on SA-La/MgO was observed when an exposed sample was exposed in methane at 600 °C (Figure 3c), suggesting that a superstructure may form in this area. This phenomenon may be attributed to the accumulation of surface oxygen vacancies, a phenomenon that has been previously observed on different metal oxides.^28,29^ For comparison, the *in situ* ETEM image of intrinsic MgO in Figure 3d does not show oxygen vacancies after the introduction of methane at 600 °C. Additionally, when SA-La/MgO is exposed only to O_2_, no oxygen vacancies are detected even when the sample is heated to 700 °C under *in situ* ETEM investigation (Figures S12–S15), confirming that the reaction with methane results in the generation of these oxygen vacancies. Therefore, the La single atom in MgO induces the “slingshot” La–O–Mg geometry and the valency difference, providing highly active lattice O_2_^–^ for methane conversion.

**Figure 3:**
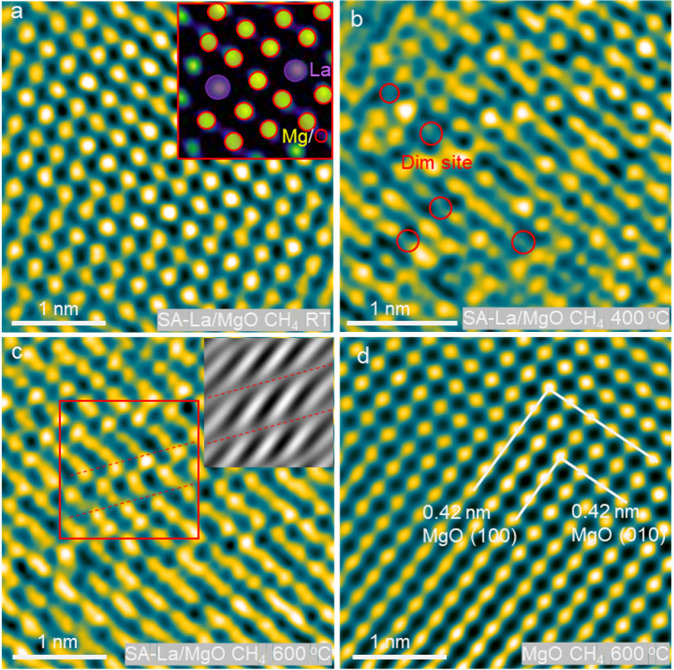
*In situ* ETEM image of SA-La/MgO in methane atmosphere at room temperature and the illustration of La single atom on SA-La/MgO (a); *in situ* ETEM image of SA-La/MgO in methane atmosphere at 400 °C (b) and at 600 °C (c). The IFFT image of the selected area (red square) in panel (a) is also provided, and the red dashed line in panel (c) indicates the disturbed regions. *In situ* ETEM image of intrinsic MgO in methane atmosphere at 600 °C (d).

To further validate this mechanism, *in situ* ETEM was performed under an alternating CH_4_ and O_2_ atmosphere. As shown in Figure 4a, the dim sites emerged on SA-La/MgO at 400 °C under a CH_4_ environment, indicating the formation of oxygen vacancies. Upon switching the gas environment from CH_4_ to O_2_, most of the dim sites disappeared. We further increased the temperature to 600 °C and performed gas environment alternation. Similar behavior was observed in the same area at 600 °C (Figure 4c,d), and the quantitative evolution of dim site density under different conditions is presented in Figure S16. These results reveal an oscillation in the number of dim sites depending on the gas environment, confirming the reversible generation and replenishment of oxygen vacancies during the reaction.

**Figure 4:**
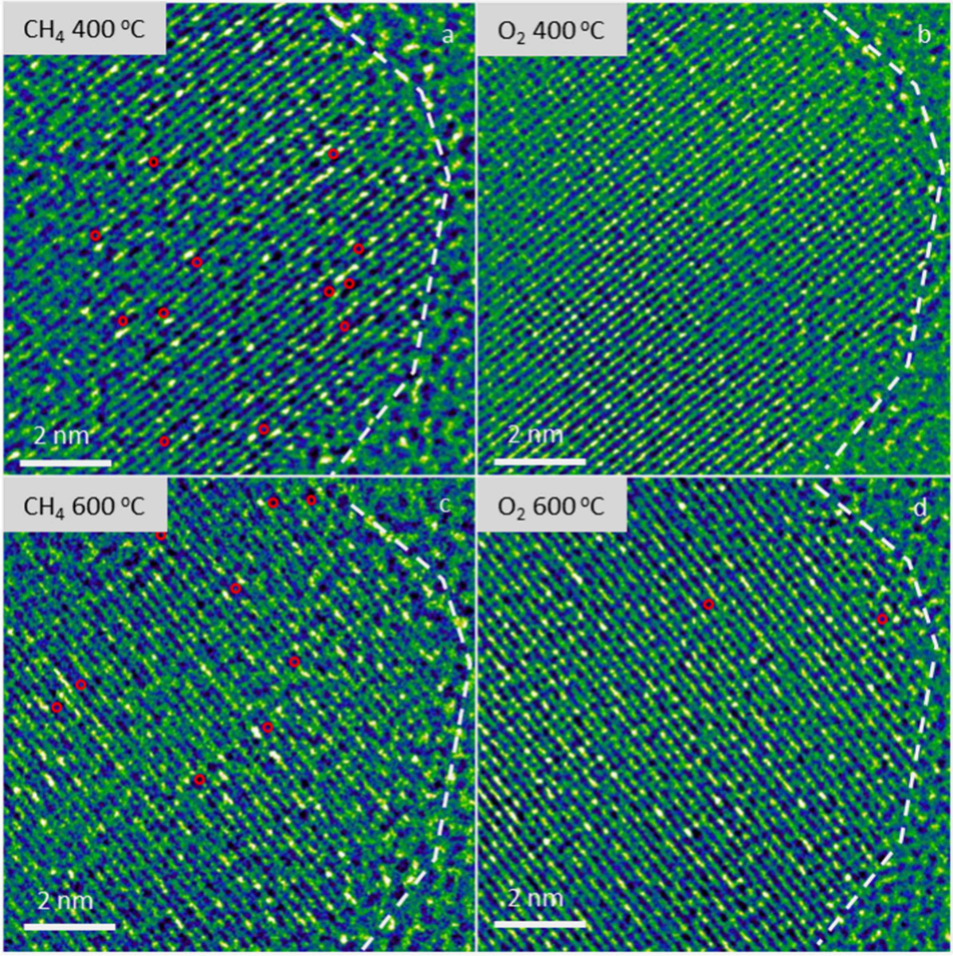
Fake-color high resolution *in situ* ETEM image of SA-La/MgO under alternating gas environment at elevated temperature: in CH_4_ at 400 °C for 616 s (a); switched to O_2_ and held at 400 °C for 1332 s (b); temperature increased to 600 °C and switched back to CH_4_, held for 570 s (c); switch to O_2_ and held at 600 °C for 416 s (d). All observations were conducted in the same sample area. Red circles mark the position of “dim sites”.

*In situ* EELS spectra also confirm the activity of lattice O^2–^_act._ derived from the La–O–Mg “slingshot” structure in the OCM reaction. Figure 5c presents the *in situ* EELS spectrum of the O–K ELENS of SA-La/MgO under different reaction conditions. The prepeak detected on fresh SA-La/MgO, positioned at 532.4 eV, originates from the interaction between the O 2p orbital and the d band of the transition metal,^30^ indicating the presence of La–O–Mg sites on the sample. In contrast, no prepeak at 532.4 eV was detected on intrinsic MgO. When SA-La/MgO undergoes reaction in a methane atmosphere at 400 °C, as depicted by the blue line in Figure 5c, the prepeak at 532.4 eV disappears, suggesting the consumption of lattice O^2–^_act_ from La–O–Mg. A similar result was observed when SA-La/MgO was exposed to methane at 600 °C (Figure S19a). Correspondingly, the *in situ* DRIFTs spectra in Figure 5a,b demonstrate that the dominant surface CH_*x*_ species are detected at wavenumbers of 1430–1410 cm^–1^ after methane activation on SA-La/MgO at 400 °C, whereas these species were detected on PA-La/MgO and MgO at the higher temperature of 600 °C.

**Figure 5:**
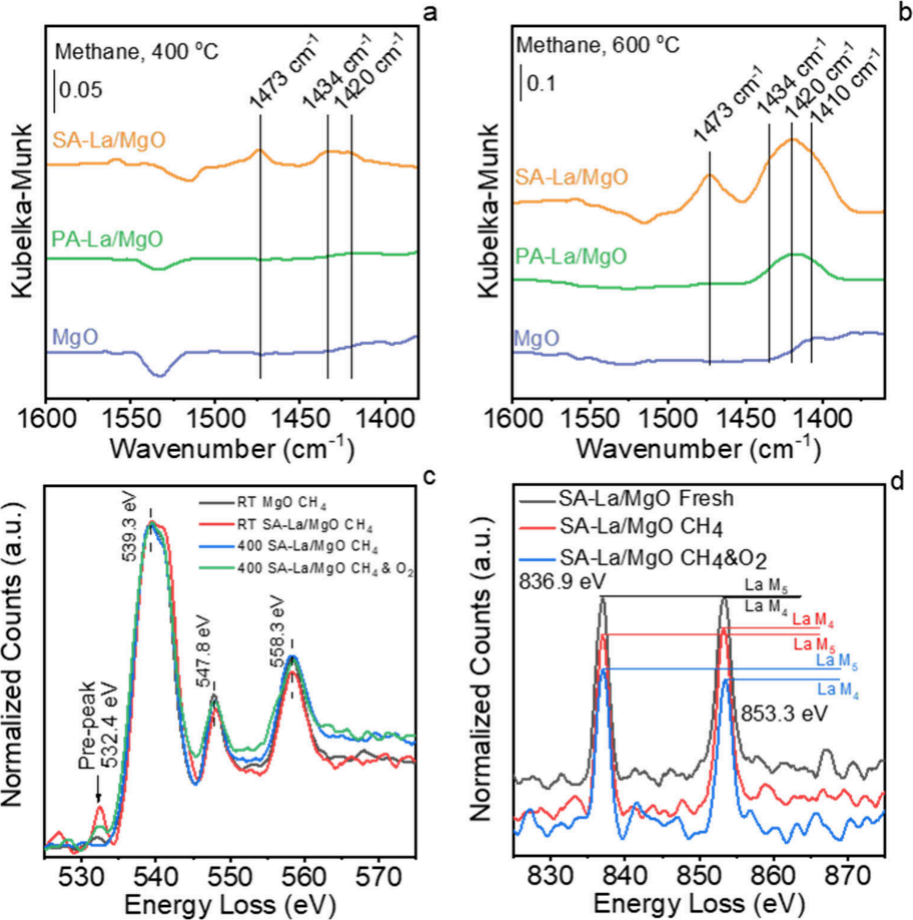
*In situ* DRIFTS spectra of PA-La/MgO, SA-La/MgO, and intrinsic MgO exposed in methane at 400 °C (a) and 600 °C (b); O–K EELS spectra of MgO and SA-La/MgO under different conditions (c); and *in situ* EELS La M_4,5_ spectra of fresh SA-La/MgO and SA-La/MgO being exposed to CH_4_ or CH_4_ and O_2_ (d).

During the methane activation process on SA-La/MgO (without the introduction of O_2_), lattice O_2_^–^ species, which play an active role and stem from the La–O–Mg slingshot structure, are consumed. Therefore, the replenishment of these consumed lattice O_2_^–^ species through the introduction of O_2_ is crucial to ensure continuous progress of the reaction. Figure 6a presents the DIRFTS spectra of SA-La/MgO after being pretreated in pure methane for 30 min and then exposed to an oxygen environment. The DRIFTS peak centered around 1175 cm^–1^ corresponds to the O_2_^–^ species,^31^ indicating that the dioxygen molecule can be adsorbed onto the oxygen vacancies created by methane activation.^32^ As a comparison, if the SA-La/MgO was not pretreated in methane, no O_2_^–^ can be detected on the sample surface, suggesting that oxygen vacancies are essential for the adsorption and activation of O_2_. The EELS spectra presented in Figure 5c and Figure S19b demonstrate that the O–K prepeak reappears when SA-La/MgO is exposed to CH_4_ and O_2_ at 400 and 600 °C. This indicates that the lattice O_2_^–^ species in the La–O–Mg slingshot structure can be replenished after the introduction of reactant O_2_.

**Figure 6:**
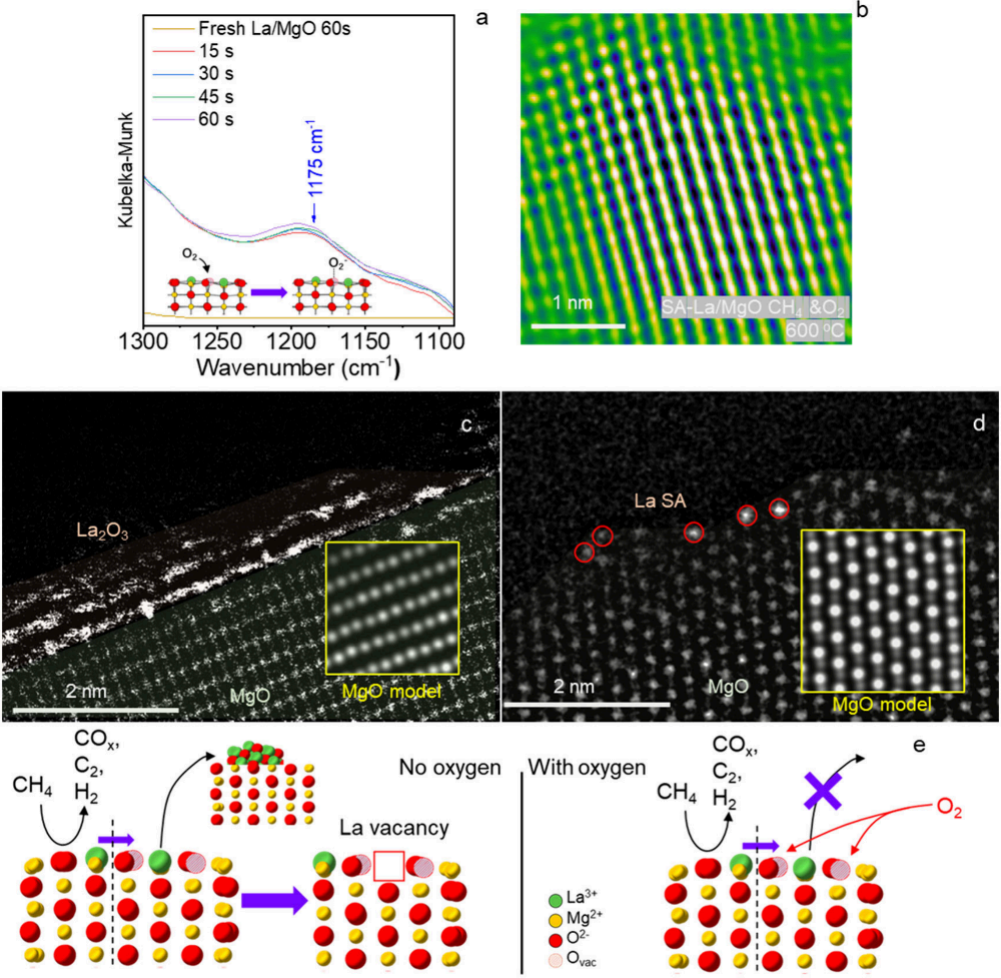
*In situ* DRIFTS spectra for fresh and postused SA-La/MgO heated in oxygen for 60 s (a); *in situ* ETEM image for SA-La/MgO reacted in the mixture of methane and oxygen at 600 °C (b); *ex situ* HAADF-STEM image of SA-La/MgO postused in methane (c) or in methane and oxygen (d); and the illustration of the movement of La in La–O–Mg sites under different reaction conditions (e).

Apart from spectroscopy, the *in situ* ETEM images are acquired to demonstrate the change in surface vacancies on SA-La/MgO. The *in situ* ETEM image of SA-La/MgO (Figure 6b) when exposed to methane and oxygen at 600 °C reveals that no obvious oxygen vacancies were detected. This indicates that the oxygen vacancies continuously generated from the methane-surface reaction have been replenished by continuously adsorbed oxygen. This phenomenon is also corroborated by ETEM images taken at other temperatures (Figures S21–S23). Furthermore, the R-space K edge extended electron energy loss fine structure spectrum for SA-LaMgO before and after exposure to CH_4_ (Figure S24) determines the O–La coordination declination after SA-LaMgO heated in a CH_4_ environment, suggesting the O^2–^_act._ in La–O–Mg was reacted with CH_4_. Taking all these results into consideration, the OCM reaction on SA-La/MgO can be summarized as follows. The La–O–Mg in MgO activates the lattice oxygen by modifying the geometric and electronic structures^27^ The methane reaction is initiated on the highly active lattice O^2–^_act._ of La–O–Mg, generating oxygen vacancies. Subsequently, dioxygen molecules accept electrons and are chemisorbed at this site, transforming into O_2_^–^. Finally, the chemisorbed O_2_^–^ continuously accepts electrons and is converted into a new surface lattice O^2–^_act._ of La–O–Mg, thus closing the cycle.^32^ The mechanism is depicted in Scheme 1.

**Scheme 1:**
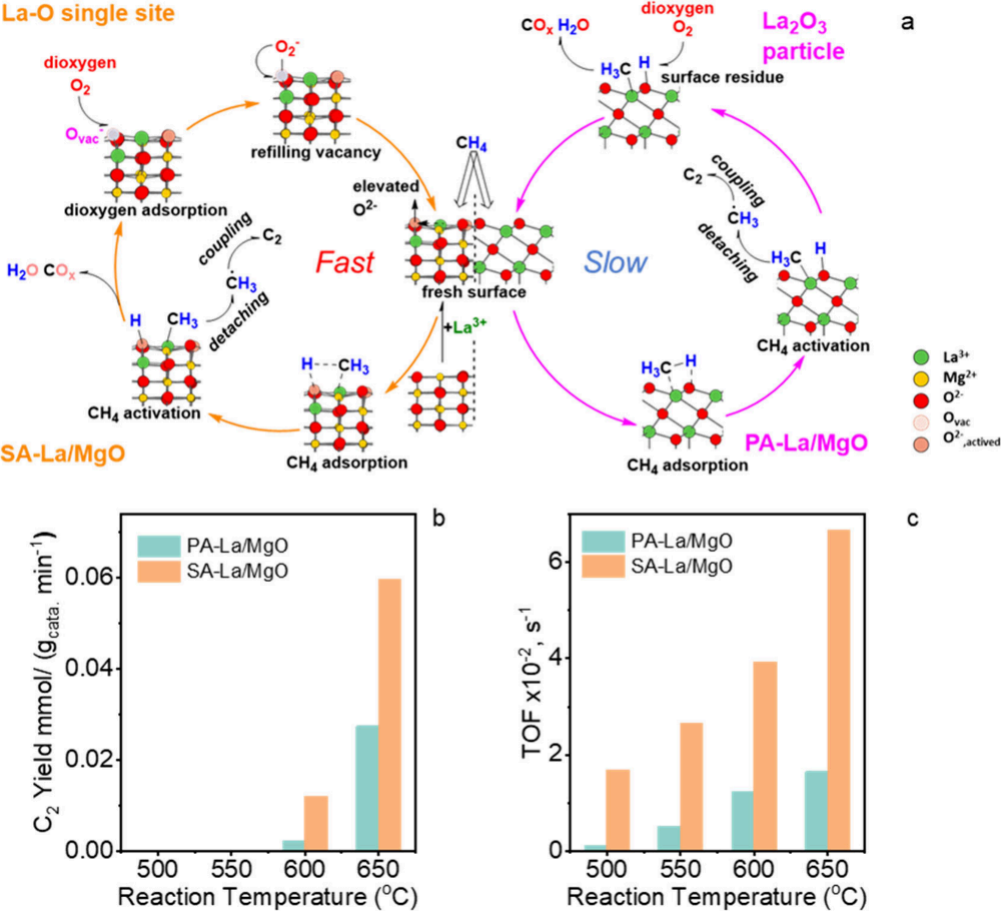
Proposed OCM over SA-La/MgO and PA-La/MgO^a^

a Green balls represent La, red balls represent O, pink balls represent activated surface lattice O^2–^_act._ in La–O–Mg slingshot site, and yellow balls represent Mg (a). C_2_ yield of PA-La/MgO (b) and the apparent TOF of PA-La/MgO and SA-La/MgO (c).

In addition to facilitating the reaction progression, lattice O^2–^_act_ in La–O–Mg also serves to stabilize the isolated La atom. We analyzed the spent SA-La/MgO catalyst after it had reacted either with methane alone or with a mixture of methane and oxygen through HAADF-STEM images (Figure 6c,d). In the HAADF-STEM image of the SA-La/MgO used in a methane-only atmosphere, the aggregation of lanthanum was observed. Moreover, the HRTEM image (Figure S25) revealed holes on the catalyst (marked with a red square). The lattice spacings in Figure S25a, which are 0.15 and 0.21 nm, correspond to the (110) and (100) facets of MgO. This suggests that the holes are formed on a MgO-based material (i.e., the La–O–Mg sites on SA-La/MgO). These images confirm that the isolated La atoms on SA-La/MgO will aggregate if the reaction occurs in a methane-only atmosphere. As more and more lattice O^2–^_act._ in La–O–Mg is consumed, the isolated La atoms lose their anchoring and aggregate to a more stable state. The lanthanum M_4,5_ ELENS of the fresh SA-La/MgO and the SA-La/MgO after exposure to methane (Figure 5d and Figure S20) indicated that some La elements in SA-La/MgO aggregated to form La_2_O_3_ after reaction with methane. On the contrary, if the reaction takes place in a mixture of methane and oxygen, the HAADF-STEM image (Figure 6d) shows well-dispersed La single sites on the spent catalyst, and the HRTEM image (Figure S25b) indicates that no holes are observed on the catalyst surface. The corresponding EELS spectra (Figure 5d) show that La_2_O_3_ was not generated, indicating that the La–O–Mg site is stable under these reaction conditions. The comparison of these two spent catalysts demonstrates that the active surface lattice oxygen adjacent to the SA La not only acts as the active site for methane activation but also stabilizes the La–O–Mg structure during the reaction.

### 3.2 No O Defect Generated at Reaction Conditions on PA-La/MgO

Regarding the methane conversion over PA-La/MgO, the generation of oxygen vacancies on intrinsic La_2_O_3_ nanoparticles is challenging. This is because the generation energy of oxygen vacancies on La_2_O_3_ is extremely high.^33^ We conducted a further in-depth investigation of methane activation over PA-La/MgO. As presented in Figure S17a, at room temperature, the La_2_O_3_ particle on PA-La/MgO exhibited an oriented structure. The lattice distance marked in Figure S17a is 0.35 nm, which corresponds to the (100) facet. When the La_2_O_3_ nanoparticle was exposed to methane at 400 or 600 °C, it maintained the oriented structure, and no obvious defects were observed (Figure S17b,c). The *in situ* La M_4_,_5_ EELS spectra of PA-La/MgO, as shown in Figure S18, indicate a stable La chemical environment, even when the sample is heated in methane up to 600 °C. *In situ* DRIFTS analysis of PA-La/MgO exposed to CH_4_ at 600 °C detected the adsorption of surface hydrocarbon fragments, which confirms that surface methane activation indeed occurred on PA-La/MgO. During the CH_4_ activation process, protons are attracted by lattice O^2–^ on the surface of the La_2_O_3_ nanoparticle. These surface protons (H^+^) react with O_2_ to form water, which is then removed from the surface of Pa–La/MgO.^34^ As a result, the fresh surface of La_2_O_3_ can be re-exposed, thereby continuously driving the reaction. The mechanism is illustrated in Scheme 1a. With this unique active site geometry over SA-La/MgO, the CH_4_ conversion and C_2_ selectivity (Figure S26) was improved. As displayed on Scheme 1b,c, the C_2_ yield over SA-La/MgO (5.96 × 10^–2^ mmol/(g_cata._ min)) is more than two-folded compared to PA-La/MgO (2.75 × 10^–2^ mmol/(g_cata._ min)), and the apparent TOF of methane conversion over SA-La/MgO also significantly improved, which is around 4 times higher (6.39 × 10^–2^ vs 1.65 × 10^–3^ @ 650 °C) than the over PA-La/MgO, indicating the excellent activity and C_2_ selectivity of this active site.

## 4 Conclusion

Herein, through the integration of various structural and surface characterization techniques, we confirm that the catalyst geometry is yet another crucial factor in enhancing the OCM performance. The SA La within MgO gives rise to the La–O–Mg slingshot geometry, which covers the surface of SA-La/MgO. This geometry plays a significant role in activating the surface lattice of the atoms, O_2_^–^ (designated as O^2–^_act._) in the vicinity of the La single atom, thereby enhancing the reaction efficiency. Simultaneously, these active surface lattice O^2–^_act._ species originating from the La–O–Mg sites are consumed during methane conversion, leading to the formation of oxygen vacancies. Consequently, they require replenishment by the introducing dioxygen reactants. This not only sustains the progression of the reaction but also ensures the structural stability of the single La atoms, preventing their aggregation. Conversely, in the case of the reaction occurring over the La_2_O_3_ nanoparticle geometry on PA-La/MgO, no generation of this surface-active oxygen species for the reaction is observed, resulting in poor OCM performance. This result holds significant importance for further research in the field of the OCM and suggests the potential for designing highly efficient catalysts for challenging reactions.

## Supplementary Material





## Notes

Table of selected OCM performance data reported over similar system; methane activation mechanism scheme; magnified XRD results; supplementary XPS results; low-magnification HAADF-STEM images; STEM EDS mapping of samples; apparent activation energy calculation; supplementary ETEM images of samples under different environments; supplementary *in situ* EELS results of samples under different conditions; supplementary HRTEM images; supplementary EXELFS spectrum; supplementary OCM performance results (PDF)
Transparent Peer Review report available (PDF)
