# Supplementary material for: Active Slingshot Geometry Site on Single-Atom La Catalyst Largely Promotes Oxidative Methane Coupling
Source: ACS Cent Sci. 2025 Sep 20;11(11):2188–95. doi: 10.1021/acscentsci.5c01016 (PMC12670278; doi:10.1021/acscentsci.5c01016)
Supplement: Supplementary file 1 [file oc5c01016_si_001.pdf]

Supporting information of "Active Slingshot Geometry Site on Single-Atom La Catalyst Largely Promotes Oxidative Methane Coupling"

Lizhuo Wang[1], Liwei Cao[2], Ang Li[2,*], Wenjie Yang[1], Wei Li[2], Xiaozhou Liao[3], Xiaodong Han[2,4*], Jun Huang[1,*]

[1] Laboratory for Catalysis Engineering, School of Chemical and Biomolecular Engineering, Sydney Nano Institute, The University of Sydney, NSW, 2006, Australia

[2] Beijing Key Laboratory of Microstructure and Property of Advanced Materials, Beijing University of Technology, Beijing, 100124, China

[3] Australian Centre for Microscopy & Microanalysis and School of Aerospace, Mechanical and mechatronic Engineering, The University of Sydney, Sydney, NSW, 2006, Australia

[4] Department of Materials Science and Engineering, Southern University of Science and Technology, Shenzhen, 518055, China

[*] corresponding authors: ang.li@bjut.edu.cn; hanxd@sustech.edu.cn; jun.huang@sydney.edu.au

Table S1 Selected OCM performance data reported for La-promoted MgO and intrinsic La_2O_3.

Entry	Catalyst	La/Mg molar ratio	Reaction temperature (^oC)	Molar ratio CH_4/O_2	Space Velocity (ml/g/hr)	CH4 conversion (%)	C2 selectivity (%)	References
1	La-MgO	0.1	800	3.2	18,000	27.8	40.5	[1]
2	La-MgO	0.1	800	4	102,000	24.7	63.5	[2]
3	La-MgO	0.005	700	4	51,600	25.8	46.6	[3]
			800	4	51,600	30.9	59.6	
4	La/MgO	0.11	700	4	51,600	24.8	34.1	[3]
			800	4	51,600	29.5	53.5	
5	La-MgO	0.02	650	4	51,360	1.9	62.6	[4]
6	La2O3	N/A	800	4	102,000	25.7	57.6	[2]
7	La2O3	N/A	800	3	12,000	28.4	36.7	[5]
8	La2O3	N/A	600	3	60,000	14.7	23.7	[6]

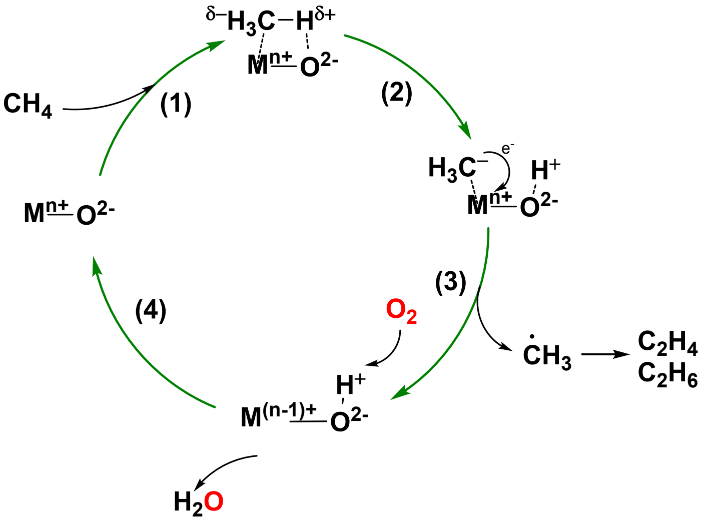

Scheme S1 Methane activation mechanism based on acidity/basicity theory

XRD:

The XRD results are shown on Figure 1 d. The result for MgO shown its intrinsic peak at 36.9 °, 42.9 °, 62.3 °, 74.7 ° as well as 78.6 ° (according to synthesized periclase MgO, PDF#45-0946). After the introducing of lanthanum particle, a couple of new peaks emerged at 15.6 °, 27.3 °, 28.0 °, 39.5 ° and 48.7 °. These newly generate peaks are attributed to the $La(OH)_3$ (PDF#36-1481) which may come from the reaction between surface La_2O_3 particles and the moisture in the air. The similar phenomenon is also observed on the synthesized La_2O_3 material. The La_2O_3 particle sizes over PA-La/MgO is calculated via Scherrer equation[7]. It is determined the La_2O_3 particle size over PA-La/MgO is around 18.3 nm. For SA-La/MgO, the peaks for La_2O_3 or $La(OH)_3$ are not observed, indicating the La^{3+} is in the form of single sites. On both PA-La/MgO and SA-La/MgO, the intrinsic peak for MgO remains its pattern. No peak position shift is observed over the XRD pattern, indicating the crystal structure of MgO is not dramatically influenced by the introducing of lanthanum.

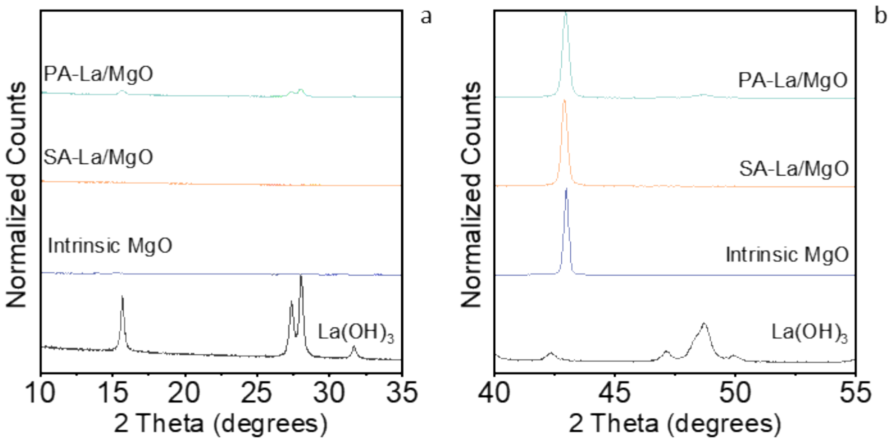

Figure S1 XRD patterns of PA-La/MgO, SA-La/MgO, Intrinsic MgO, and $La(OH)_3$ in the region between 10° to 35° (a) and 40° to 55° (b).

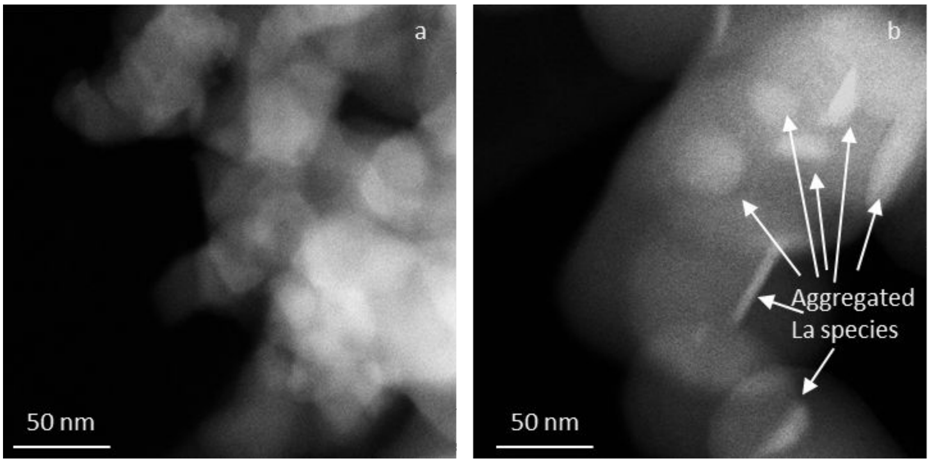

Figure S2 The low-magnification HAADF-STEM image of SA-LaMgO (a) and PA-LaMgO (b)

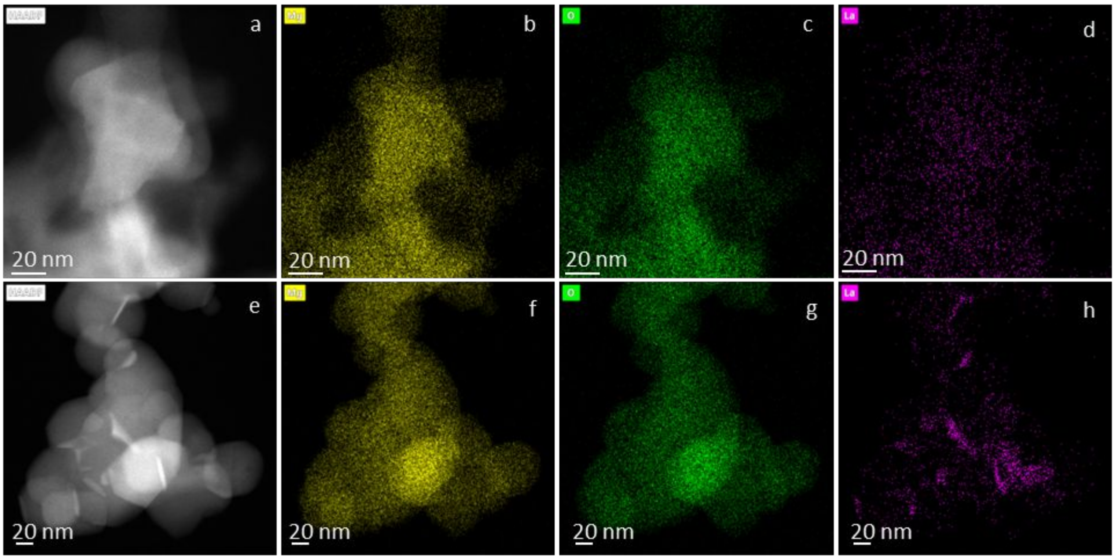

Figure S3 The HAADF-STEM image (a) and corresponding Mg (b), O (c), and La (d) EDS mapping

for SA-LaMgO; The HAADF-STEM image (e), and corresponding Mg (f), O (g), and La (h) EDS mapping for PA-LaMgO

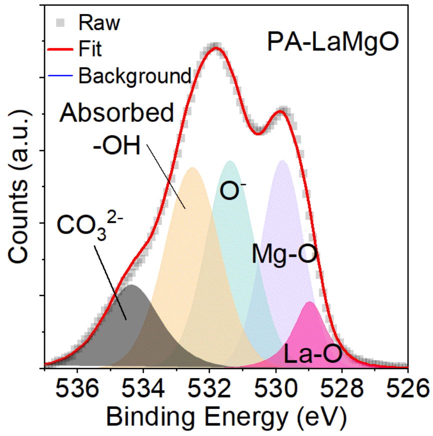

Figure S4 O 1s XPS result of PA-La/MgO

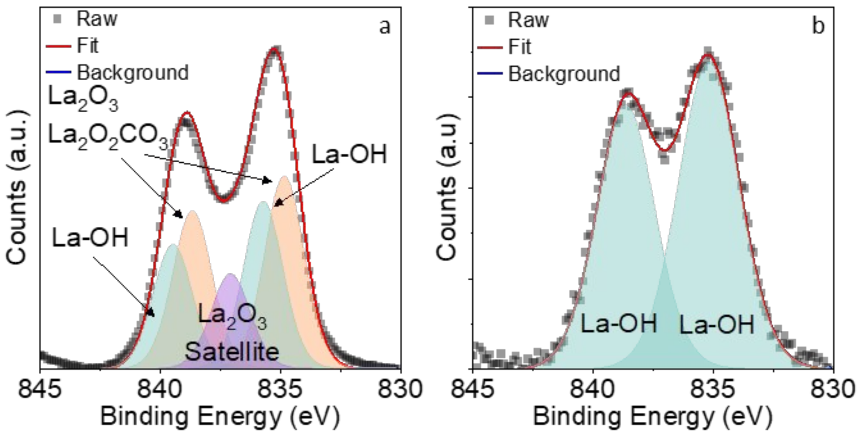

Figure S5 La3d XPS results of PA-La/MgO (a) and SA-La/MgO (b)

From the XPS results shown on Figure 1 (e) (f) and S5 (a) (b). The O1s spectrum of pure MgO can be deconvoluted into three peaks, listed as lattice O (Mg-O) at 530.0 eV[8], peroxide (O^{-1}) at 531.6 eV [8] and absorbed -OH at 532.5 eV[9]. After the lanthanum singe stie is introduced into the catalyst, the aforementioned peak position remains steady. Interestingly, the fraction of the surface -OH group decreased, and a new peak attributed to La-O which located at 528.9 eV is observed[10]. The carbonate group, which may come from CO_2 contamination, located at 534.4 eV was observed in O 1s XPS spectra for PA-LaMgO (Figure S4).[11] This result is corresponding to the published results.[12] As for the La 3d spectrum, the PA-La/MgO is applied as a reference and the result is shown on Figure S5 (a). The binding energy located between

c.a. 830~840 eV is La3d 5/2 orbital [13]. Further analysis of the results indicates that two species are found in the sample. The peaks located at around 834.6 eV and 839.0 eV are attributed to the mixture of La_2O_3 and $La_2O_2(CO)_3$.[14] Apart from La_2O_3, the La-OH generated from La_2O_3 and moisture is another important component in the sample. The peaks for $La(OH)_3$ are located at around 835.2 eV and 838.8 eV [14]. It is indicated more surface $La(OH)_3$ species are observed on La 3d XPS spectra over SA-La/MgO compared to La_2O_3. It is corresponding to the XRD results that some lanthanum component on La/MgO are transferred to $La(OH)_3$ due to absorbing the vapor in the air. As for the SA-LaMgO (Figure S5b), the only observed La species is La-OH. The absence of La_2O_3 and $La_2O_2(CO)_3$ suggests there was no La_2O_3 on SA-LaMgO surface, suggesting the well dispersion of La on SA-LaMgO.

CrystalMaker simulation:

The simulation was carried out in CrystalMaker® 11 software. The MgO crystal structure was obtained from ICSD database (ICSD#9863). The MgO size was 2x2x2 cells and the structure was transferred from crystal to molecule before substitute one surface Mg cation by La cation. Then the whole structure underwent relaxation until the stable energy level of the crystal structure was reached.

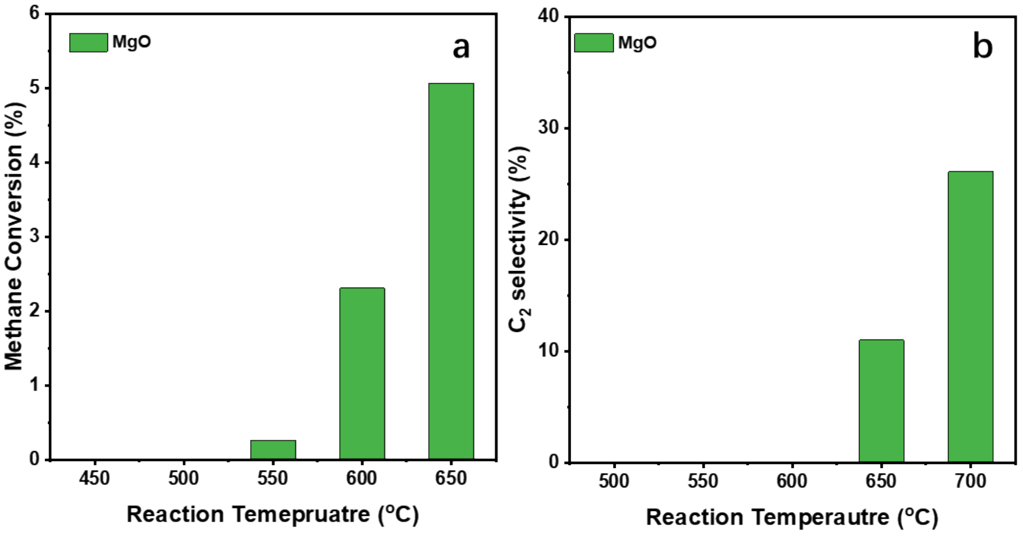

Figure S6 Methane conversion over intrinsic MgO at different temperature (a); C_2 selectivity of the reaction over intrinsic MgO at different temperature (b)

To verify the performance of La/MgO in the OCM reaction, the product from DRIFTS was analysed by GC. As displayed over Figure 1g, the observed methane conversion over SA-La/MgO and PA-La/MgO is recorded as 1.57% and 0.27 % respectively when temperature as low as 450 ^{o}C while the methane conversion over intrinsic MgO can only be observed when the temperature is higher than 550 ^{o}C (Figure S6). Around 3.21 % of inlet methane is converted at 550 ^{o}C over SA-La/MgO and the one on PA-La/MgO was only 1.42 % at that temperature. Although the methane conversion is increased along with temperature for both La/MgO and intrinsic MgO, the difference of methane conversion over La/MgO or intrinsic MgO is enlarged as well. More than 4.75 % of methane is converted over SA-La/MgO at 600 ^{o}C and less than 2.30 % methane can be converted under same condition over intrinsic MgO. The methane conversions over PA-La/MgO and SA-La/MgO are 5.25% and 8.05% when temperature increased to 650 ^{o}C, while the methane conversion at same temperature over intrinsic MgO is only 5.07%. Compared to intrinsic MgO, both of the La-doped MgO has lower start-off temperature, which suggested the possibility that different methane activation sites are existing over Lg/MgO and intrinsic MgO.

As for the yield of ethane and ethylene products displayed on Scheme 1 b, at a lower temperature (<500 ^{o}C), the carbon oxides are the only products since the generated methyl radical needs energy to detached from the surface of the catalyst [15]. The C_2 species over both SA-La/MgO and PA-La/MgO are observed when the temperature reached 600 ^{o}C. The intrinsic MgO needs an even higher temperature, which is 650 ^{o}C, to generate ethane and ethylene. When the temperature reached 650 ^{o}C, the intrinsic MgO displayed a 2.3 % methane conversion and 12% C_2 selectivity and these number increased to 5.0% and 26% at 700 ^{o}C. Since the La/MgO displayed a superior conversion rate of methane and an excellent selectivity to C_2 species, it took the advantage for C_2 species yield. The sum of ethane and ethylene yield over SA-La/MgO achieved 0.075 mmol C_2/ ($g_{cata} \cdot$ min) at 650 ^{o}C and 0.146 mmol C_2/ ($g_{cata} \cdot$ min) for 700 ^{o}C. The C_2 yield over PA-MgO was only 0.051 mmol C_2/($g_{cata} \cdot$ min) at 650 ^{o}C and 0.053 mmol C_2/($g_{cata} \cdot$ min) at 700 ^{o}C. It can be evident that, the C_2 yield over SA-

La/MgO at these temperatures are much higher than the one over PA-La/MgO, suggesting the superior reaction performance of the SA-La/MgO. For the temperature higher than 700 ^{o}C, considering the temperature is excessively higher than the light-off region, the gas-phase OCM reaction would take part a significant role[4]. Thus, the catalyst surface behaviours are no longer the determining factor of this reaction at that temperature, and we would not discuss it in this work.

To further comparing the performance of the single La-O-Mg sites, the TOF of the catalyst at this reaction condition is calculated. For example, at 550 ^{o}C, 1.42 % of methane can be converted over PA-La/MgO and 3.21 % of methane reacted over SA-La/MgO, while only negligible (0.26%) methane are converted over intrinsic MgO. Thus, turn over frequency (TOF) on PA-La/MgO is

$$TOF_{PA-La/MgO} = \frac{reactant\ consumed}{mole\ of\ catalyst} \times (reaction\ time)^{-1}$$

$$= \frac{1.42\% \times 4\,ml\ \times (22.4\frac{L}{mol})^{-1} \times 1 \times 10^{-3}\frac{L}{ml}}{\frac{(5\,wt\%\ \times 0.05\,g)}{183\frac{g}{mol}}} \times (60s)^{-1}$$

$$= 3.0 \times 10^{-3}\,s^{-1}$$

Turn over frequency (TOF) on SA-La/MgO is

$$TOF_{SA-La/MgO} = \frac{reactant\ consumed}{mole\ of\ catalyst} \times (reaction\ time)^{-1}$$

$$= \frac{3.21\% \times 4\,ml\ \times (22.4\frac{L}{mol})^{-1} \times 1 \times 10^{-3}\frac{L}{ml}}{\frac{(1\,wt\%\ \times 0.05\,g)}{183\frac{g}{mol}}} \times (60s)^{-1}$$

$$= 3.5 \times 10^{-1}\,s^{-1}$$

This calculation results, which displayed on Scheme 1c, determines the TOF over SA-La/MgO is around 5 times higher than the one over PA-La/MgO. Indicating the single atom La on SA-La/MgO lead to an excellent performance of methane activation especially when the temperature is lower than the light-off temperature. The single atom La might introduced another type of active site for methane activation other than the one over La_2O_3 particles.

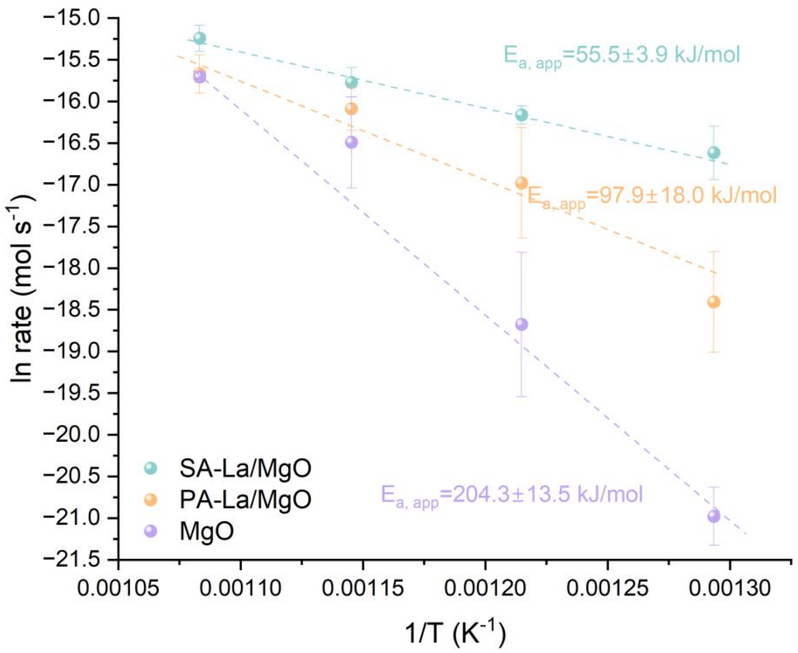

Figure S7 The apparent activation energy of methane conversion over SA-La/MgO, PA-La/MgO, and intrinsic MgO. The calculation was based on the reaction performance shown on Figure 1g and Figure S6.

Figure S7 gives the Arrhenius plots of the reaction rates with the corresponding reaction temperature on all three catalysts (namely, SA-La/MgO, PA-La/MgO and intrinsic MgO). The SA-La/MgO (light green dash) displayed the lowest apparent activation energy of methane conversion (55.5±3.9 kJ/mol), in contrast to that of 97.9±18.0 kJ/mol for PA-La/MgO (light orange dash). This number increased to 204.3±13.5 kJ/mol for intrinsic MgO (light purple dash). The lowest apparent activation energy over SA-La/MgO fully supports the notion that SA-La/MgO provides a highly active catalytic centre for methane activation and conversion. Additionally, the progressively increasing apparent activation energy over SA-La/MgO, PA-La/MgO, and intrinsic MgO indicates a corresponding increase in the difficulty of methane activation across these three materials. The apparent methane activation energy is comparable to the number over La_2O_3 nanoparticles[16-18], reflecting the methane may be activated on La_2O_3 nanoparticle on PA-La/MgO.

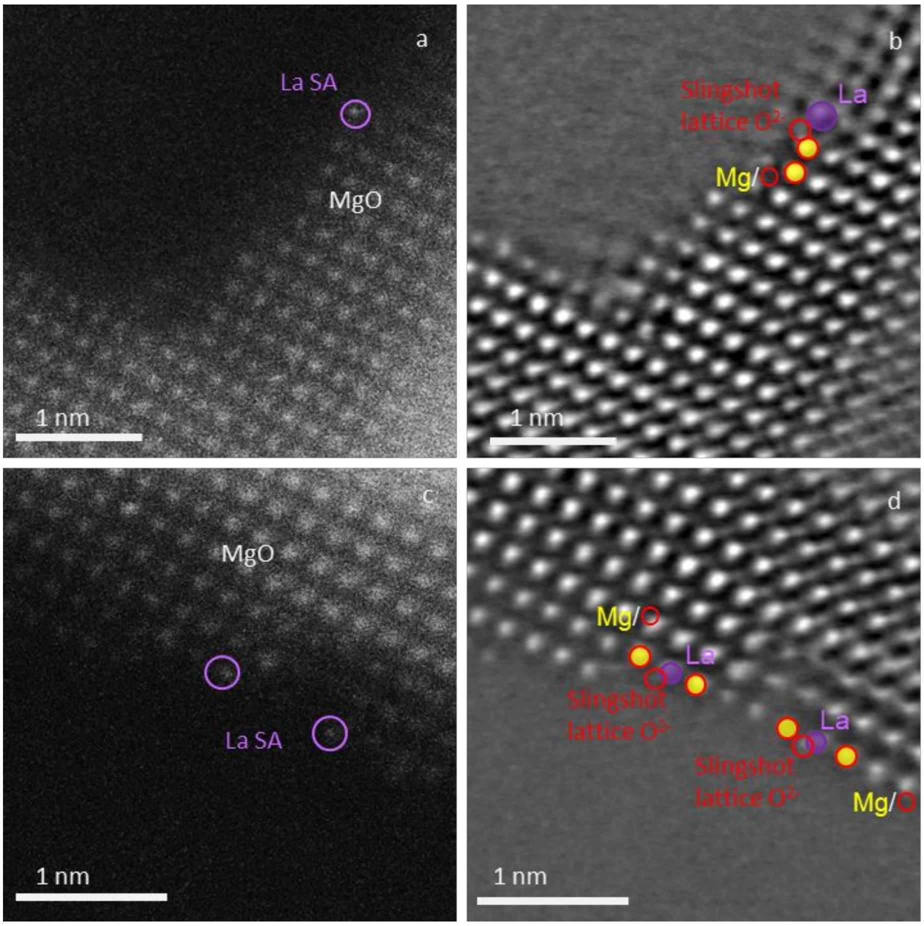

Figure S8 The HAADF-STEM image of SA-LaMgO (a) and the corresponding STEM-iDPC image of panel a (b); Another site HAADF-STEM image of SA-LaMgO (c) and the corresponding STEM-iDPC image of panel c (d). The STEM-iDPC image directly reveals the lattice O^{2-} in slingshot La-O-Mg geometry.

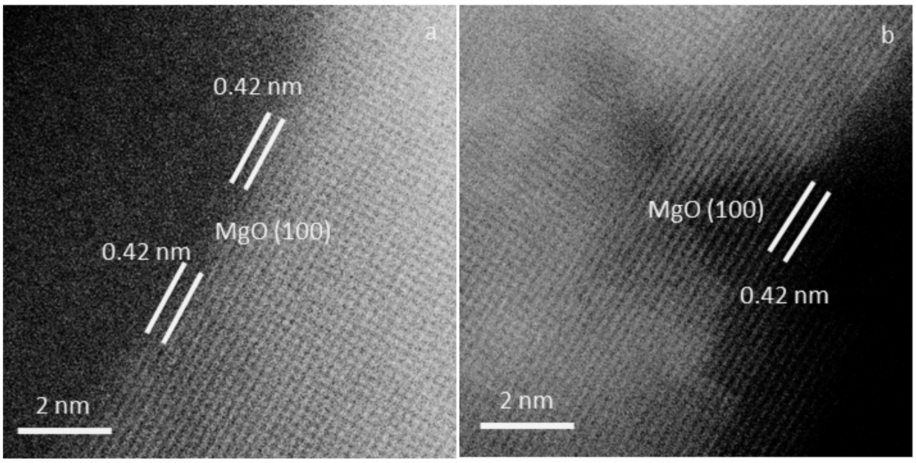

Figure S9 HAADF-STEM image of intrinsic MgO surface (a) and the HAADF-STEM image of intrinsic MgO surface at another site (b). "Slingshot structure" was not detected in the intrinsic MgO.

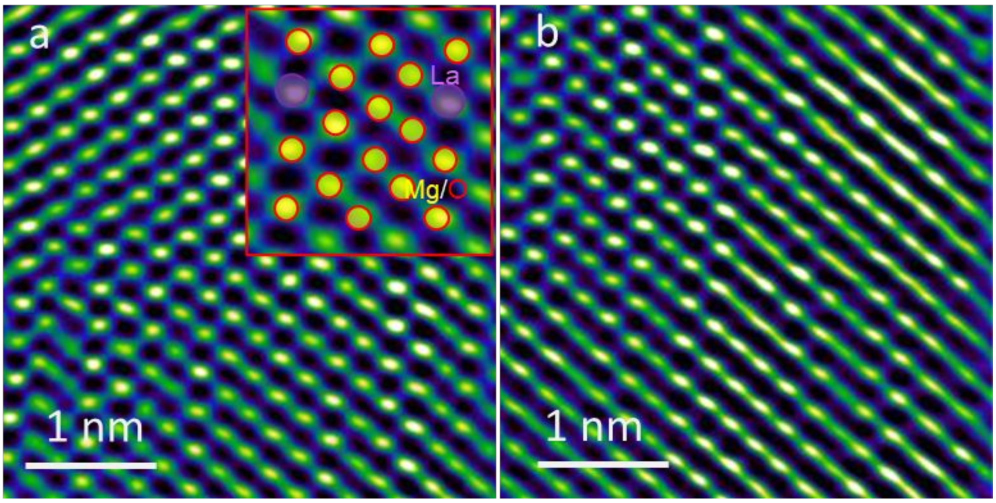

Figure S10 in-situ ETEM images of SA-La/MgO in methane atmosphere at 300 ^{o}C for 0 s (a) and 30 s (b)

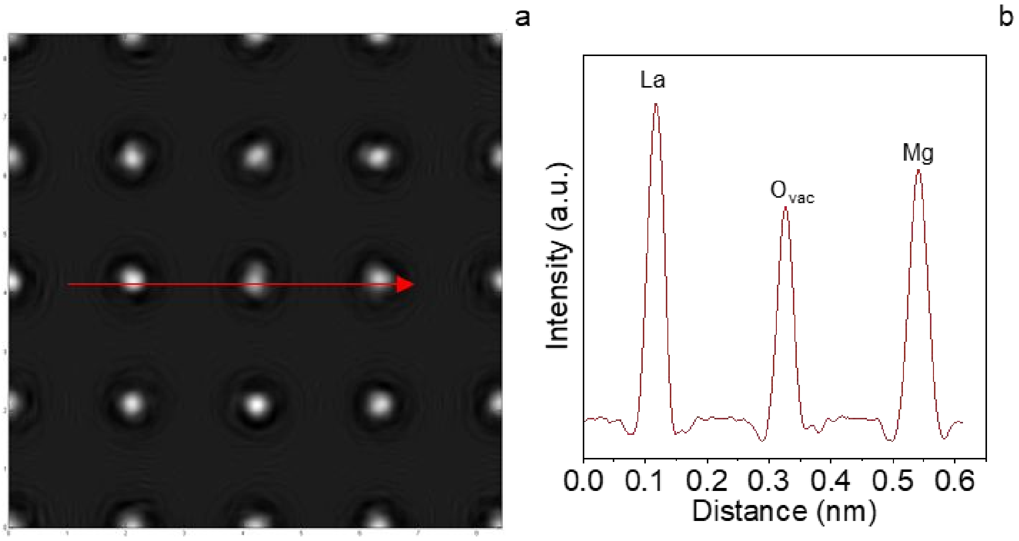

Figure S11 Simulated TEM of La-O_{vac}-Mg sites (a) and the contrast profile of the arrow region (b)

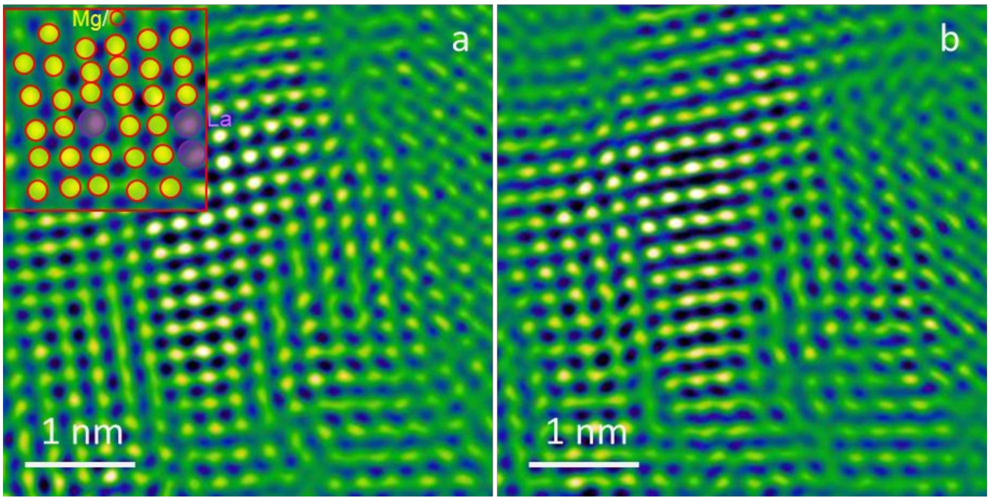

Figure S12 In-situ ETEM images of SA-La/MgO in oxygen atmosphere at room temperature for 0s (a) and 20s (b)

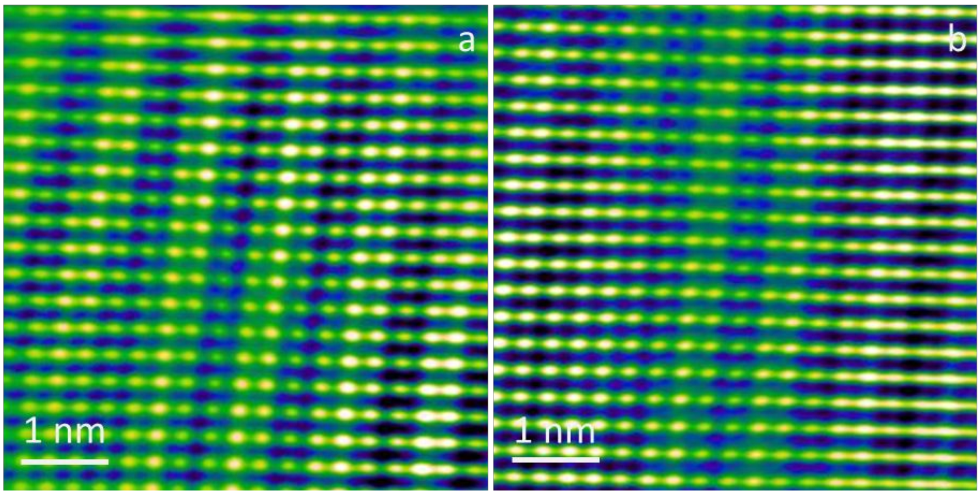

Figure S13 In-situ ETEM images of SA-La/MgO in oxygen atmosphere at 300 ^{o}C for 0s (a) and 75s (b)

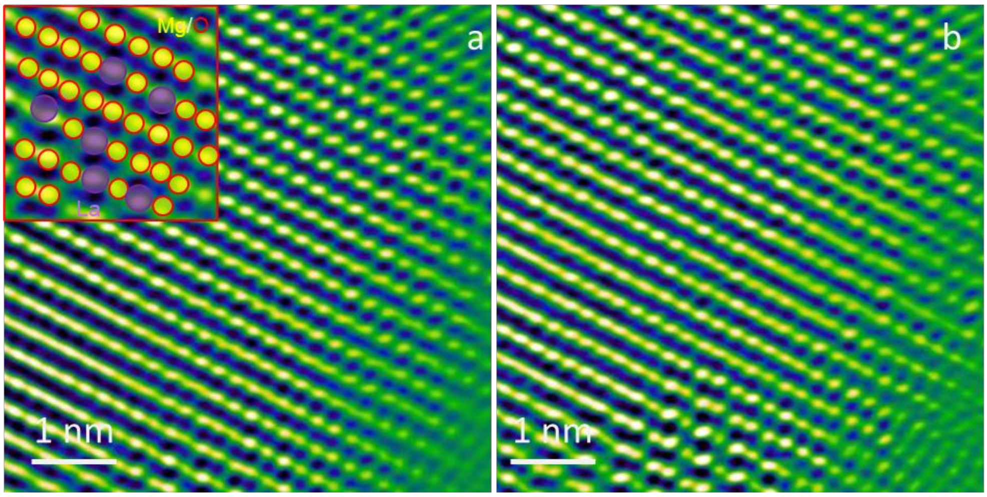

Figure S14 In-situ ETEM images of SA-La/MgO in oxygen atmosphere at 600 ^{o}C for 0s (a) and 20 s (b)

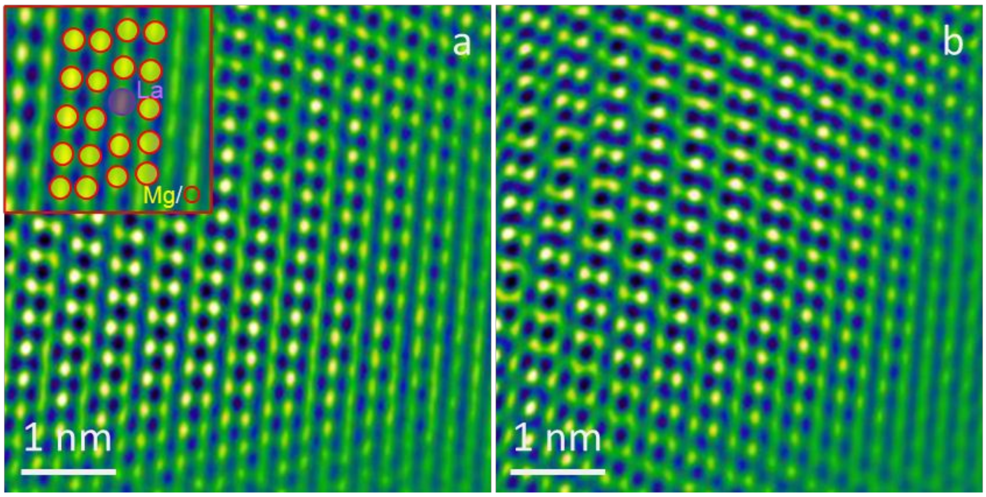

Figure S15 In-situ ETEM images of SA-La/MgO in oxygen atmosphere at 700 ^{o}C for 0s (a) and 40s (b)

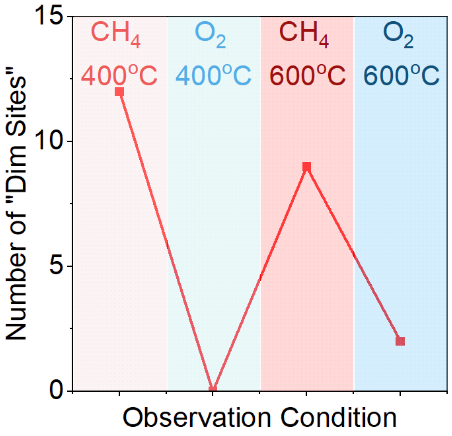

Figure S16 Profile of dim site density observed under different condition based on Figure 4. The observation was conducted at same area and the size is 10 nm x 10 nm

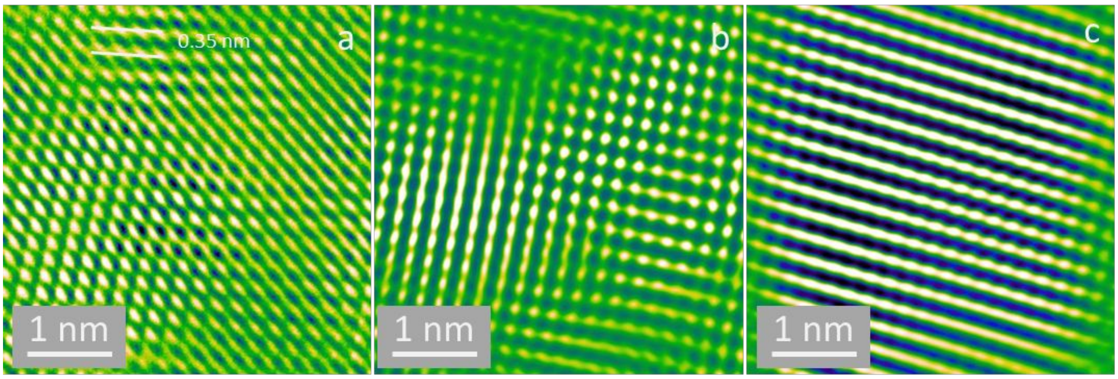

Figure S17 In-situ ETEM image of PA-La/MgO in methane atmosphere at room temperature (a); In-situ ETEM image of PA-La/MgO particle in methane atmosphere at 400 °C (b); In-situ ETEM image of PA-La/MgO particle in methane atmosphere at 600 °C (c) The pe-sudo colour is used for easier observation.

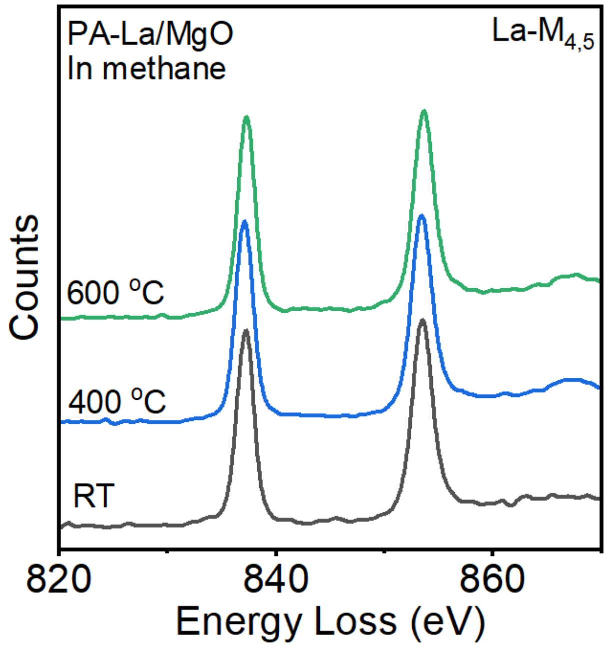

Figure S18 in situ La-$M_{4,5}$ EELS spectra of PA-La/MgO exposed in methane at different temperatures.

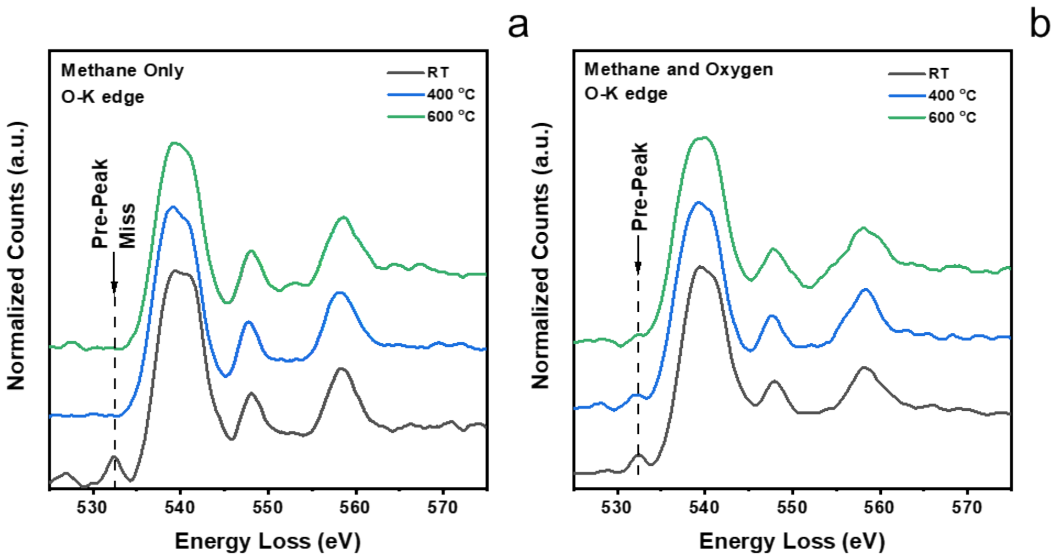

Figure S19 The O-K ELENS spectra of SA-La/MgO in methane atmosphere at different reaction temperature (a) and the O-K ELNES spectra of SA-La/MgO in methane and oxygen atmosphere at different reaction temperature (b). The spectra are acquired at the surface region of the catalyst.

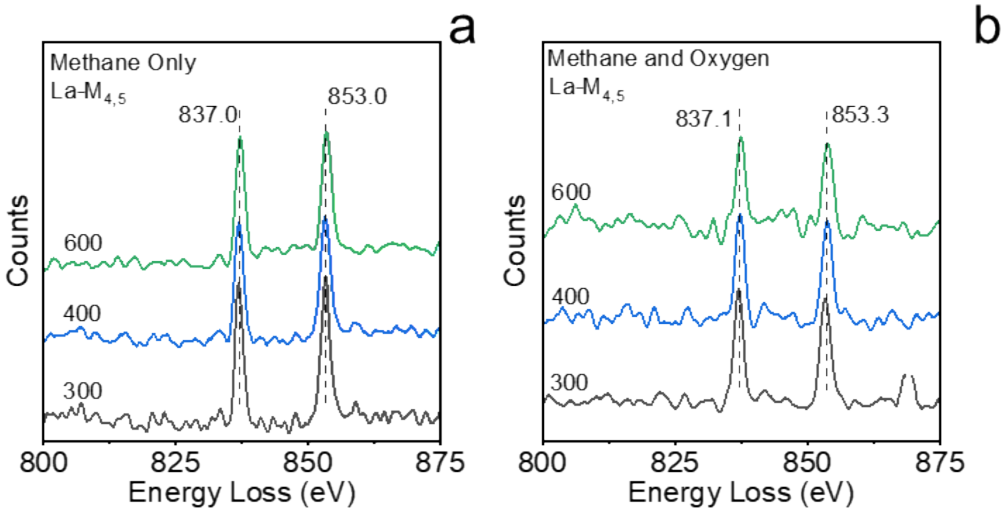

Figure S20 The La-$M_{4,5}$ ELENS spectra of SA-La/MgO in methane only atmosphere at different reaction temperature (a) and the La-$M_{4,5}$ ELENS spectra of SA-La/MgO in methane and oxygen atmosphere at different reaction temperature (b). The spectra are acquired at the surface region of the catalyst.

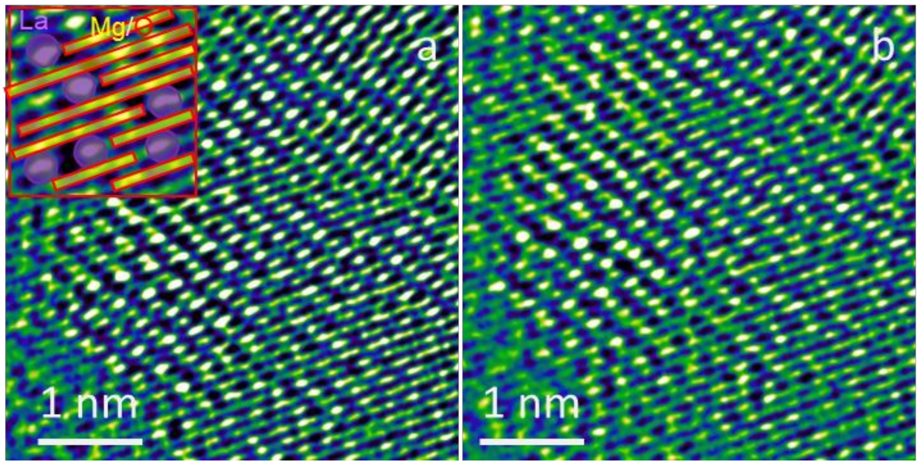

Figure S21 In-situ ETEM images of SA-La/MgO in methane and oxygen atmosphere at room temperature for 0s (a) and 5s (b)

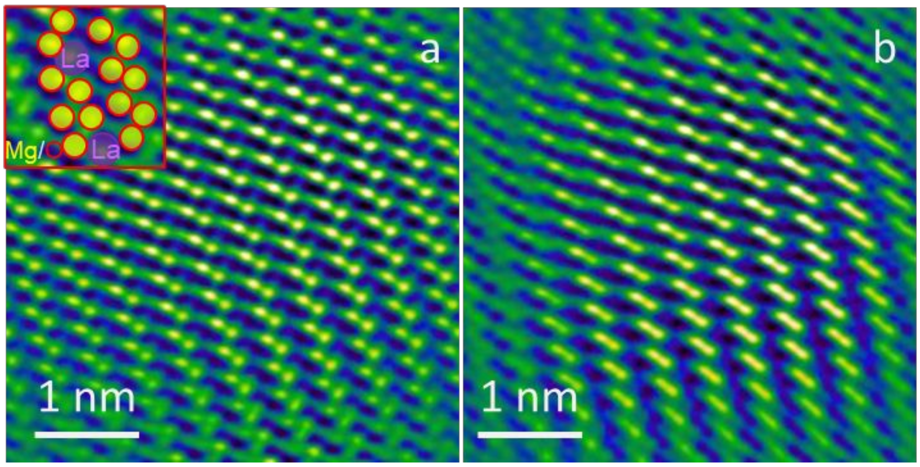

Figure S22 In-situ ETEM images of SA-La/MgO in methane and oxygen atmosphere at 300 ^{o}C for 0s (a) and 5s (b)

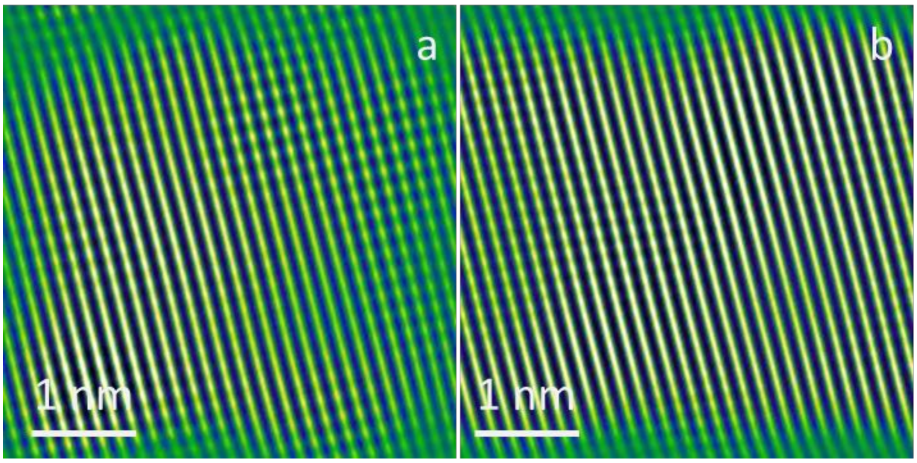

Figure S23 In-situ ETEM images of SA-La/MgO in methane and oxygen atmosphere at 400 ^{o}C for 0s (a) and 2s (b)

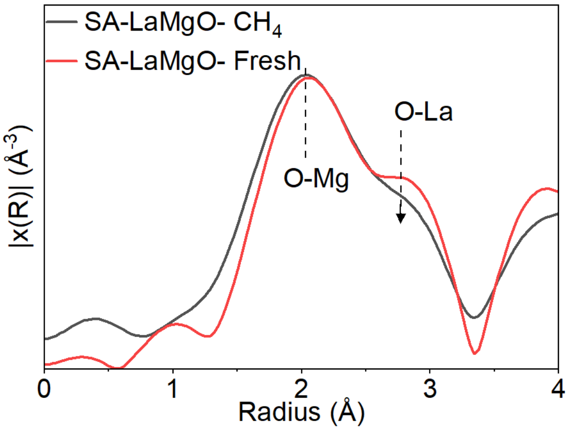

Figure S24 the extended electron energy loss fine structure (EXELFS) spectrum of SA-LaMgO before and after exposed to CH_4.

The R-space O1s EXELFS indicated two peaks in the region between 2-3 Å. The peak located at around 2.1 Å is attributed to O-Mg path,[19] whereas the peak located between 2.5 to 2.8 Å is attributed to O-La path.[20] This result determines that the reduction of La-O coordination after SA-La/MgO exposed in CH_4 environment.

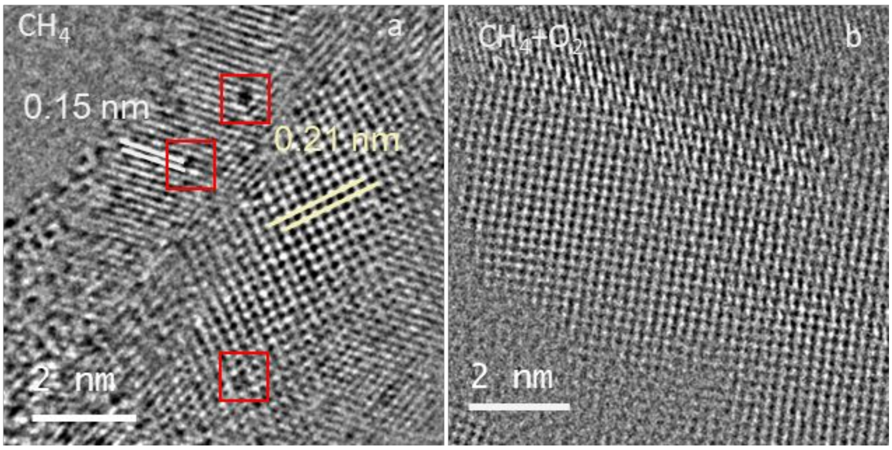

Figure S25 ex situ TEM image of SA-La/MgO post used in methane (a) and methane and oxygen (b)

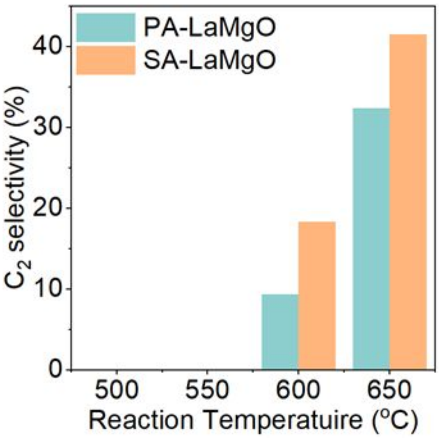

Figure S26 The C_2 selectivity over PA-LaMgO and SA-LaMgO

References

[1] V.R. Choudhary, V.H. Rane, S.T. Chaudhari, Pulse reactions of methane, ethane and ethylene over Li-, La- and Sm-promoted MgO catalysts in the presence and absence of free oxygen, React. Kinet. Catal. Lett., 63 (1998) 371-377.

[2] V.R. Choudhary, S.A.R. Mulla, B.S. Uphade, Oxidative Coupling of Methane over Supported La_2O_3 and La-Promoted MgO Catalysts: Influence of Catalyst−Support Interactions, Industrial & Engineering Chemistry Research, 36 (1997) 2096-2100.

[3] V.R. Choudhary, V.H. Rane, S.T. Chaudhari, Surface properties of rare earth promoted MgO catalysts and their catalytic activity/selectivity in oxidative coupling of methane, Applied Catalysis A: General, 158 (1997) 121-136.

[4] V.R. Choudhary, V.H. Rane, S.T. Chaudhari, Factors influencing activity/selectivity of La-promoted MgO catalyst prepared from La- and Mg- acetates for oxidative coupling of methane, Fuel, 79 (2000) 1487-1491.

[5] C. Guan, Y. Yang, Y. Pang, Z. Liu, S. Li, E.I. Vovk, X. Zhou, J.P.H. Li, J. Zhang, N. Yu, L. Long, J. Hao, A.P. van Bavel, How CO_2 poisons La_2O_3 in an OCM catalytic reaction: A study by in situ XRD-MS and DFT, J. Catal., 396 (2021) 202-214.

[6] Z. Liu, J.P. Ho Li, E. Vovk, Y. Zhu, S. Li, S. Wang, A.P. van Bavel, Y. Yang, Online Kinetics Study of Oxidative Coupling of Methane over La2O3 for Methane Activation: What Is Behind the Distinguished Light-off Temperatures?, ACS Catal., 8 (2018) 11761-11772.

[7] A. Monshi, M.R. Foroughi, M.R. Monshi, Modified Scherrer equation to estimate more accurately nano-crystallite size using XRD, World journal of nano science and engineering, 2 (2012) 154-160.

[8] Y. Wan, C. Samundsett, J. Bullock, M. Hettick, T. Allen, D. Yan, J. Peng, Y. Wu, J. Cui, A. Javey, A. Cuevas, Conductive and Stable Magnesium Oxide Electron-Selective Contacts for Efficient Silicon Solar Cells, Advanced Energy Materials, 7 (2017) 1601863.

[9] G. Suárez-Campos, D. Cabrera-German, J.A. García-Valenzuela, M. Cota-Leal, J.L. Fuentes-Ríos, M. Martínez-Gil, H. Hu, M. Sotelo-Lerma, Controlled synthesis of Mg(OH)2 thin films by chemical solution deposition and their thermal transformation to MgO thin films, Ceramics International, 45 (2019) 10356-10363.

[10] J. Song, C. Han, P.T. Lai, Comparative Study of Nb2O5, NbLaO, and La2O3 as Gate Dielectric of InGaZnO Thin-Film Transistor, IEEE Trans. Electron Devices, 63 (2016) 1928-1933.

[11] X. Chen, R. Ye, C. Jin, F. Hu, L. Zhou, Z.-H. Lu, R. Zhang, G. Feng, A highly efficient Ni/3DOM-$La_2O_2CO_3$ catalyst with ordered macroporous structure for CO_2 methanation, J. Catal., 428 (2023) 115129.

[12] C. Guan, Z. Liu, D. Wang, X. Zhou, Y. Pang, N. Yu, A.P. van Bavel, E. Vovk, Y. Yang, Exploring the formation of carbonates on La_2O_3 catalysts with OCM activity, Catalysis Science & Technology, 11 (2021) 6516-6528.

[13] S. Mickevičius, S. Grebinskij, V. Bondarenka, B. Vengalis, K. Šliužienė, B.A. Orlowski, V. Osinniy, W. Drube, Investigation of epitaxial LaNiO3−x thin films by high-energy XPS, J. Alloys Compd., 423 (2006) 107-111.

[14] J.P.H. Li, X. Zhou, Y. Pang, L. Zhu, E.I. Vovk, L. Cong, A.P. van Bavel, S. Li, Y. Yang, Understanding of binding energy calibration in XPS of lanthanum oxide by in situ treatment, Physical Chemistry Chemical Physics, 21 (2019) 22351-22358.

[15] J.H. Lunsford, The catalytic oxidative coupling of methane, Angewandte Chemie International Edition in English, 34 (1995) 970-980.

[16] Y. Lei, C. Chu, S. Li, Y. Sun, Methane Activations by Lanthanum Oxide Clusters, The Journal of

Physical Chemistry C, 118 (2014) 7932-7945.
[17] J.M. Deboy, R.F. Hicks, Kinetics of the oxidative coupling of methane over 1 wt% SrLa2O3, J. Catal., 113 (1988) 517-524.
[18] H. Borchert, M. Baerns, The Effect of Oxygen-Anion Conductivity of Metal–Oxide Doped Lanthanum Oxide Catalysts on Hydrocarbon Selectivity in the Oxidative Coupling of Methane, J. Catal., 168 (1997) 315-320.
[19] M. Chen, A.R. Felmy, D.A. Dixon, Structures and Stabilities of (MgO)n Nanoclusters, The Journal of Physical Chemistry A, 118 (2014) 3136-3146.
[20] X.-L. Ding, Z.-Y. Li, J.-H. Meng, Y.-X. Zhao, S.-G. He, Density-functional global optimization of $(La_2O_3)_n$ clusters, J. Chem. Phys., 137 (2012) 214311.
