# Supplementary material for: Active Slingshot Geometry Site on Single-Atom La Catalyst Largely Promotes Oxidative Methane Coupling
Source: ACS Cent Sci. 2025 Sep 20;11(11):2188–95. doi: 10.1021/acscentsci.5c01016 (PMC12670278; doi:10.1021/acscentsci.5c01016)
Supplement: Supplementary file 2 [file oc5c01016_si_002.pdf]

oc-2025-010165.R1

Name: Peer Review Information for "Active Slingshot Geometry Site on Single-Atom La Catalyst Largely Promotes Oxidative Methane Coupling"

First Round of Reviewer Comments

Reviewer: 1

Comments to the Author

This work reports an SA-La/MgO OCM catalyst, focusing on the investigation of its structure-activity relationship by utilizing advanced electron microscopy techniques combined with spectroscopic characterization. It is widely accepted that, for oxide-based catalysts, surface-active oxygen species are key in determining C2 selectivity in OCM reaction. The authors suggest that the unique La-O-Mg "slingshot" geometry in their designed catalyst is the crucial structure for activating oxygen and generating active oxygen species. Due to the high reaction temperature of OCM and the structural complexity of the catalysts, microscopic studies on the structure-activity relationship of OCM catalysts, especially in-situ microscopic studies, are rare. Although the chosen catalytic system may not have outstanding practical application potential, using it as a model system for atomic-level exploration still holds high theoretical significance for understanding the structure-activity relationship and rational design of catalysts. This article presents high-quality data and clear writing logic. I would support the publication of the present work after addressing the following points:

(1) The authors' discussion on pages 5-7, which utilizes STEM and XPS, XRD to elucidate the structure of the SA-La/MgO catalyst and the presence of single-atom La sites, requires further clarification. While the high-resolution electron microscopy provides valuable insights into the atomic-level structure, it offers a limited field of view, potentially overlooking the larger context of the material's morphology. This limitation necessitates the integration of macroscopic techniques to gain a comprehensive understanding. However, the current characterization data does not conclusively demonstrate the exclusive presence of single-atom La in SA-La/MgO or only particles in PA-La/MgO. Given the distinct

synthesis methods—sol-gel for SA-La/MgO and impregnation for PA-La/MgO, it is plausible that both catalysts contain a mixture of single atoms, clusters, and nanoparticles of La, albeit in varying proportions. The sol-gel method, while promoting atomic dispersion, doesn't preclude the possibility of La2O3 agglomeration during calcination. Conversely, the strong La-Mg interaction proposed by the authors could facilitate the formation of some single-atom La sites even in the impregnation-prepared PA-La/MgO. Therefore, to strengthen their argument, the authors should provide additional lower-magnification TEM/STEM images or EDX, providing a statistically relevant representation of the overall morphology and distribution of La species.

(2) The authors should include magnified regions of the XRD patterns. The current presentation in Figure 1d does not provide sufficient detail to discern key features.

(3) Regarding the XPS characterization of surface O and La species on both catalysts, it's crucial to acknowledge the potential presence of La2O2CO3, a commonly observed species in La2O3 catalysts. La2O3 is known to be susceptible to atmospheric contamination, readily reacting with impurities to form La2O2CO3. Therefore, when analyzing the XPS data for surface O and La species, the contribution of CO32- and La2O2CO3 should be carefully considered and appropriately deconvoluted. Furthermore, the assignment of the La-O peak in Figures 1e-f appears somewhat arbitrary. The peak is enveloped within a broader feature, and the raw data doesn't exhibit a distinct change in shape to justify the addition of this specific component.

(4) The duplication of the image in Figure 2a-b (identical to Figure 1c) introduces redundancy and should be addressed. Presenting the ex-situ HAADF and in situ ETEM data together in this manner could be misleading and should be reconsidered for clarity. Furthermore, rotating the original image in Figure 2b to better align with the simulated structure would significantly improve the visual comparison and facilitate interpretation.

(5) The ETEM results in Figure 2 raises several concerns. Firstly, it is unclear whether the zone axis of the sample remains consistent (as depicted in Figure 2c) after heating. The apparent atomic stretching and overlap observed in Figures 2d-e suggest a potential shift in the zone axis, which could significantly impact the interpretation of the images and the validity of the conclusions drawn. Secondly, the authors attribute the "dim sites" in Figure

2d to oxygen vacancies. However, the presence of numerous "dark sites" raises questions about their structural identity. Are these also oxygen vacancies, or do they represent different structural features? A more detailed analysis and discussion are needed to clarify this point. To substantiate the claim that the "dim sites" observed in the ETEM correspond to oxygen vacancies, further experiments are warranted. Switching the atmosphere to O_2 under the same conditions should provide valuable insights. If the "dim sites" disappear upon O_2 introduction, or exhibit structural oscillations with changing atmosphere, it would provide strong evidence supporting their assignment as oxygen vacancies.

(6) The authors should specify the precise regions where the in-situ EELS spectra were acquired.

(7) Figure 4b is used to demonstrate the regeneration of oxygen vacancies in the SA-La/MgO catalyst under OCM reaction conditions requires further clarification. Specifically, the authors should explicitly state whether the region analyzed in Figure 4b is identical to the one shown in Figure 2.

(8) This work is aimed at OCM reaction, fixed-bed catalytic performance data should be provided. In addition to TOF and C2 productivity, the information about CH_4 conversion and C2 selectivity is also very important.

Reviewer: 2

Comments to the Author

This manuscript introduces a single-atom La catalyst (SA-La/MgO) featuring a "slingshot" La-O-Mg geometry for oxidative coupling of methane (OCM). The authors claim that this unique structure activates lattice oxygen species (O_{act}^{2-}), enabling high C2 yields (5.96×10^{-2} mmol/(g·min)) and a 10-fold higher TOF compared to La_2O_3 nanoparticle-decorated MgO (PA-La/MgO). The work employs in situ ETEM and EELS to probe oxygen vacancy dynamics under reaction conditions. While the concept of geometric tuning for OCM enhancement is intriguing, the study suffers from insufficient mechanistic evidence,

inconsistent data interpretation, and inadequate characterization controls. Major revisions are required to validate the proposed mechanism and claims.

1. The claim that La^{3+} introduces excess electrons (page 9, line 13) appears problematic since La^{3+} was used in the material synthesis without any excess electrons. Furthermore, the higher valence state of La^{3+} compared to Mg^{2+} in the lattice doping would actually strengthen the La-O bond energy, making it unreasonable to simply assume that O in La-O-Mg is more reactive (assuming identical coordination numbers to MgO) or more prone to oxygen vacancy formation. Additionally, the statement on page 13 line 18 suggesting O_{act}^{2-} stabilizes La contradicts the earlier argument about O_{act}^{2-} being highly reactive.

2. Regarding page 10 lines 10-12, the observation that no oxygen vacancies were detected when SA-La/MgO was exposed to pure O_2 up to 700°C (Figure S6-S9) raises questions about the conclusion that methane reaction generates these vacancies. If vacancies weren't observed under reaction conditions, what evidence supports their formation?

3. Figures S6-S10 and S14-17 require more detailed analysis and labeling. Currently, it's impossible to distinguish La, O, and Mg features in these images. Proper identification of these elements is crucial for interpreting the results.

4. The repeated mentions of "Lattice O^{2-}" throughout the manuscript need experimental verification. Was EPR spectroscopy performed to confirm this species? The characterization appears incomplete without such evidence.

5. The characterization techniques seem relatively limited. Additional in situ techniques like XAS would provide more comprehensive insights into the electronic structure and local coordination environment of the active sites.

6. Several characterization aspects need improvement: a) Large-area TEM images of SA-La/MgO should be provided; b) Statistical analysis of the dimer sites claimed in Figure 2d; c) Line-scan EELS for the region in Figure 1c and similarly for Figures S5 and S17 to better support the elemental assignments.

7. The XPS signals in Figure S1 for PA-La/MgO and SA-La/MgO appear quite similar, making it difficult to conclusively demonstrate the single-atom nature of La in SA-La/MgO. More distinctive evidence is needed.

8. In the SI page 4, the reference to "Figure 2b" for ethane and ethylene yields appears incorrect as this figure doesn't show the described content.

9. The activation energy calculation in Figure S3 using only three temperature points is statistically insufficient (minimum four points required). Additionally, error bars should be provided for each temperature measurement.

10. The TOF calculation for PA-La/MgO should consider only surface La atoms rather than total La content, given the large particle size of La2O3. Furthermore, comparison with PA-La/MgO at identical loading (1 wt%) would strengthen the argument.

11. The catalytic activity should be benchmarked against literature values to properly contextualize the reported performance.

12. Details about the CrystalMaker modeling and construction parameters are missing and should be provided for reproducibility.

13. Minor corrections needed: a) Page 10 line 17 contains punctuation errors; b) The reference to "Figure 3a" on page 10 line 23 should be "Figure 3c".

14. The proposed surface site properties and elementary reaction steps would benefit significantly from supporting DFT calculations to provide theoretical validation of the experimental observations.

Author's Response to Peer Review Comments:

Dear editor,

many thanks for carefully handling our manuscript. We sincerely appreciate your time and effort, as well as the reviewers' thoughtful and constructive comments, which have helped us further improve our manuscript. We have performed several additional in situ ETEM and specstropy experiments and carefully revised the manuscript and the supporting information in accordance with the suggestions provided. Therefore, it took some time.

In the uploaded "Reviewers' comments reply"file, all formatting requirements have also been addressed accordingly.

The revised manuscript and new supporting information are uploaded as well.

We also appreciate the chance of compete the cover art of the issue, thererfore, we have updated the newly made cover art image.

We do hope the revision meet the publication standard of the ACS Central Science in this case.

Best and wish you a nice read.

Dear Editor

We sincerely appreciate your time and effort, as well as the reviewers' thoughtful and constructive comments, which have helped us further improve our manuscript. We have carefully revised the manuscript in accordance with the suggestions provided. All formatting requirements have also been addressed accordingly.

For clarity, the reviewer's comments are shown in black, while our responses are provided in blue. All changes made in the manuscript and the supporting information are highlighted in red. Our detailed responses to the reviewers' comments are presented below:

Reviewer: 1

Comments:

This work reports an SA-La/MgO OCM catalyst, focusing on the investigation of its structureactivity relationship by utilizing advanced electron microscopy techniques combined with spectroscopic characterization. It is widely accepted that, for oxide-based catalysts, surfaceactive oxygen species are key in determining C2 selectivity in OCM reaction. The authors suggest that the unique La-O-Mg "slingshot" geometry in their designed catalyst is the crucial structure for activating oxygen and generating active oxygen species. Due to the high reaction temperature of OCM and the structural complexity of the catalysts, microscopic studies on the structure-activity relationship of OCM catalysts, especially in-situ microscopic studies, are rare. Although the chosen catalytic system may not have outstanding practical application potential, using it as a model system for atomic-level exploration still holds high theoretical significance for understanding the structure-activity relationship and rational design of catalysts. This article presents high-quality data and clear writing logic. I would support the publication of the present work after addressing the following points:

Response: We are deeply grateful for the reviewer's efforts and the constructive comments to make this work to the highest possible quality. All of the comments have been carefully addressed and the manuscript has been revised point-by-point accordingly.

(1) The authors' discussion on pages 5-7, which utilizes STEM and XPS, XRD to elucidate the structure of the SA-La/MgO catalyst and the presence of single-atom La sites, requires further clarification. While the high-resolution electron microscopy provides valuable insights into the atomic-level structure, it offers a limited field of view, potentially overlooking the larger context of the material's morphology. This limitation necessitates the integration of macroscopic techniques to gain a comprehensive understanding. However, the current characterization data does not conclusively demonstrate the exclusive presence of single-atom La in SA-La/MgO or only particles in PA-La/MgO. Given the distinct synthesis methods— sol-gel for SA-La/MgO and impregnation for PA-La/MgO, it is plausible that both catalysts contain a mixture of single atoms, clusters, and nanoparticles of La, albeit in varying proportions. The sol-gel method, while promoting atomic dispersion, doesn't preclude the possibility of La2O3 agglomeration during calcination. Conversely, the strong La-Mg interaction proposed by the authors could facilitate the formation of some single-atom La sites even in the impregnation-prepared PA-La/MgO. Therefore, to strengthen their argument, the authors should provide additional lower-magnification TEM/STEM images or EDX, providing a statistically relevant representation of the overall morphology and distribution of La species.

Response: We sincerely appreciate the comments from reviewer about the structural differences between SA-La/MgO and PA-La/MgO. As suggested by reviewer, the HAADFSTEM images for SA-La/MgO and PA-La/MgO with lower magnification were added in supporting information Figure S2 The HAADF-STEM image of SA-La/MgO determines that no La_2O_3 cluster was observed in SA-La-MgO sample. In contrast, the HAADF-STEM image of PA-La/MgO determines that nanoparticles are supported on MgO. The low-magnification image of PA-La/MgO (Figure S2b) indicates the La_2O_3 are in the sheet-like morphology with average thickness as 7.45±1.43 nm. These new data indicates that La species are in atomic distribution in SA-La/MgO while the La species will form nanoparticles in PA-La/MgO. As suggested by reviewer, we also added the STEM-EDS mapping to investigate the distribution of La species on both samples. The EDS results indicate an evenly distributed La species on SA-LaMgO while the obvious La species aggregation can be observed on PA-LaMgO.

To address this issue, the following editing has been made in the manuscript on page 5 line 28:

“*The low magnification HAADF-STEM image for PA-La/MgO (Figure S2b) indicates that La species are in the form of flat sheet with average thickness is 7.45±1.43 nm. The XRD measurement of a particle size calculated via the Scherrer equation is approximately 18.3 nm [23] in Figure 1d*”

In the manuscript on page 6 line 9, the following editing has been made:

“*Low magnification image of SA-La/MgO (Figure S2a) indicates that no obvious La species aggregation, which is corresponding to the STEM EDS mapping (Figure S3) results that La is well dispersed on MgO without aggregation on SA-LaMgO.*”

In supporting information on page S4 line 6, the following content has been added:

“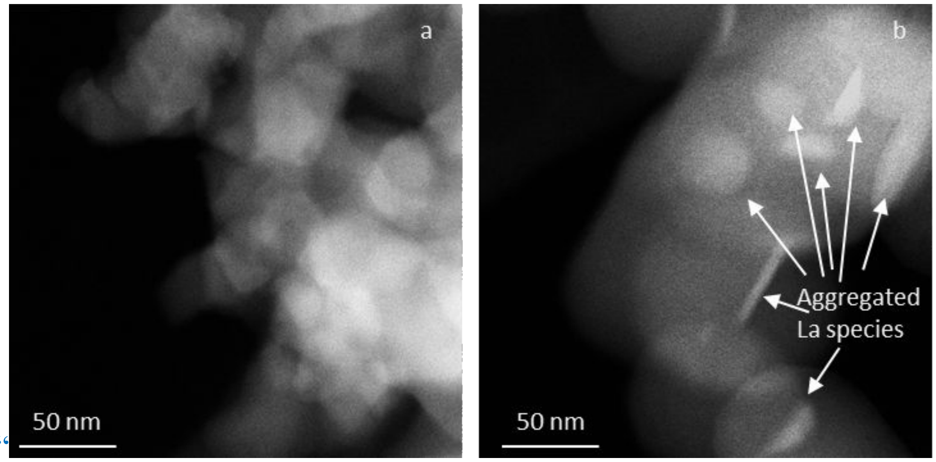

Figure S2 The low-magnification HAADF-STEM image of SA-LaMgO (a) and PA-LaMgO (b)

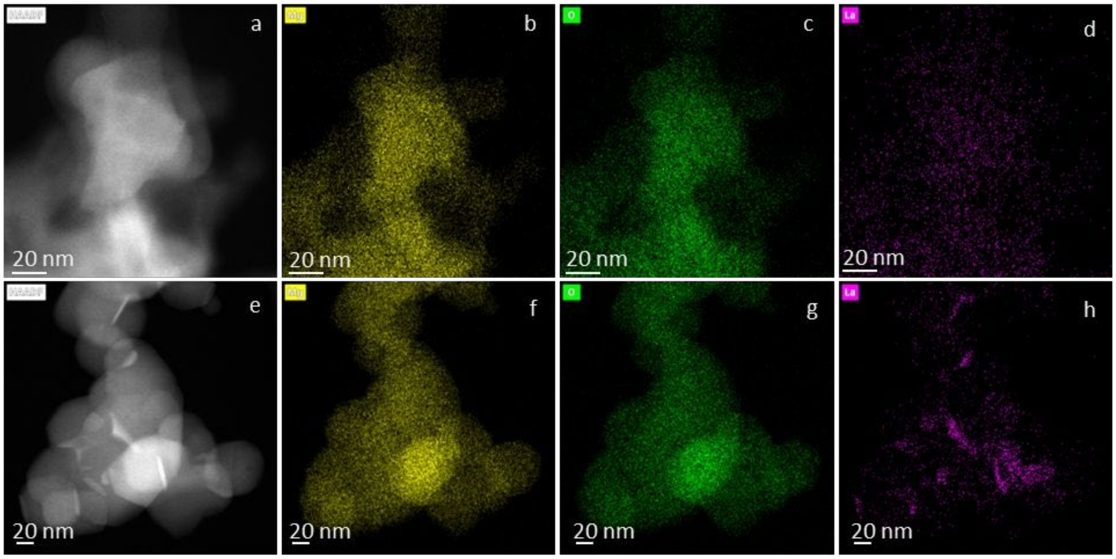

Figure S3 The HAADF-STEM image (a) and corresponding Mg (b), O (c), and La (d) EDS mapping for SA-LaMgO; The HAADF-STEM image (e), and corresponding Mg (f), O (g), and La (h) EDS mapping for PA-LaMgO

”

(2) The authors should include magnified regions of the XRD patterns. The current presentation in Figure 1d does not provide sufficient detail to discern key features. Response: We sincerely appreciate the reviewer’s comment regarding the XRD patterns. As suggested by reviewer, magnified regions of the XRD patterns have been included in the revised supporting information.

The following content has been added to supporting information on Page S4 line 1: “

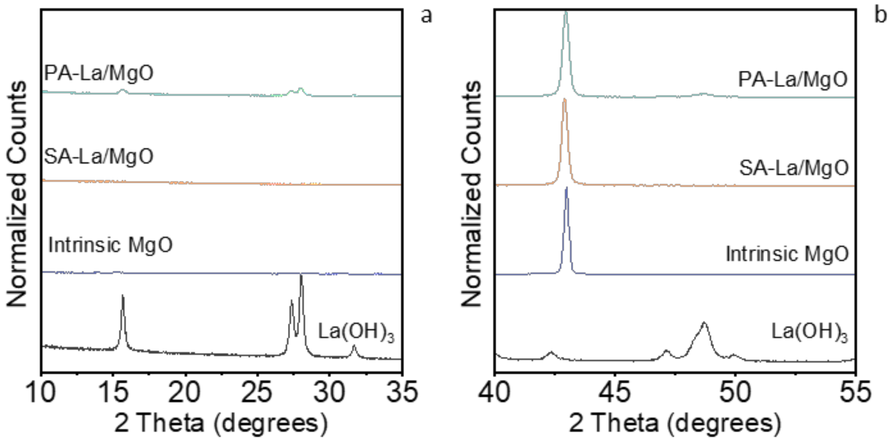

Figure S1 XRD patterns of PA-La/MgO, SA-La/MgO, Intrinsic MgO, and La(OH)$_3$ in the region between 10° to 35° (a) and 40° to 55° (b)."

The following content has been edited in the manuscript, on page 5 line 25

"*as also indicated by the XRD measurement in Figure 1d and Figure S1*"

(3) Regarding the XPS characterization of surface O and La species on both catalysts, it's crucial to acknowledge the potential presence of La2O2CO3, a commonly observed species in La2O3 catalysts. La2O3 is known to be susceptible to atmospheric contamination, readily reacting with impurities to form La2O2CO3. Therefore, when analyzing the XPS data for surface O and La species, the contribution of CO32- and La2O2CO3 should be carefully considered and appropriately deconvoluted. Furthermore, the assignment of the La-O peak in Figures 1e-f appears somewhat arbitrary. The peak is enveloped within a broader feature, and the raw data doesn't exhibit a distinct change in shape to justify the addition of this specific component.

Response: We are grateful for the reviewer's constructive comment about the XPS characterisation. After carefully investigation, we recognised that $La_2O_2CO_3$ and carbonate species would existed and this has been reported in a similar system that we investigate in our research.[2, 3] The O1s XPS of PA-LaMgO support the existence of carbonate species on the surface. Meanwhile, it is also noticed that $La_2O_2CO_3$ and La_2O_3 are not existence in SALaMgO system, which indicates the well La dispersion in the material. As suggested by reviewer, the following content has been edited in supporting information: In supporting information on page S5 line 3

"
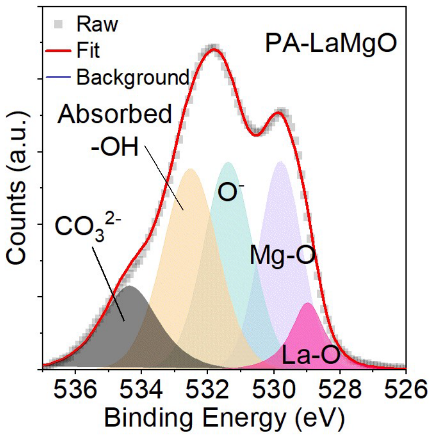

Figure S4 O 1s XPS result of PA-La/MgO

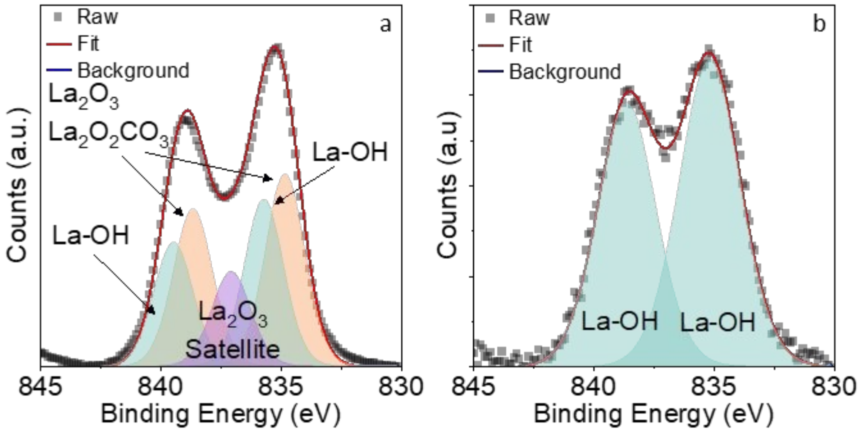

Figure S5 La3d XPS results of PA-La/MgO (a) and SA-La/MgO (b)

From the XPS results shown on Figure 1 (e) (f) and S5 (a) (b). The O1s spectrum of pure MgO can be deconvoluted into three peaks, listed as lattice O (Mg-O) at 530.0 eV[8], peroxide (O1) at 531.6 eV [8] and absorbed -OH at 532.5 eV[9]. After the lanthanum singe stie is introduced into the catalyst, the aforementioned peak position remains steady. Interestingly, the fraction of the surface -OH group decreased, and a new peak attributed to La-O which located at 528.9 eV is observed[10]. The carbonate group, which may come from CO_2 contamination, located at 534.4 eV was observed in O 1s XPS spectra for PA-LaMgO (Figure S4).[11] This result is corresponding to the published results.[12] As for the La 3d spectrum, the PA-La/MgO is applied as a reference and the result is shown on Figure S5 (a). The binding energy located between c.a. 830~840 eV is La3d 5/2 orbital [13]. Further analysis of the results indicates that two species are found in the sample. The peaks located at around 834.6 eV and 839.0 eV are attributed to the mixture of La_2O_3 and $La_2O_2(CO)_3$.[14] Apart from La_2O_3, the La-OH generated from La_2O_3 and moisture is another important component in the sample. The peaks for $La(OH)_3$ are located at around 835.2 eV and 838.8 eV [14]. It is indicated more surface $La(OH)_3$ species are observed on La 3d XPS spectra over SA-La/MgO compared to La_2O_3. It is corresponding to the XRD results that some lanthanum component on La/MgO are transferred to $La(OH)_3$ due to absorbing the vapor in the air. As for the SA-LaMgO (Figure S5b), the only observed La species is La-OH. The absence of

La_2O_3 and $La_2O_2(CO)_3$ suggests there was no La_2O_3 on SA-LaMgO surface, suggesting the well dispersion of La on SA-LaMgO."

(4) The duplication of the image in Figure 2a-b (identical to Figure 1c) introduces redundancy and should be addressed. Presenting the ex-situ HAADF and in situ ETEM data together in this manner could be misleading and should be reconsidered for clarity. Furthermore, rotating the original image in Figure 2b to better align with the simulated structure would significantly improve the visual comparison and facilitate interpretation.

Response: We thank the comment from reviewer regarding to the HAADF-STEM images. The Figure 1c was intended to determine the atomic distribution of La^{3+} ions in SA-La/MgO. As suggested by reviewer, the figure 1 c was updated, and we used another HAADF-STEM image for the same sample. The new figure 1c also indicates the atomically distribution of La^{3+} ions in SA-La/MgO. For the figure 2 a and b, we understand the concern regarding put in situ results and ex situ results in same figure. As suggested by reviewer, we have split the original figure 2 into new figure 2 and figure 3. We also appreciate the comment from reviewer about algin the HAADF results to simulation results. To address this, the HAADF-STEM images are rotated to have a better align with the simulated structure.

The following content has been edited in the manuscript:

On page 7, line 1:

“

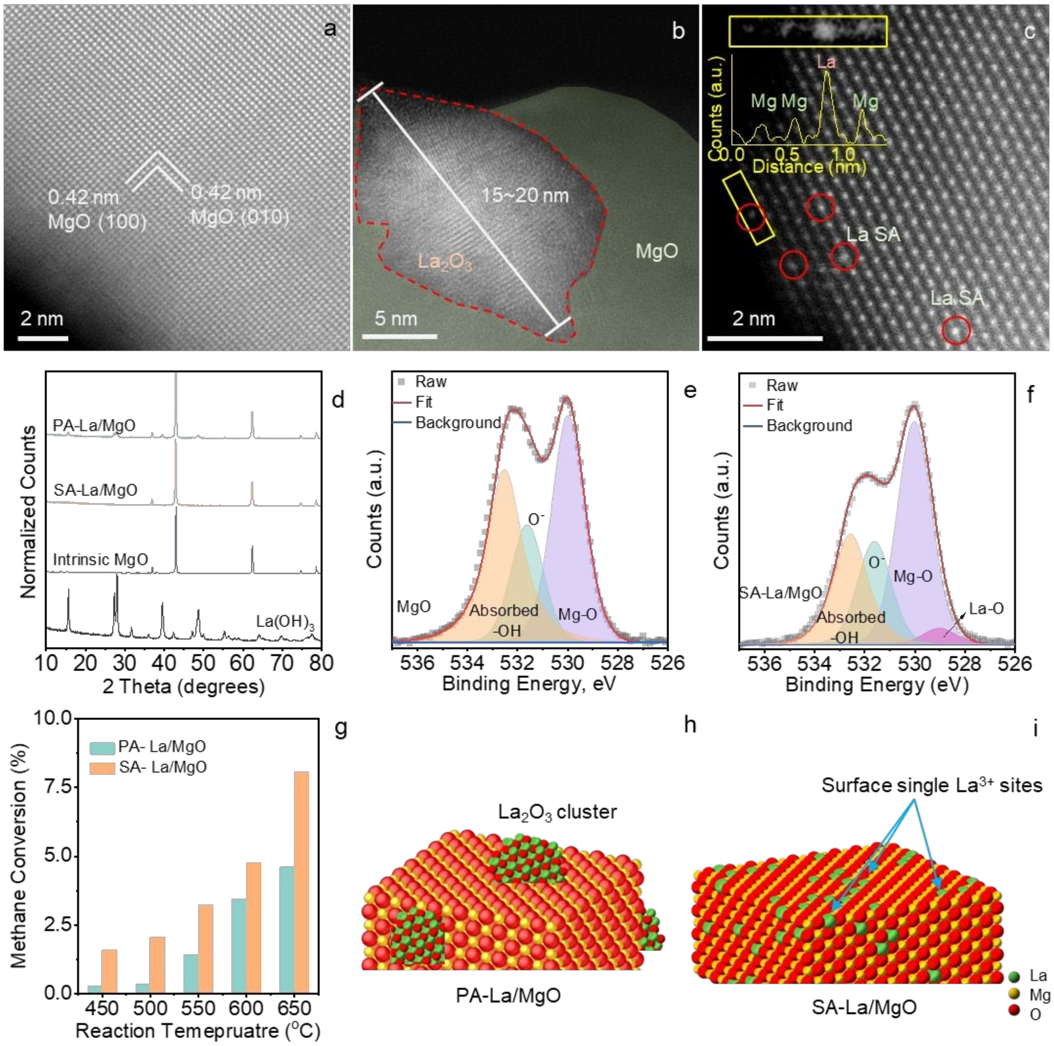

Figure 1 HAADF-STEM image for intrinsic MgO (a), PA-La/MgO (b) and SA-La/MgO (c); XRD pattern of PA-La/MgO, SA-La/MgO, Intrinsic MgO and $La(OH)_3$ (d); O1s XPS result of intrinsic MgO (e) and SA-La/MgO (f); Methane conversion at different temperature of PALa/MgO and SA-La/MgO based on DRIFTS product analysis(g); Illustration of the structure of PA-La/MgO (h) and SA-La/MgO (i).” On page 8, line 3:

“

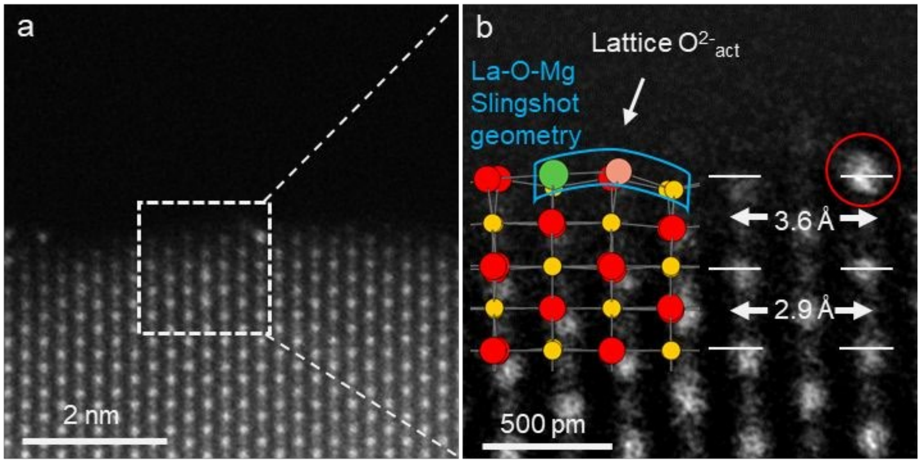

Figure 2 The HAADF-STEM image of SA-La/MgO (a) and the zoomed-in image of panel a (b), simulation of SA-La/MgO catalyst after relaxation was also added for comparison (Where green ball represent La, yellow ball represent Mg, red ball represent O, and pink ball represent active lattice O)"

On page9, line 14:

“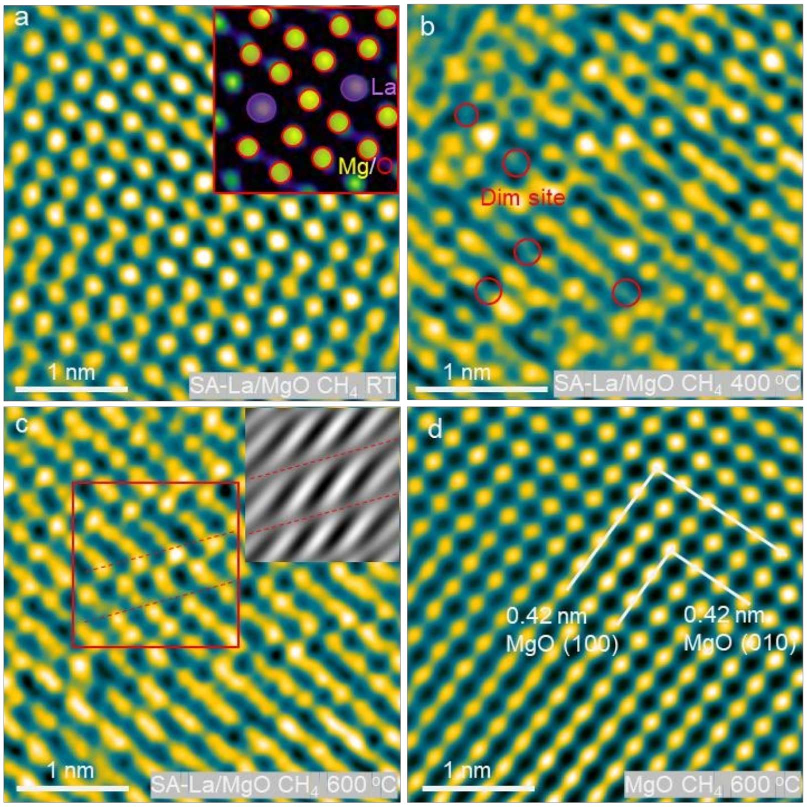

Figure 3 In situ ETEM image of SA-La/MgO in methane atmosphere at room temperature and the illustration of La single atom on SA-La/MgO (a); In situ ETEM image of SA-La/MgO in methane atmosphere at 400 ºC (b)and at 600 ºC (c) the IFFT image of selected area (red square) in panel e was also provided. The red dash line in panel c indicates the disturbed regions; In situ ETEM image of intrinsic MgO in methane atmosphere at 600 ºC (d)”

(5) The ETEM results in Figure 2 raises several concerns. Firstly, it is unclear whether the zone axis of the sample remains consistent (as depicted in Figure 2c) after heating. The apparent atomic stretching and overlap observed in Figures 2d-e suggest a potential shift in the zone axis, which could significantly impact the interpretation of the images and the validity of the conclusions drawn. Secondly, the authors attribute the "dim sites" in Figure 2d to oxygen vacancies. However, the presence of numerous "dark sites" raises questions about their structural identity. Are these also oxygen vacancies, or do they represent different structural features? A more detailed analysis and discussion are needed to clarify this point. To substantiate the claim that the "dim sites" observed in the ETEM correspond to oxygen vacancies, further experiments are warranted.

Switching the atmosphere to O2 under the same conditions should provide valuable insights. If the "dim sites" disappear upon O2 introduction, or exhibit structural oscillations with changing atmosphere, it would provide strong evidence supporting their assignment as oxygen vacancies.

Response: We deeply appreciate the reviewer's constructive suggestion to further investigate the "dim sites" and oxygen vacancies' oscillations. As suggested by reviewer, we conducted a supplementary in situ ETEM investigation by switching the atmosphere between O_2 and CH_4 at 400 °C and 600 °C. The results suggest that "dim sites" will be generated when exposed to CH_4 environment at elevated temperature. After switching the gas environment from CH_4 to O_2, the generated "dim sites" disappeared. We further counted the number of "dim sites" in the view field, and it oscillated upon the gas environment alternation in which SA-La/MgO exposed to. Combined with other characterisation results originally provided in the manuscript, it is confirmed that these "dim sites" are oxygen vacancies, indicating the CH_4 activation mechanism over SA-La/MgO.

To address this comment, the following content has been added to the manuscript, on page 10 line 28:

"*To further validate this mechanism, in situ ETEM was performed under alternating CH_4 and O_2 atmosphere. As shown in Figure 4a, the dim sites emerged on SA-La/MgO at 400 °C under CH_4 environment, indicating the formation of oxygen vacancies. Upon switching the gas environment from CH_4 to O_2, most of the dim sites disappeared. We further increased the temperature to 600°C and performed gas environment alternation. Similar behavior was observed in same area at 600°C (Figure 4c and d) and the quantitative evolution of dim site density under different condition is presented in Figure S15. These results reveal an oscillation in the number of dim sites depending on the gas environment, confirming the reversible generation and replenishment of oxygen vacancies during the reaction.*

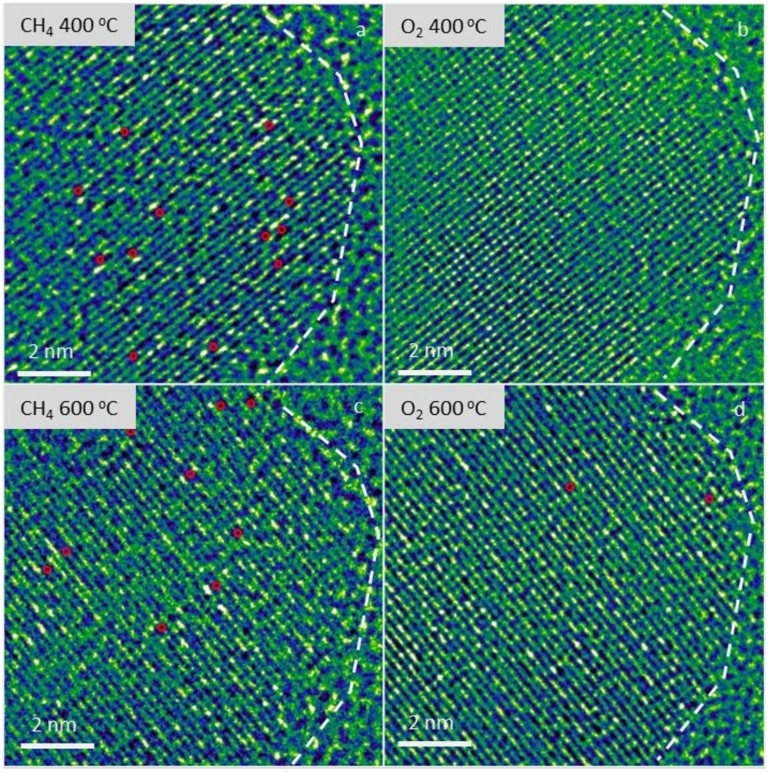

Figure 4 Fake-color high resolution in situ ETEM image of SA-La/MgO under alternating gas environment at elevated temperature: in CH_4 at 400 ºC for 616s (a); switched to O_2 and held at 400ºC for 1332s (b); temperature increased to 600 ºC and switched back to CH_4, held for 570s (c); switch to O_2 and held at 600 ºC for 416s (d). All observations were conducted at the same sample area. Red circles mark the position of "dim sites"."

The following content has been added to the supporting information, in page S13 line 1 "

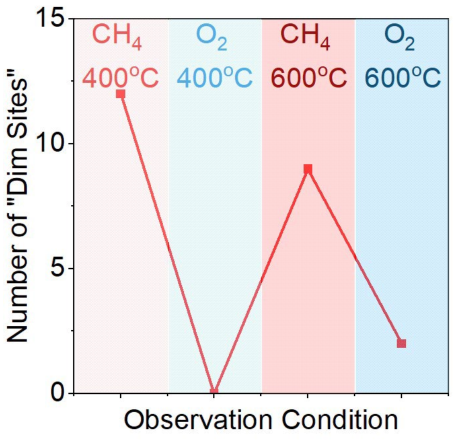

Figure S15 Profile of dim site density observed under different condition based on Figure 4. The observation was conducted at same area and the size is 10 nm x 10 nm"

(6) The authors should specify the precise regions where the in-situ EELS spectra were acquired.

Response: We sincerely thank the reviewer for the comment regarding to the regions that we acquired EELS spectra. The EELS spectra acquired from ex situ TEM characterisation is using STEM-EELS line scan or mapping and the region for the EELS analysis was given. However, for the in situ ETEM results, the EELS spectra were acquired in TEM mode, the beam diameter was about 10 micrometers and capture signals from the entire field of view. We understand our original description in methodology may lead to misunderstanding. To address this concern, we edit the methodology part. The following content has been edited on page 5 from line 4:

"*The in situ TEM-EELS spectra were taken from the edge region on the catalyst along with taking in situ images. The TEM-EELS was acquired at 27,000X magnification and the irradiation area was around 100 um^2. EELS signal was captured from the entire illuminated field.*"

(7) Figure 4b is used to demonstrate the regeneration of oxygen vacancies in the SA-La/MgO catalyst under OCM reaction conditions requires further clarification. Specifically, the authors should explicitly state whether the region analyzed in Figure 4b is identical to the one shown in Figure 2.

Response: We acknowledge the comments from reviewer about the figure to demonstrate the regeneration process in oxygen vacancies in the SA-La/MgO. Since the La species will aggregate after exposing in CH_4 environment, we have to change the sample after we conducted the experiment showing in Figure 2 (Figure 3 in revised manuscript), so the region analysed in Figure 4b (Figure 6b in revised manuscript) is not identical to the one shown in Figure 2. However, we analysed the typical region of the material. In all tests, we selected the thin region close to the edge of the samples. By doing so, we aimed to analyse areas with comparable composition and physicochemical properties to minimize the influence of sample variability.

To further address this issue, as suggested by reviewer, we added a supplementary in situ ETEM experiment to investigate the material dynamic change during exposing to different environments at the same area. This result indicates the oscillation of oxygen vacancies number under different

condition, which provides solid evidence of oxygen vacancies replenish under the effect of dioxygen.

The following content has been added to the manuscript:

In manuscript on page 10 line 28:

"*To further validate this mechanism, in situ ETEM was performed under alternating CH_4 and O_2 atmosphere. As shown in Figure 4a, the dim sites emerged on SA-La/MgO at 400 °C under CH_4 environment, indicating the formation of oxygen vacancies. Upon switching the gas environment from CH_4 to O_2, most of the dim sites disappeared. We further increased the temperature to 600°C and performed gas environment alternation. Similar behavior was observed in same area at 600°C (Figure 4c and d) and the quantitative evolution of dim site density under different condition is presented in Figure S15. These results reveal an oscillation in the number of dim sites depending on the gas environment, confirming the reversible generation and replenishment of oxygen vacancies during the reaction.*

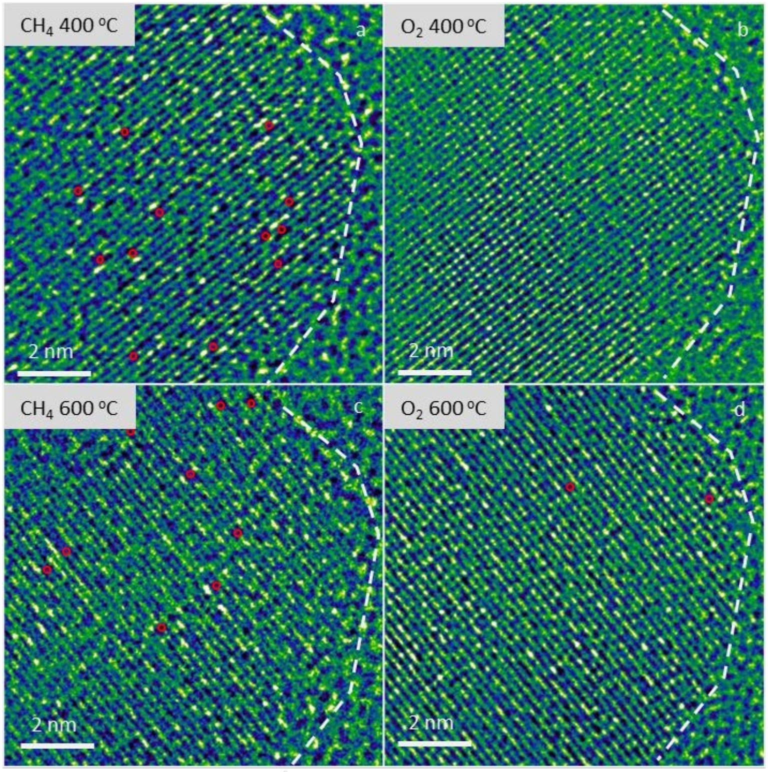

Figure 4 Fake-color high resolution in situ ETEM image of SA-La/MgO under alternating gas environment at elevated temperature: in CH_4 at 400 °C for 616s (a); switched to O_2 and held at

400°C for 1332s (b); temperature increased to 600 °C and switched back to CH_4, held for 570s (c); switch to O_2 and held at 600 °C for 416s (d). All observations were conducted at the same sample area. Red circles mark the position of "dim sites"."

(8) This work is aimed at OCM reaction, fixed-bed catalytic performance data should be provided. In addition to TOF and C2 productivity, the information about CH4 conversion and C2 selectivity is also very important.

Response: We sincerely acknowledge the reviewer's constructive comment about providing more catalytic performance data. The CH_4 conversion data over two different catalysts was originally given in Figure 1g. Since the C_2 yield is equal to ½ CH_4 conversion times C_2 selectivity, we believed that providing CH_4 conversion and C_2 yield were sufficient to demonstrate the fixed-bed catalytic performance. As suggested by reviewer, the C_2 selectivity was given in Figure S25.

The following content has been added to supporting information:

In supporting information on page S17 line 11

"
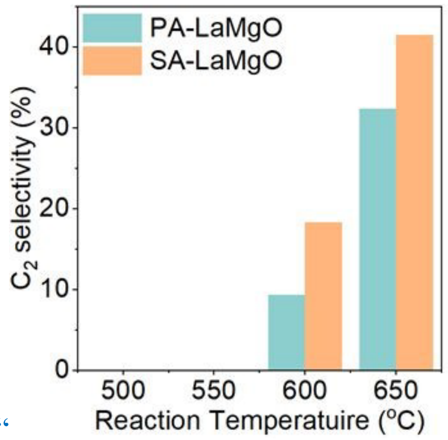

Figure S25 The C_2 selectivity over PA-LaMgO and SA-LaMgO" The following content has been edited in manuscript:

In the manuscript on page 17 line 18:

"*With this unique active site geometry over SA-La/MgO, the CH_4 conversion and C_2 selectivity (Figure S25) was improved*."

Reviewer: 2

Comments:

This manuscript introduces a single-atom La catalyst (SA-La/MgO) featuring a "slingshot" LaO-Mg geometry for oxidative coupling of methane (OCM). The authors claim that this unique structure activates lattice oxygen species (Oact2−), enabling high C2 yields (5.96×10−2 mmol/(g·min)) and a 10-fold higher TOF compared to La2O3 nanoparticle-decorated MgO (PA-La/MgO). The work employs in situ ETEM and EELS to probe oxygen vacancy dynamics under reaction conditions. While the concept of geometric tuning for OCM enhancement is intriguing, the study suffers from insufficient mechanistic evidence, inconsistent data interpretation, and inadequate characterization controls. Major revisions are required to validate the proposed mechanism and claims.

Reply: We sincerely appreciate the reviewer's critical evaluation and thoughtful suggestions, which have significantly contributed to enhancing the quality of our manuscript. Each comment has been carefully considered and addressed in detail.

1. The claim that La3+ introduces excess electrons (page 9, line 13) appears problematic since La3+ was used in the material synthesis without any excess electrons. Furthermore, the higher valence state of La3+ compared to Mg2+ in the lattice doping would actually strengthen the La-O bond energy, making it unreasonable to simply assume that O in La-O-Mg is more reactive (assuming identical coordination numbers to MgO) or more prone to oxygen vacancy formation. Additionally, the statement on page 13 line 18 suggesting Oact2- stabilizes La contradicts the earlier argument about Oact2- being highly reactive.

Reply: We thank the reviewers comment regarding the material structure and function of activated surface lattice oxygen O_{act}^{2-}. According to published results, doping high-valence atoms into metal oxides will produces excess electrons, and making the material to electron donor.[4, 5] This may resultant from the formation of cation vacancies due to the introduction of high-valence dopant.[6] So, the introduction of La^{3+} will alter the localised electron structure and thus improve the OCM reaction performance. As or the La-O bond energy, it is true that bonding energy of intrinsic La_2O_3 is much higher than MgO,[7] which aligns with our purposed mechanism and previously published results that OCM over intrinsic La_2O_3 will not generate oxygen defects.[8]

However, in our study, the La cations are doped into MgO, which have significant differences to intrinsic La_2O_3 system. Since La cation has much larger effective radius than Mg cation,[9] the doped La cation exerts lattice strain on adjacent atoms, thereby distorting the localized crystal structure and forming "slingshot geometry site". This phenomenon was observed in our HAADF-STEM results and newly added iDPC results. Since the geometry change would significantly change the bonding energy,[10] it is plausible that the La-O bonding energy could be changed by the slingshot geometry and make the O adjacent to La more active.

We understand the current description in page 9 could potentially make confusion. To address this issue, we modified the following description in the manuscript:

On page 9 line 7:

"*The doped La^{3+} in the MgO lattice alters the localised electron structure of the surface La-O-Mg geometry [27].*"

As for the stabilisation effect of O_{act}^{2-}, the reviewer might have misunderstanding regarding to our explanation. Our purposed O_{act}^{2-} is highly active is not conflicting with O_{act}^{2-} stabilising doped La^{3+}. We purpose the reaction utilise lattice oxygen and undergo Mars-van Krevelen mechanism, meaning the O_{act}^{2-} will be consumed during reaction with CH_4 and then refilled by migration of adsorbed oxygen species.[11] When the sample is exposed to a CH_4 environment, O_{act}^{2-} reacts with CH_4 but is difficult to replenish once consumed. Consequently, considerable amounts of O_{act}^{2-} around La^{3+} was lost, resulting in the destabilisation and eventual aggregation of La^{3+}. In contrast, when the sample is exposed to a CH_4/O_2 mixture, O_{act}^{2-} still reacts with CH_4, but the consumed O_{act}^{2-} is rapidly replenished by adsorbed dioxygen molecules. As a result, O_{act}^{2-} remains in dynamic equilibrium, preventing significant loss of O_{act}^{2-} around La^{3+} and thereby ensuring the stability of the doped La^{3+} within the MgO matrix. This process is confirmed by the supplementary in situ ETEM results shown in Figure 4 in the revised manuscript.

2. Regarding page 10 lines 10-12, the observation that no oxygen vacancies were detected when SA-La/MgO was exposed to pure O2 up to 700°C (Figure S6-S9) raises questions about the

conclusion that methane reaction generates these vacancies. If vacancies weren't observed under reaction conditions, what evidence supports their formation?

Response: We sincerely appreciate the comments from reviewer about the oxygen vacancies generation. The reviewer might have misunderstanding regarding to this part. In our purposed mechanism, it is CH_4 reacted with O_{act}^{2-} and generate vacancies. Figure S6-S9 (Figure S11-14 in revised supporting information) displays the material exposed in O_2 atmosphere. Since there is no CH_4 involved, the oxygen vacancies cannot be generated under this condition. In contrast, the oxygen vacancies can be found in situ ETEM results of SA-La/MgO exposed in pure CH_4 environment (Figure 3 b and c). This result is well-algin with our purposed mechanism. To further address this concern, we have a supplementary in situ ETEM results added into the revised manuscript. The new results provide further evidence determined the oxygen vacancies generation-diminishing oscillations upon SA-La/MgO switching in CH_4 and O_2 environments at elevated temperatures.

To address this concern, the following content has been added to the manuscript:

In manuscript on page 10 line 28:

"*To further validate this mechanism, in situ ETEM was performed under alternating CH_4 and O_2 atmosphere. As shown in Figure 4a, the dim sites emerged on SA-La/MgO at 400 ^oC under CH_4 environment, indicating the formation of oxygen vacancies. Upon switching the gas environment from CH_4 to O_2, most of the dim sites disappeared. We further increased the temperature to 600^oC and performed gas environment alternation. Similar behavior was observed in same area at 600^oC (Figure 4c and d) and the quantitative evolution of dim site density under different condition is presented in Figure S15. These results reveal an oscillation in the number of dim sites depending on the gas environment, confirming the reversible generation and replenishment of oxygen vacancies during the reaction.*

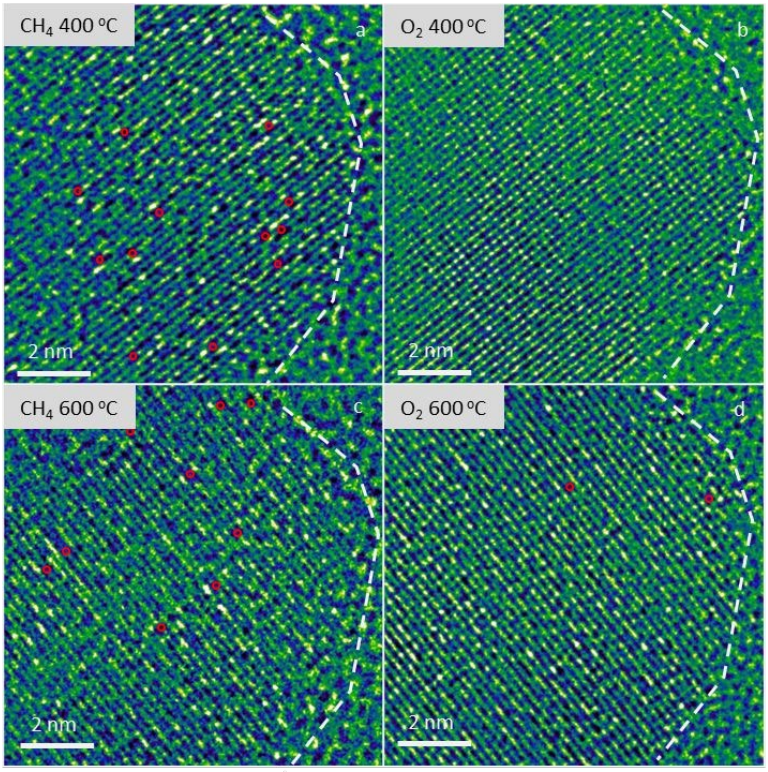

Figure 4 Fake-color high resolution in situ ETEM image of SA-La/MgO under alternating gas environment at elevated temperature: in CH_4 at 400 °C for 616s (a); switched to O_2 and held at 400°C for 1332s (b); temperature increased to 600 °C and switched back to CH_4, held for 570s (c); switch to O_2 and held at 600 °C for 416s (d). All observations were conducted at the same sample area. Red circles mark the position of "dim sites"."

3. Figures S6-S10 and S14-17 require more detailed analysis and labeling. Currently, it's impossible to distinguish La, O, and Mg features in these images. Proper identification of these elements is crucial for interpreting the results.

Response: We appreciate the constructive comment from reviewer regarding to labelling the ETEM figures in the supporting information. We managed to label most of the images in supporting information. However, due to the sample movement and vibration during test as well as overlapped particles, some of the images were influenced by Moier fringes or only showing lattice fringes, making difficulties for element label.

The following content has been edited in Supporting information

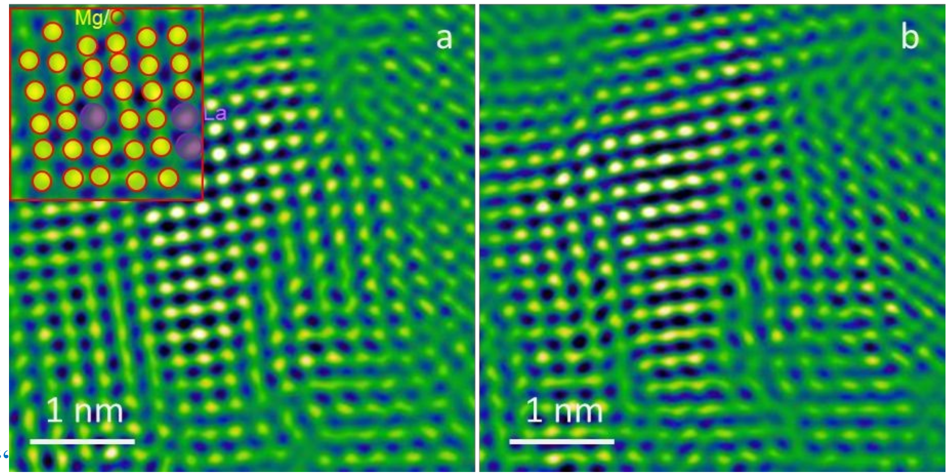

Figure S11 In-situ ETEM images of SA-La/MgO in oxygen atmosphere at room temperature for 0s (a) and 20s (b)

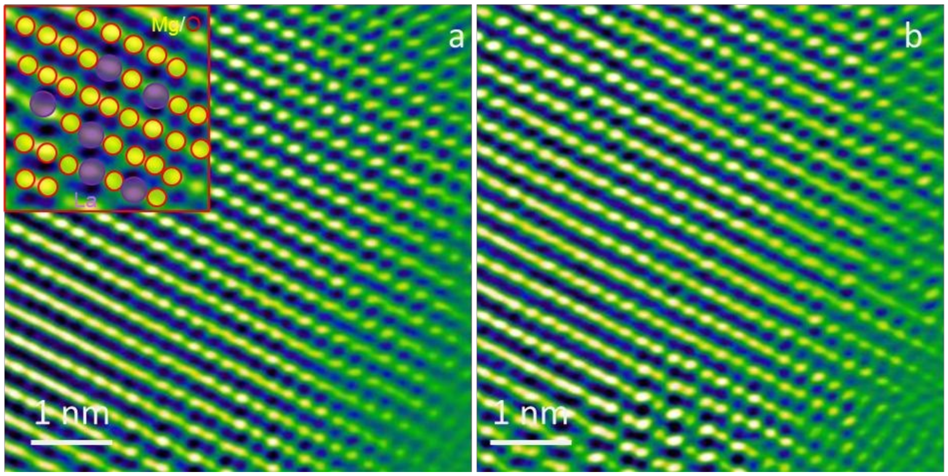

Figure S13 In-situ ETEM images of SA-La/MgO in oxygen atmosphere at 600 ºC for 0s (a) and 20 s (b)

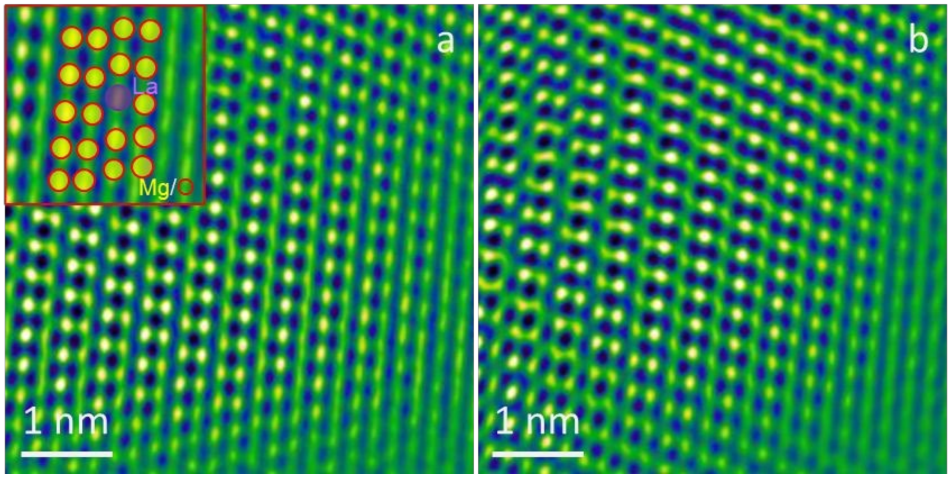

Figure S14 In-situ ETEM images of SA-La/MgO in oxygen atmosphere at 700 °C for 0s (a) and 40s (b)

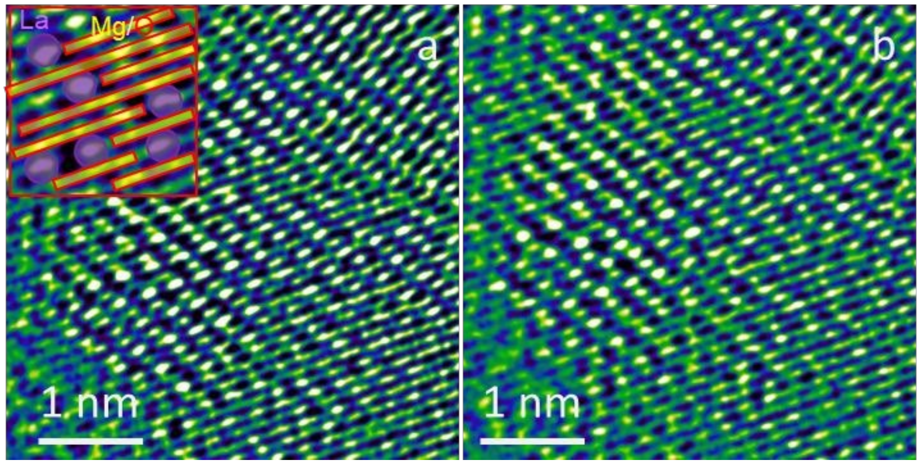

Figure S20 In-situ ETEM images of SA-La/MgO in methane and oxygen atmosphere at room temperature for 0s (a) and 5s (b)

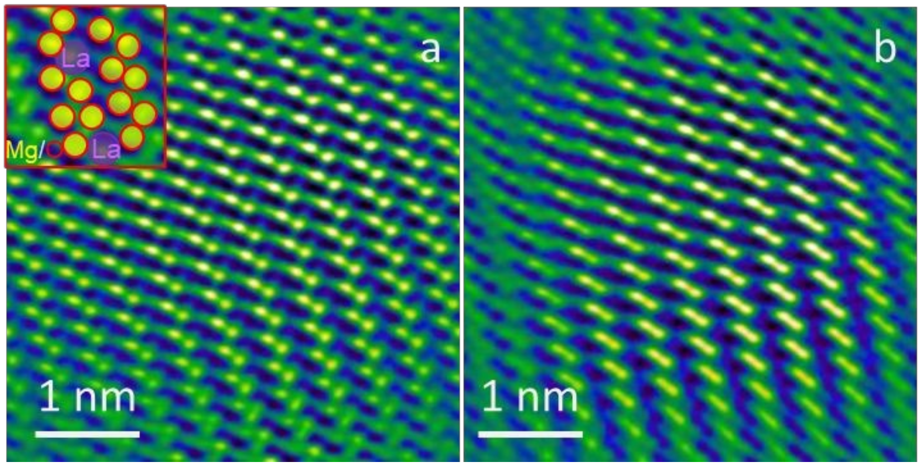

Figure S21 In-situ ETEM images of SA-La/MgO in methane and oxygen atmosphere at 300 °C for 0s (a) and 5s (b)"

4. The repeated mentions of "Lattice O2-" throughout the manuscript need experimental verification. Was EPR spectroscopy performed to confirm this species? The characterization appears incomplete without such evidence.

Response: We thank the comment from author regarding analyse the lattice O^{2-} . Considering lattice O^{2-} does not have unpair electrons, the EPR may not be the best approach to analyse it[3].

However, as pointed out by reviewer, it is crucial to identify lattice O^{2-}_{act} in our work as it is the key for our reaction. In our original manuscript, we used the EELS results determined a special lattice O^{2-} adjacent to La ion in fresh SA-LaMgO. To further confirm this observation, we used ex situ integrated differential phase contrast (iDPC) image to investigate the SALaMgO. We observed the lattice O^{2-} adjacent La ion was forming slingshot geometry at multiple sites. We believe this is direct evidence that "lattice O^{2-}" in MgO forming slingshot geometry with the influence of La single atom.

To address this issue, the following content has been added to the supporting information on page S10 line 1:

“

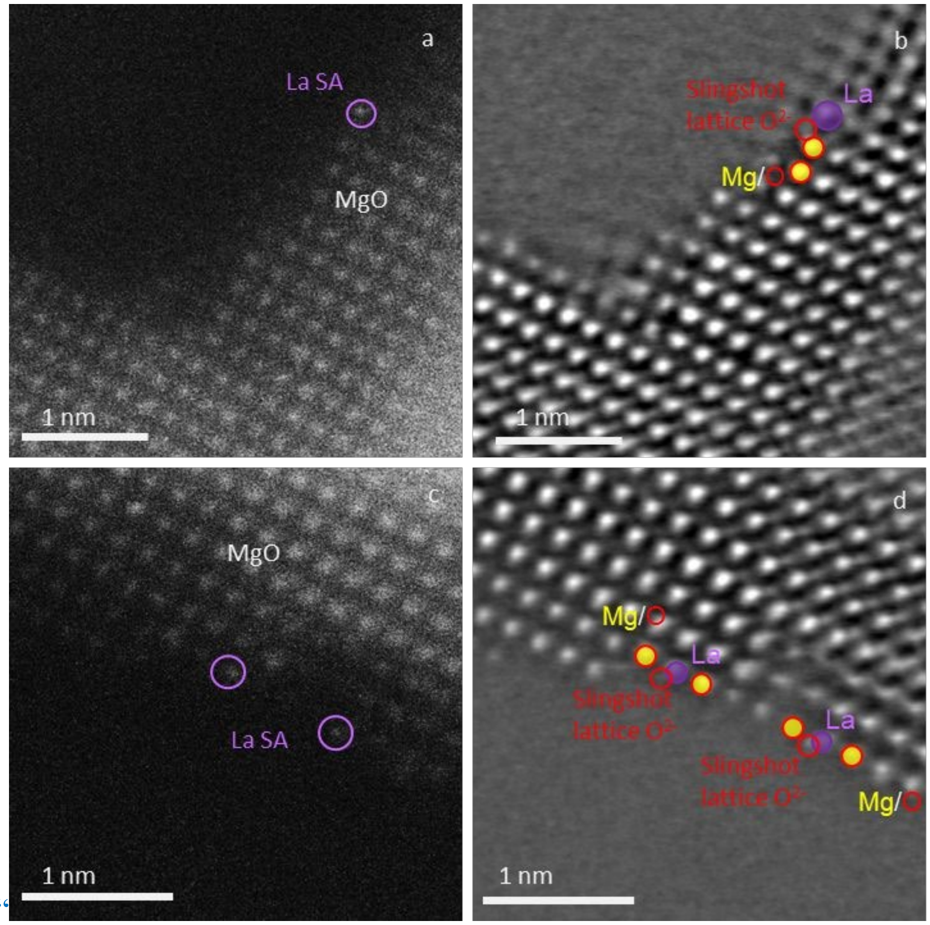

Figure S8 The HAADF-STEM image of SA-LaMgO (a) and the corresponding STEM-iDPC image of panel a (b); Another site HAADF-STEM image of SA-LaMgO (c) and the corresponding STEM-iDPC image of panel c (d). The STEM-iDPC image directly reveals the lattice O^{2-} in slingshot La-O-Mg geometry.”

The following content has been edited/added to manuscript:

On page 4 line 22, the following content has been edited:

"*High resolution High Angle Annular Dark Filed (HAADF) images and integrated differential phase contrast (iDPC) images were taken on the FEI Themis Z equipped with probe and image spherical aberration corrector on 300 kV*."

On page 8 line 21, the following content has been added:

"*STEM-iDPC image shown on Figure S8 revealed the surface lattice O^{2-} adjacent to La ions was displaced from its original position. This direct observation double confirms the formation of 'slingshot' type of La-O-Mg coordination geometry*."

5. The characterization techniques seem relatively limited. Additional in situ techniques like XAS would provide more comprehensive insights into the electronic structure and local coordination environment of the active sites.

Response: We sincerely appreciate the comments from reviewer about using other techniques to investigate the electronic structure and local coordination environment of the active sites. In our original manuscript, we used in situ EELS to investigate the electronic structure of our material. It is recognised as an effective way to reveal the electronic structure.[12, 13] In this work, it is proven by EELS that La-O bond was formed at in SA-LaMgO and the oxidation status change of La species during the reaction. As for the local coordination environment, due to the beamtime limitation, unfortunately, we are unable to provide additional in situ XAS test. To address this issue, ex situ extended electron energy loss fine structure (EXELFS) spectra for SA-La/MgO before and after exposed to CH_4 were acquired. The R-space O1s EXELFS indicated two peaks in the region between 2-3 Å. The peak located at around 2.1 Å is attributed to O-Mg path,[14] whereas the peak located between 2.5 to 2.8 Å is attributed to O-La path.[15] This result determines that the reduction of La-O coordination after SA-La/MgO exposed in CH_4 environment.

The following content has been added to the supporting information on Page S16 line 9: "

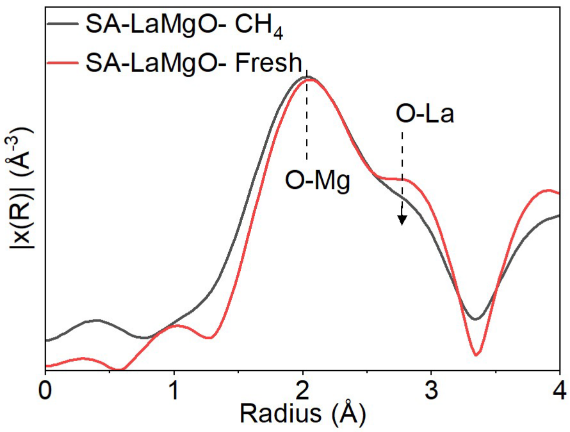

Figure S23 the extended electron energy loss fine structure (EXELFS) spectrum of SA-LaMgO before and after exposed to CH_4.

The R-space O1s EXELFS indicated two peaks in the region between 2-3 Å. The peak located at around 2.1 Å is attributed to O-Mg path,[19] whereas the peak located between 2.5 to 2.8 Å is attributed to O-La path.[20] This result determines that the reduction of La-O coordination after SA-La/MgO exposed in CH4 environment."

The following content has been added to the manuscript, on page 15 line 2:

"*Furthermore, the R-space O K edge extended electron energy loss fine structure spectrum for SA-LaMgO before and after exposure to CH_4 (Figure S23) determines the O-La coordination declination after SA-LaMgO heated in CH_4 environment, suggesting the O_{act}^{2-} in La-O-Mg was reacted with CH_4.*"

6. Several characterization aspects need improvement: a) Large-area TEM images of SALa/MgO should be provided; b) Statistical analysis of the dimer sites claimed in Figure 2d; c) Line-scan EELS for the region in Figure 1c and similarly for Figures S5 and S17 to better support the elemental assignments.

Response: We appreciate the reviewer's comment regarding improve the characterisations.

a) As suggested by reviewer, the large-area STEM images for both SA-La/MgO and PALa/MgO were provided. The large-area STEM indicates no La aggregation was observed in SA-La/MgO while the nanoparticles of La species was observed in PA-La/MgO.

The following content has been added to the supporting information on page S4 line 6:

“

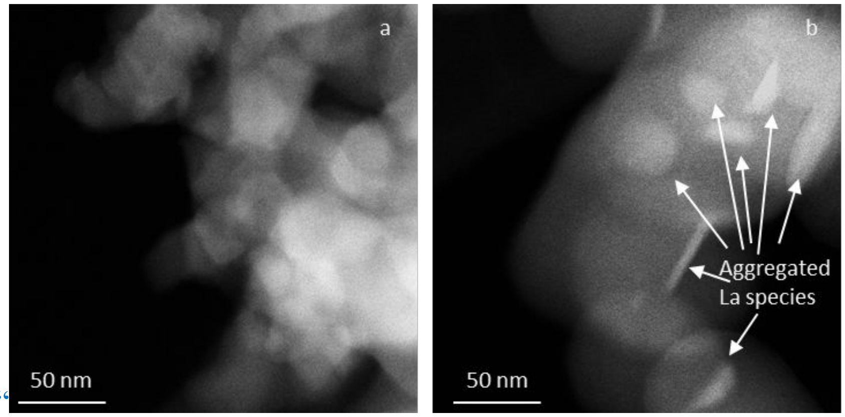

Figure S2 The low-magnification HAADF-STEM image of SA-LaMgO (a) and PA-LaMgO (b) ”

The following content has been added to the manuscript for describing the low-magnification HAADF-STEM images. On page 5 line 28:

“*The low magnification HAADF-STEM image for PA-La/MgO (Figure S2b) indicates that La species are in the form of flat sheet with average thickness is 7.45±1.43 nm. The XRD measurement of a particle size calculated via the Scherrer equation is approximately 18.3 nm [23] in Figure 1d*.”

b) To address the concern regarding to the formation and disappearing of dimer sites and its statistical analysis, we conducted a supplementary in situ ETEM experiment and switching the atmosphere between O_2 and CH_4 at 400 °C and 600 °C. We observed the same area under 4 different conditions and confirms the formation of “dim site” upon CH_4 exposure and these “dim site” disappeared after the sample exposed in O_2. We statistical analysis the dimer sites in this figure and it confirms its oscillation depending on gas environment.

The following content has been added to the manuscript on page 10 line 28 :

"To further validate this mechanism, in situ ETEM was performed under alternating CH_4 and O_2 atmosphere. As shown in Figure 4a, the dim sites emerged on SA-La/MgO at 400 °C under CH_4 environment, indicating the formation of oxygen vacancies. Upon switching the gas environment from CH_4 to O_2, most of the dim sites disappeared. We further increased the temperature to 600°C and performed gas environment alternation. Similar behavior was observed in same area at 600°C (Figure 4c and d) and the quantitative evolution of dim site density under different condition is presented in Figure S15. These results reveal an oscillation in the number of dim sites depending on the gas environment, confirming the reversible generation and replenishment of oxygen vacancies during the reaction.

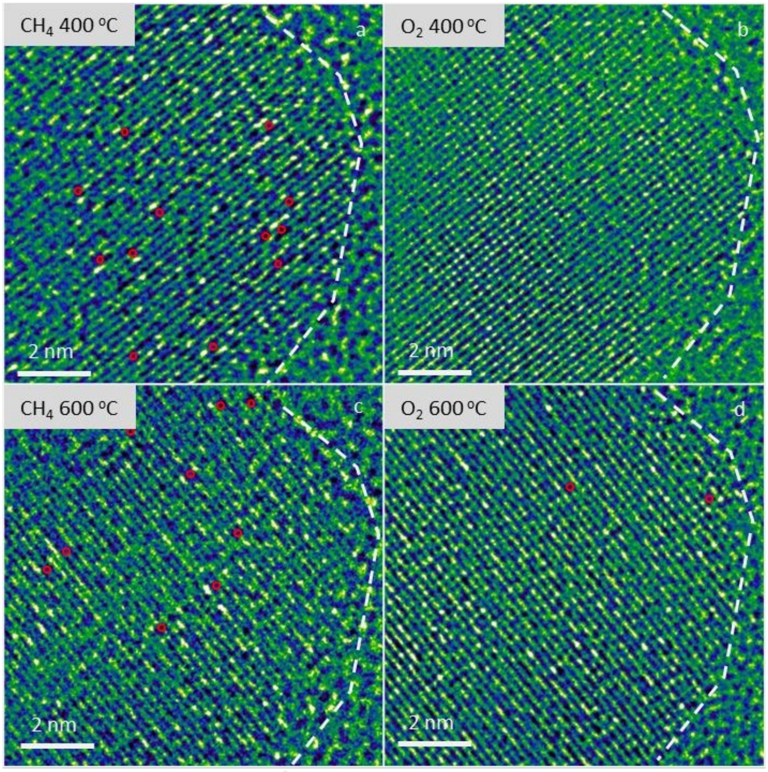

Figure 4 Fake-color high resolution in situ ETEM image of SA-La/MgO under alternating gas environment at elevated temperature: in CH_4 at 400 °C for 616s (a); switched to O_2 and held at 400°C for 1332s (b); temperature increased to 600 °C and switched back to CH_4, held for 570s (c); switch to O_2 and held at 600 °C for 416s (d). All observations were conducted at the same sample area. Red circles mark the position of "dim sites"."

The following content has been added to the supporting information in page S13 line 1:

“

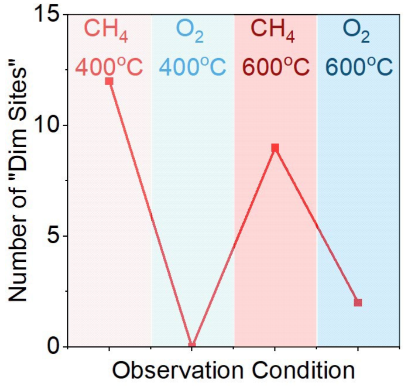

Figure S15 Profile of dim site density observed under different condition based on Figure 4. The observation was conducted at same area and the size is 10 nm x 10 nm”

c) As for the single atom system, as we do not have the access to low-dose camera (i.e. K2 or K3 camera), after a few attempts, we found that the line-scan EELS could not give us enough signal strength while keeping the sample free from beam damage for this single atom system. To better understand the distribution of the different elements, we analysed the STEM-EDS mapping for SA-LaMgO and PA-LaMgO. The results determined a well dispersed La in SA-LaMgO while the aggregated La was observed in PA-LaMgO

The following content has been added to the supporting information on page S4 line 6.

“

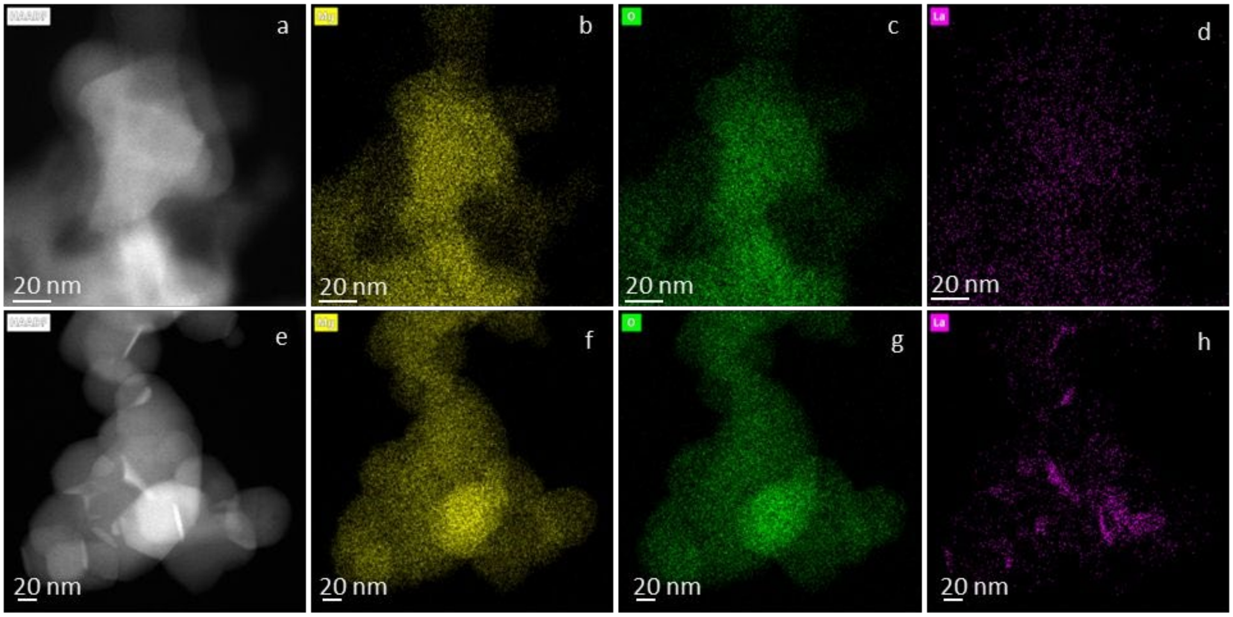

Figure S2The HAADF-STEM image (a) and corresponding Mg (b), O (c), and La (d) EDS mapping for SA-LaMgO; The HAADF-STEM image (e), and corresponding Mg (f), O (g), and La (h) EDS mapping for PA-LaMgO"

The following content has been added to the manuscript for describing the STEM-EDS mapping. In page 6 line 8:

"*Low magnification image of SA-La/MgO (Figure S2a) indicates that no obvious La species aggregation, which is corresponding to the STEM EDS mapping (Figure S3) results that La is well dispersed on MgO without aggregation on SA-LaMgO.*"

7. The XPS signals in Figure S1 for PA-La/MgO and SA-La/MgO appear quite similar, making it difficult to conclusively demonstrate the single-atom nature of La in SA-La/MgO. More distinctive evidence is needed.

Response: We appreciate the comment from reviewer regarding to the XPS analysis. After carefully analysis, we revised our peak assignment based on some published results which is more closely related to our research system. In the new assignment, the La 3d XPS result for SA-LaMgO is only constituted of La-OH species while La_2O_3 species are not observed. For the PA-LaMgO, the La species is constituted of La-OH, La_2O_3, and $La_2O_2CO_3$. Combined with other

characterisation results like high-magnification HAADF-STEM images and newly added low-magnification HAADF-STEM images, it is evident that La in SA-LaMgO is atomically dispersed.

The following content has been edited in supporting information:

In supporting information on page S5 line 7:

"

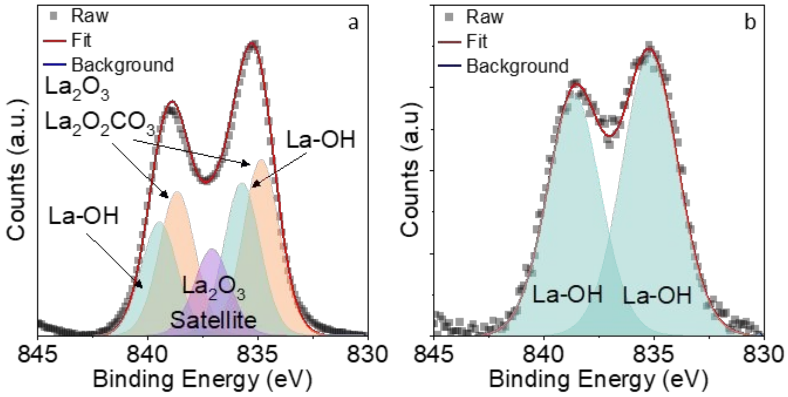

Figure S5 La3d XPS results of PA-La/MgO (a) and SA-La/MgO (b)

"

In supporting information on page S5 line 18:

"*As for the La 3d spectrum, the PA-La/MgO is applied as a reference and the result is shown on Figure S5 (a). The binding energy located between c.a. 830~840 eV is La3d 5/2 orbital [13]. Further analysis of the results indicates that two species are found in the sample. The peaks located at around 834.6 eV and 839.0 eV are attributed to the mixture of La_2O_3 and $La_2O_2(CO)_3$.[14] Apart from La_2O_3, the La-OH generated from La_2O_3 and moisture is another important component in the sample. The peaks for $La(OH)_3$ are located at around 835.2 eV and 838.8 eV [14]. It is indicated more surface $La(OH)_3$ species are observed on La 3d XPS spectra over SA-La/MgO compared to La_2O_3. It is corresponding to the XRD results that some lanthanum component on La/MgO are transferred to $La(OH)_3$ due to absorbing the vapor in the air. As for the SA-LaMgO (Figure S5b), the only observed La species is La-OH. The absence of La_2O_3 and $La_2O_2(CO)_3$ suggests there was no La_2O_3 on SA-LaMgO surface, suggesting the well dispersion of La on SA-LaMgO.*"

8. In the SI page 4, the reference to "Figure 2b" for ethane and ethylene yields appears incorrect as this figure doesn't show the described content.

Response: We thank the reviewer point this issue out and we apologise for our oversight. The following content in supporting information has been revised:

In supporting information page S4, line 5:

"*As for the yield of ethane and ethylene products displayed on Scheme 1 b*".

9. The activation energy calculation in Figure S3 using only three temperature points is statistically insufficient (minimum four points required). Additionally, error bars should be provided for each temperature measurement.

Response: We sincerely appreciate the reviewer's comment about activation energy calculation. As suggested by reviewer, we've recalculated the activation energy based on 4 data points and the error bar has been provided. The trend shown in original manuscript was not changed.

The following content in supporting information has been revised on page S9 line 1

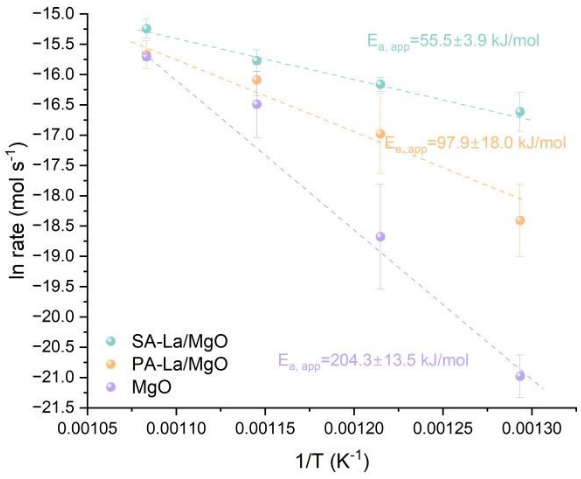

"

Figure S7 The apparent activation energy of methane conversion over SA-La/MgO, PALa/MgO, and intrinsic MgO. The calculation was based on the reaction performance shown on Figure 1g and Figure S6."

To correspond the change of Figure S7, the following content has been updated in manuscript:

On page 6 line 22

"*As depicted in Figure S7, the apparent methane activation energy over SA-La/MgO is 55.5±3.9 kJ/mol, which is notably lower than that over PA-La/MgO (97.9±18.0 kJ/mol) or intrinsic MgO (204.3±13.5 kJ/mol).*"

10. The TOF calculation for PA-La/MgO should consider only surface La atoms rather than total La content, given the large particle size of La2O3. Furthermore, comparison with PALa/MgO at identical loading (1 wt%) would strengthen the argument.

Response: We acknowledge the constructive comment from reviewer regarding to the TOF calculation and comparing sample selection.

For the TOF calculation, since the La_2O_3 dispersion is difficult to be determined by CO-TPD or H_2-TPD, we used TEM and XRD to estimate the particle size and calculate the surface La atom dispersion.

The average diameter calculated from XRD is 18.3 nm, and the corresponding La_2O_3 volume is 3208.9 nm^3. We noticed that aggregated La species in PA-LaMgO is flat sheet and the thickness of the sheet is 7.45±1.43 nm. Assuming the sheet is cylindrical, then the surface area of the La_2O_3 is 1413.3 nm^2. Since the volume of a La_2O_3 unit cell is 0.821 nm^3,[16] there will be 3907 units cells in one average particle. The cross section of one unit cell is 0.877 nm^2 and the surface area of an average cylindrical nanoparticle is 1413.4 nm^2. So, there will be 1611 cells exposed on surface. Thus, it is calculated that La dispersion is 0.412.

We cooperate this value to the TOF calculation and the updated TOF figure is given below:

In manuscript on page 16 line 10

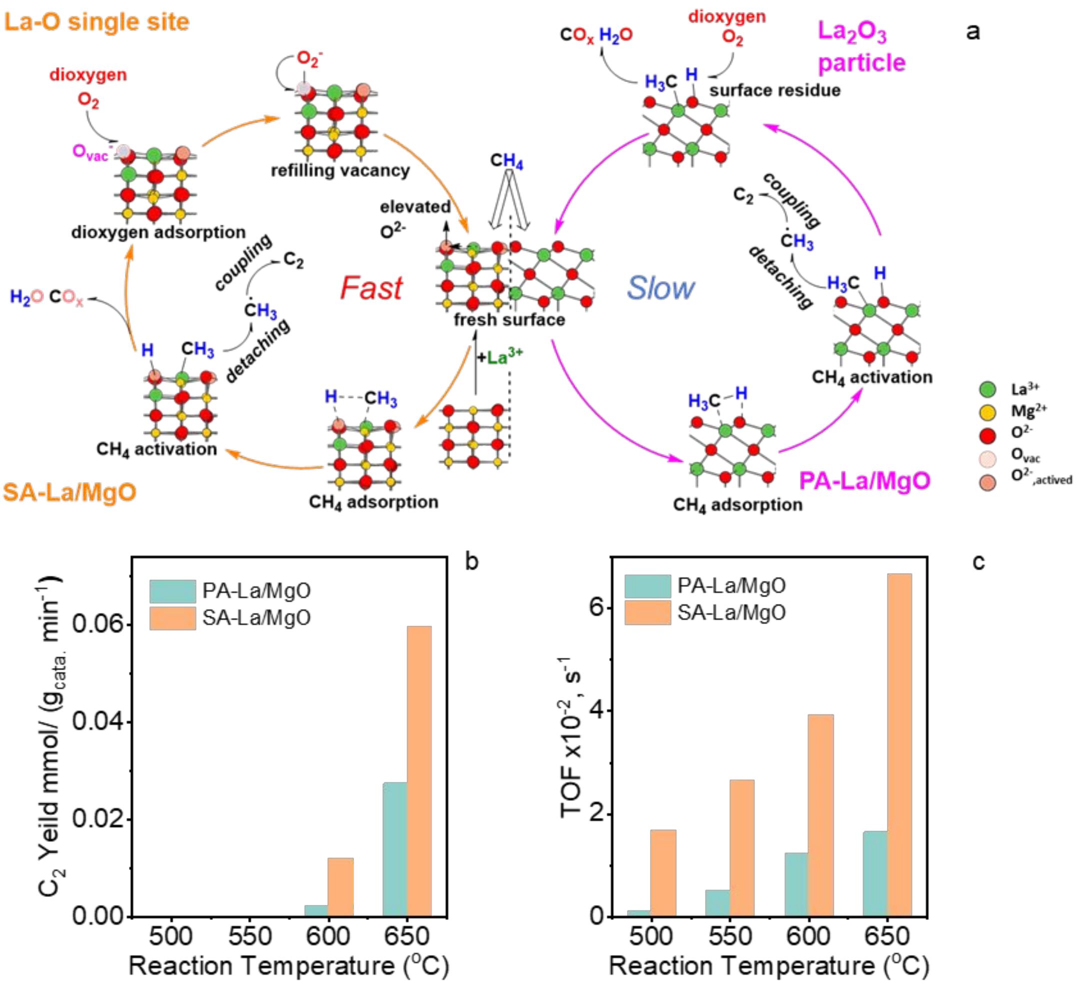

“

Scheme 1 Proposed OCM over SA-La/MgO and PA-La/MgO. Where green balls represent La, red balls represent O, pink balls represent activated surface lattice O^{2-}_{act} in La-O-Mg slingshot site, and yellow balls represent Mg (a); C_2 yield of PA-La/MgO (b) and the apparent TOF of PA-La/MgO and SA-La/MgO (c)”

The following content has been edited in manuscript:

In manuscript on page 17 line 22:

“*which is around 4 times higher (6.39×10^{-2} vs. 1.65×10^{-3} @ 650 °C) than the over PA-La/MgO, indicating the excellent activity and C_2 selectivity of this active site.*”

We’d also like to acknowledge the reviewer’s comment regarding to comparing the sample with 1wt% La loading amount. Our initial motivate to synthesis 5% La/MgO is to show the distinct difference between atomically dispersed La on MgO and La_2O_3 nanoparticle on MgO. However,

as pointed out by reviewer, using same loading amount might be a better approach and we will follow this instruction in our future research. For the current project, we synthesized the 1wt% loaded La/MgO via impregnation method and the apparent activation energy for both PA-LaMgO (97.9±18.0 kJ/mol) and 1wt%La/MgO via impregnation (88.1±6.9 kJ/mol) are comparable. So 1wt% La/MgO has same active sites compared to reported PALaMgO in this work, and the latter is representative and can effectively reflect the properties of La_2O_3/MgO catalyst during the OCM reaction.

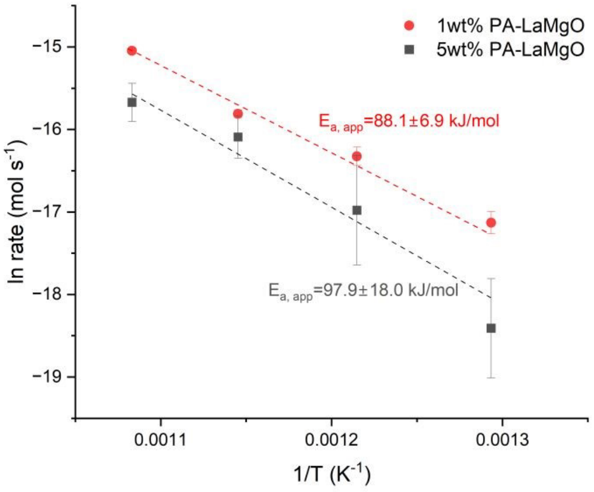

11. The catalytic activity should be benchmarked against literature values to properly contextualize the reported performance.

Response: We acknowledge the reviewer’s comment about displaying literature reported OCM performance. The following content has been added:

In supporting information, on page S2 line 1“

Table S1 Selected OCM performance data reported for La-promoted MgO and intrinsic La_2O_3.

Entry	*Catalyst*	*La/Mg molar ratio*	*Reaction temperature (°C)*	*Molar ratio CH_4/O_2*	*Space Velocity (ml/g/hr)*	*CH4 conversion (%)*	*C2 selectivity (%)*	*References*
1	*La-MgO*	*0.1*	*800*	*3.2*	*18,000*	*27.8*	*40.5*	*[1]*
2	*La-MgO*	*0.1*	*800*	*4*	*102,000*	*24.7*	*63.5*	*[2]*
3	*La-MgO*	*0.005*	*700*	*4*	*51,600*	*25.8*	*46.6*	*[3]*
			800	*4*	*51,600*	*30.9*	*59.6*	
4	*La/MgO*	*0.11*	*700*	*4*	*51,600*	*24.8*	*34.1*	*[3]*
			800	*4*	*51,600*	*29.5*	*53.5*	
5	*La-MgO*	*0.02*	*650*	*4*	*51,360*	*1.9*	*62.6*	*[4]*
6	*La2O3*	*N/A*	*800*	*4*	*102,000*	*25.7*	*57.6*	*[2]*
7	*La2O3*	*N/A*	*800*	*3*	*12,000*	*28.4*	*36.7*	*[5]*
8	*La2O3*	*N/A*	*600*	*3*	*60,000*	*14.7*	*23.7*	*[6]*

”

The following content has been edited in manuscript, on page 2 line 21:

"*yet their performance (Table S1) is still constrained by the intrinsic challenge of balancing methane conversion with selectivity*"

12. Details about the CrystalMaker modeling and construction parameters are missing and should be provided for reproducibility.

Response: We appreciate the reviewer's comment for providing details regarding CrystalMaker simulation. As suggested by reviewer, the detailed step added into the supporting information

In supporting information on page S6 line 13:

"*CrystalMaker simulation:*

The simulation was carried out in CrystalMaker® 11 software. The MgO crystal structure was obtained from ICSD database (ICSD#9863). The MgO size was 2x2x2 cells and the structure was transferred from crystal to molecule before substitute one surface Mg cation by La cation. Then the whole structure underwent relaxation until the stable energy level of the crystal structure was reached. "

13. Minor corrections needed: a) Page 10 line 17 contains punctuation errors; b) The reference to "Figure 3a" on page 10 line 23 should be "Figure 3c".

Response: We appreciate the comments from reviewer about some minor corrections and apologise for our oversight in the original draft preparation. The punctuation error has been fixed. The labelling issue also fixed as follows:

In manuscript on page 12 line 6:

"*as depicted by the blue line in Figure 5c*"

14. The proposed surface site properties and elementary reaction steps would benefit significantly from supporting DFT calculations to provide theoretical validation of the experimental observations.

Response: We sincerely appreciate the reviewer's insightful comments regarding the DFT calculations. In this study, our primary focus is on employing advanced experimental techniques to investigate the OCM reaction over the La/MgO system and elucidating the structure-function relationship of the active sites. The in situ DRIFTS results reveal the formation of key intermediates on SA-LaMgO under various gas environments at elevated temperatures. Complementarily, the in situ ETEM results demonstrate the dynamic structural change of the material, while the in situ EELS measurements capture the chemical environmental evolution of the La-O-Mg sites during the OCM reaction. Based on this comprehensive set of experimental evidence, we have proposed our reaction mechanism.

We acknowledge the reviewer's valuable suggestion that incorporating theoretical calculations to experimental observations. However, due to current time and resource constraints, we were unable to perform DFT calculations in the present work. We recognize the importance of this approach and will actively seek collaborations with computational groups to integrate theoretical insights into our further studies.

References

[1] A. Monshi, M.R. Foroughi, M.R. Monshi, Modified Scherrer equation to estimate more accurately nano-crystallite size using XRD, World journal of nano science and engineering, 2 (2012) 154-160.

[2] J.P.H. Li, X. Zhou, Y. Pang, L. Zhu, E.I. Vovk, L. Cong, A.P. van Bavel, S. Li, Y. Yang, Understanding of binding energy calibration in XPS of lanthanum oxide by in situ treatment, Physical Chemistry Chemical Physics, 21 (2019) 22351-22358. [3] X. Chen, R. Ye, C. Jin, F. Hu, L. Zhou, Z.-H. Lu, R. Zhang, G. Feng, A highly efficient Ni/3DOM-$La_2O_2CO_3$ catalyst with ordered macroporous structure for CO_2 methanation, Journal of Catalysis, 428 (2023) 115129.

[4] X. Shao, S. Prada, L. Giordano, G. Pacchioni, N. Nilius, H.-J. Freund, Tailoring the Shape of Metal Ad-Particles by Doping the Oxide Support, Angewandte Chemie International Edition, 50 (2011) 11525-11527.

[5] N. Mammen, S. Narasimhan, S. de Gironcoli, Tuning the Morphology of Gold Clusters by Substrate Doping, Journal of the American Chemical Society, 133 (2011) 2801-2803.

[6] Y. Cui, X. Shao, S. Prada, L. Giordano, G. Pacchioni, H.-J. Freund, N. Nilius, Surface defects and their impact on the electronic structure of Mo-doped CaO films: an STM and DFT study, Physical Chemistry Chemical Physics, 16 (2014) 1276412772.

[7] J.B. Pedley, E.M. Marshall, Thermochemical Data for Gaseous Monoxides, Journal of Physical and Chemical Reference Data, 12 (1983) 967-1031.

[8] Z.-Q. Wang, D. Wang, X.-Q. Gong, Strategies To Improve the Activity While Maintaining the Selectivity of Oxidative Coupling of Methane at La2O3: A Density Functional Theory Study, ACS Catalysis, 10 (2020) 586-594.
[9] R. Shannon, Revised effective ionic radii and systematic studies of interatomic distances in halides and chalcogenides, Acta Crystallographica Section A, 32 (1976) 751-767.
[10] K. Exner, P.v.R. Schleyer, Theoretical Bond Energies: A Critical Evaluation, The Journal of Physical Chemistry A, 105 (2001) 3407-3416.
[11] D. Kiani, S. Sourav, J. Baltrusaitis, I.E. Wachs, Oxidative Coupling of Methane (OCM) by SiO2-Supported Tungsten Oxide Catalysts Promoted with Mn and Na, ACS Catalysis, 9 (2019) 5912-5928.
[12] A. Gloter, C. Ewels, P. Umek, D. Arcon, C. Colliex, Electronic structure of titaniabased nanotubes investigated by EELS spectroscopy, Physical Review B, 80 (2009) 035413.
[13] G. Bertoni, L. Calmels, A. Altibelli, V. Serin, First-principles calculation of the electronic structure and EELS spectra at the graphene/Ni(111) interface, Physical Review B, 71 (2005) 075402.
[14] M. Chen, A.R. Felmy, D.A. Dixon, Structures and Stabilities of (MgO)n Nanoclusters, The Journal of Physical Chemistry A, 118 (2014) 3136-3146. [15] X.-L. Ding, Z.-Y. Li, J.-H. Meng, Y.-X. Zhao, S.-G. He, Density-functional global optimization of $(La_2O_3)_n$ clusters, The Journal of Chemical Physics, 137 (2012) 214311.
[16] W. Adi, S. Wardiyati, S. Dewi, Nanoneedles of lanthanum oxide (La_2O_3): a novel functional material for microwave absorber material, IOP Conference Series: Materials Science and Engineering, IOP Publishing, 2017, pp. 012066.

oc-2025-010165.R2

Name: Peer Review Information for "Active Slingshot Geometry Site on Single-Atom La Catalyst Largely Promotes Oxidative Methane Coupling"

Second Round of Reviewer Comments

Reviewer: 2

Comments to the Author

The authors have made substantial efforts to address the initial review comments, incorporating significant improvements such as new iDPC-STEM data confirming the

"slingshot" geometry, quantitative analysis of oxygen vacancy dynamics under alternating gas environments. These revisions strengthen the mechanistic interpretation of the La-O-Mg active site. While the manuscript is now considerably improved and potentially suitable for publication, three issues require resolution before final acceptance:

1. The Arrhenius plots in Figure S7 may be influenced by equilibrium limitations at higher temperatures (especially for intrinsic MgO and PA-La/MgO). To ensure the reported Ea values solely reflect kinetic barriers, Figure 1g must be supplemented with calculated equilibrium methane conversion curves under the experimental conditions. The authors must explicitly state whether the measured conversions (particularly at T ≥ 650°C) are significantly below equilibrium or if the derived Ea values could be affected by approaching equilibrium, necessitating a discussion of this potential limitation.

2. The claim of lattice expansion around the La site (3.6 Å vs. 2.9 Å bulk MgO distance in Fig. 2b) is central to the "slingshot" model. However, comparison to the undoped MgO (100) surface termination is essential. The authors must provide atomic-resolution iDPC-STEM or HAADF-STEM data of pristine MgO (equivalent to Fig. 1a but at the surface sensitivity of Fig. 2b/S8) to directly measure the inherent surface Mg-Mg distance. This baseline measurement is critical to confirm that the observed 3.6 Å spacing is indeed induced by La doping and not a general surface relaxation phenomenon of MgO.

3. Despite corrections, several key figure citations remain incorrect, such as Page 14, line 2: "Figure 4h", Page 14, line 9: "Figure 3c", Page 15, line 10: "Figure 4h" etc. The authors must perform a meticulous, final check of all figure citations in the main text and Supporting Information to eliminate these errors, which hinder the reader's ability to follow the evidence.

Reviewer: 1

Comments to the Author

I have carefully reviewed the responses, and all comments have been thoroughly addressed. The supplementary experiments provided by the authors significantly enhance

the persuasiveness of the reported phenomenon. Therefore, I recommend its publication in ACS Central Science without further change.

Author's Response to Peer Review Comments:

Dear Editor, and colleagues of ACS Central Science's editor office,

many thanks for handling our manuscript entittled "Active Slingshot Geometry Site on Single-Atom La Catalyst Largely Promotes Oxidative Methane Coupling".

We appreciate the questions raised from reviewers and we have revised our manuscript accordingly.

We hope our reply could address all the issues.

Please find the point by point reply in the attached file.

Best

Ang

Reviewer(s)' Comments to Author:

Reviewer: 2

Recommendation: Publish in ACS Central Science after minor revisions noted.

Comments:

The authors have made substantial efforts to address the initial review comments, incorporating significant improvements such as new iDPC-STEM data confirming the "slingshot" geometry, quantitative analysis of oxygen vacancy dynamics under alternating gas environments. These revisions strengthen the mechanistic interpretation of the La-O-Mg active site. While the manuscript is now considerably improved and potentially suitable for publication, three issues require resolution before final acceptance:

Response: We sincerely appreciate the reviewer’s constructive comments during the firstround revision, which have greatly contributed to improving the quality of our manuscript. We are also grateful for the reviewer’s positive feedback on our work. All the issues raised have been carefully addressed point by point as follows:

1. The Arrhenius plots in Figure S7 may be influenced by equilibrium limitations at higher temperatures (especially for intrinsic MgO and PA-La/MgO). To ensure the reported Ea

values solely reflect kinetic barriers, Figure 1g must be supplemented with calculated equilibrium methane conversion curves under the experimental conditions. The authors must explicitly state whether the measured conversions (particularly at T ≥ 650°C) are significantly below equilibrium or if the derived Ea values could be affected by approaching equilibrium, necessitating a discussion of this potential limitation.

Response: We sincerely thank the reviewer's comment about equilibrium limitation. Indeed, it is important to make sure the conversion is less than equilibrium conversion so that the reaction is kinetic controlled and the Ea value would reflect the reaction kinetic. Here, we use the reaction from CH_4 to C_2H_4 and C_2H_6 as examples to explain why the CH_4 current conversion is significantly lower than equilibrium condition.

According to fundamental thermodynamic relation between equilibrium constant and Gibbs free energy

$$\Delta G = -RT ln K$$

Where ΔG is Gibbs free energy of the reaction, R is the universal gas constant (8.314 J mol^{-1} K^{-1}), T is Temperature (K), and K is equilibrium constant.

Also, Gibbs free energy ΔG is equals to

$$\Delta G = \Delta H - T\Delta S$$

Where ΔH is enthalpy change of the reaction while ΔS is the entropy change of the reaction.

For the reaction

$4CH_4 + O_2 = 2C_2H_6 + 2 H_2O$

$$K_{C2H6} = \frac{[C_2H_6]^2[H_2O]^2}{[CH_4]^4[O_2]}$$

The thermodynamic parameters' value at reaction condition are:

Temperature (°C)	ΔH (kJ)	ΔS (J/K)	ΔG (kJ)	Equilibrium constant K
300.000	-352.782	-109.663	-289.929	2.662×10^{26}
350.000	-352.381	-108.992	-284.463	7.026×10^{23}
400.000	-352.020	-108.433	-279.028	4.504×10^{21}

450.000	-351.710	-107.989	-273.618	5.829×10^{19}
500.000	-351.459	-107.652	-268.227	1.328×10^{18}
550.000	-351.274	-107.421	-262.851	4.799×10^{16}
600.000	-351.162	-107.288	-257.483	2.540×10^{15}
650.000	-351.124	-107.246	-252.120	1.849×10^{14}
700.000	-351.158	-107.281	-246.758	1.762×10^{13}

According to these thermodynamic parameters, the equilibrium constant K is 1.849×10^{14} at 650 ^{o}C, indicating all of the CH_4 will be converted at equilibrium condition.

Similarly, we tested the reaction

$2CH_4+O_2=C_2H_4+2H_2O$

$$K_{C2H4}=\frac{[C_2H_4][H_2O]^2}{[CH_4]^2[O_2]}$$

The thermodynamic parameters' value at reaction condition are:

**Temperature (^{o}C)**	**ΔH (kJ)**	**ΔS (J/K)**	**ΔG (kJ)**	**Equilibrium constant K**
300.000	-279.520	25.216	-293.972	6.219×10^{26}
350.000	-279.221	25.716	-295.246	5.632×10^{24}
400.000	-278.999	26.061	-296.541	1.030×10^{23}
450.000	-278.855	26.267	-297.850	3.282×10^{21}
500.000	-278.793	26.351	-299.166	1.635×10^{20}
550.000	-278.808	26.332	-300.483	1.173×10^{19}
600.000	-278.899	26.226	-301.798	1.138×10^{18}
650.000	-279.059	26.047	-303.105	1.419×10^{17}
700.000	-279.285	25.810	-304.401	2.190×10^{16}

According to these thermodynamic parameters, the equilibrium constant K is 1.419×10^{17} at 650 °C, indicating all of the CH_4 will be converted at equilibrium condition.

These calculations exhibit extremely high equilibrium constant K between 300 °C to 700 °C, proofed that equilibrium CH_4 conversion is 100% between this temperature range. In our measurement, the CH_4 conversion at 650 °C is less than 10%, which is significantly lower than the equilibrium conversion of CH_4 at this condition. Thus, we are confident that our Ea values reflects the kinetic properties of the catalyst rather than influence equilibrium limitation. Due to the huge differences between equilibrium conversion and measurement conversion, adding equilibrium conversion curve will change vertical axis scale, thereby making Figure 1g difficult to interpret. Thus, we may not adding this equilibrium curve into Figure 1g.

2. The claim of lattice expansion around the La site (3.6 Å vs. 2.9 Å bulk MgO distance in Fig. 2b) is central to the "slingshot" model. However, comparison to the undoped MgO (100) surface termination is essential. The authors must provide atomic-resolution iDPC-STEM or HAADF-STEM data of pristine MgO (equivalent to Fig. 1a but at the surface sensitivity of Fig. 2b/S8) to directly measure the inherent surface Mg-Mg distance. This baseline measurement is critical to confirm that the observed 3.6 Å spacing is indeed induced by La doping and not a general surface relaxation phenomenon of MgO.

Response: We sincerely appreciate the comments from reviewer regarding to providing the electron microscope image of MgO at the surface sensitivity. As suggested by reviewer we added the HAADF-STEM image showing the surface of intrinsic MgO. The HAADF-STEM image indicates no La-O-Mg "Slingshot" structure was observed on intrinsic MgO, indicating the unique La-O-Mg "Slingshot" structure is originated from the effect of La^{3+} cation.

To address this issue, the following content has been added in manuscript in page 9 line 5

"In contrast, as shown in Figure S9, intrinsic MgO exhibited a stable surface without the emergence of the 'slingshot' geometry, further confirming that this unique structure originates from the effect of La^{3+}.

"

The following content has been added in the supporting information in page S11 line 1

“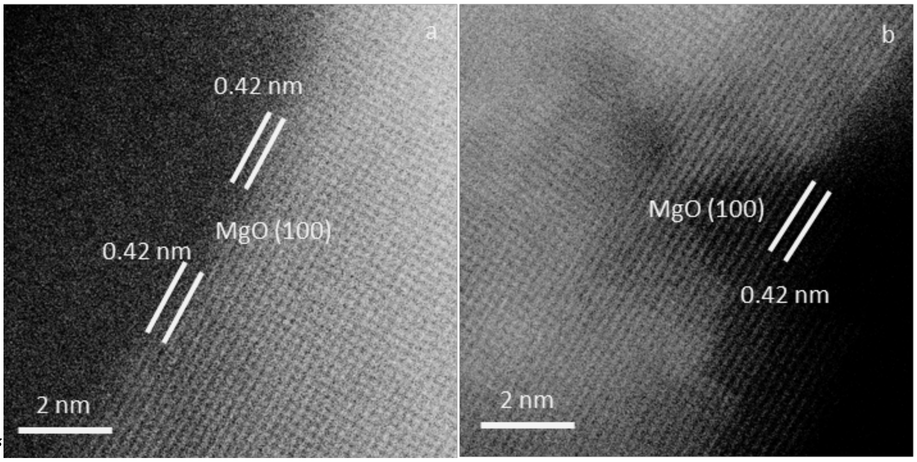

Figure S9 HAADF-STEM image of intrinsic MgO surface (a) and the HAADF-STEM image of intrinsic MgO surface at another site (b). “Slingshot structure” was not detected in the intrinsic MgO.”

3. Despite corrections, several key figure citations remain incorrect, such as Page 14, line 2: "Figure 4h", Page 14, line 9: "Figure 3c", Page 15, line 10: "Figure 4h" etc. The authors must perform a meticulous, final check of all figure citations in the main text and Supporting Information to eliminate these errors, which hinder the reader's ability to follow the evidence.

Response: We sincerely thank the reviewer’s comment regarding to the incorrect editing issue and we’d also apologize for our negligence. We’ve double checked the manuscript and supporting information to eliminate these errors. All of the corrections are highlighted in red typeface.

Reviewer: 1

Recommendation: Publish in ACS Central Science without change.

Comments: I have carefully reviewed the responses, and all comments have been thoroughly addressed.

Response: We sincerely thank the reviewer for their valuable comments and suggestions, which have greatly helped us to improve the quality of our work.
